# Lipidomic profiling reveals distinct differences in plasma lipid composition in healthy, prediabetic, and type 2 diabetic individuals

**DOI:** 10.1093/gigascience/gix036

**Published:** 2017-05-15

**Authors:** Huanzi Zhong, Chao Fang, Yanqun Fan, Yan Lu, Bo Wen, Huahui Ren, Guixue Hou, Fangming Yang, Hailiang Xie, Zhuye Jie, Ye Peng, Zhiqiang Ye, Jiegen Wu, Jin Zi, Guoqing Zhao, Jiayu Chen, Xiao Bao, Yihe Hu, Yan Gao, Jun Zhang, Huanming Yang, Jian Wang, Lise Madsen, Karsten Kristiansen, Chuanming Ni, Junhua Li, Siqi Liu

**Affiliations:** 1 BGI-Shenzhen, Shenzhen 518083, China; 2 China National GeneBank-Shenzhen, BGI-Shenzhen, Shenzhen 518083, China; 3 Shenzhen Key Laboratory of Human Commensal Microorganisms and Health Research, BGI-Shenzhen, Shenzhen 518083, China; 4 Shenzhen Engineering Laboratory of Detection and Intervention of Human Intestinal Microbiome, BGI-Shenzhen, Shenzhen 518083, China; 5 Suzhou Center for Disease Prevention and Control, Suzhou 215007, China; 6 Proteomics Division, BGI-Shenzhen, Shenzhen 518083, China; 7 BGI Education Center, University of Chinese Academy of Sciences, Beijing, China; 8 School of Bioscience and Bioengineering, South China University of Technology, Guangzhou, China; 9 James D. Watson Institute of Genome Sciences, Hangzhou 310058, China; 10 Laboratory of Genomics and Molecular Biomedicine, Department of Biology, University of Copenhagen, 2100 Copenhagen Ø, Denmark; 11 National Institute of Nutrition and Seafood Research (NIFES), 5817 Bergen, Norway; 12 CAS Key Laboratory of Genome Sciences and Information, Beijing Institute of Genomics, Chinese Academy of Sciences, Beijing, China

**Keywords:** lipidomics, type 2 diabetes, prediabetes, plasma

## Abstract

The relationship between dyslipidemia and type 2 diabetes mellitus (T2D) has been extensively reported, but the global lipid profiles, especially in the East Asia population, associated with the development of T2D remain to be characterized. Liquid chromatography coupled to tandem mass spectrometry was applied to detect the global lipidome in the fasting plasma of 293 Chinese individuals, including 114 T2D patients, 81 prediabetic subjects, and 98 individuals with normal glucose tolerance (NGT). Both qualitative and quantitative analyses revealed a gradual change in plasma lipid features with T2D patients exhibiting characteristics close to those of prediabetic individuals, whereas they differed significantly from individuals with NGT. We constructed and validated a random forest classifier with 28 lipidomic features that effectively discriminated T2D from NGT or prediabetes. Most of the selected features significantly correlated with diabetic clinical indices. Hydroxybutyrylcarnitine was positively correlated with fasting plasma glucose, 2-hour postprandial glucose, glycated hemoglobin, and insulin resistance index (HOMA-IR). Lysophosphatidylcholines such as lysophosphatidylcholine (18:0), lysophosphatidylcholine (18:1), and lysophosphatidylcholine (18:2) were all negatively correlated with HOMA-IR. The altered plasma lipidome in Chinese T2D and prediabetic subjects suggests that lipid features may play a role in the pathogenesis of T2D and that such features may provide a basis for evaluating risk and monitoring disease development.

## Background

Type 2 diabetes mellitus (T2D) is a progressive and complex disease that is tightly associated with heterogeneous metabolic disorders, particularly in glucose and lipid metabolism [1]. The prevalence of prediabetes, defined by blood glucose levels between normal and diabetic levels, is increasing rapidly worldwide. Hence, characterization of abnormalities in glucose and lipid metabolism at the prediabetic state is warranted. The high prevalence of prediabetes and T2D is rapidly increasing in highly populated countries like China. Using criteria defined by the World Health Organization (WHO) and American Diabetes Association, 2 national epidemiological studies reported that the prevalence of adult diabetes in China was 9.7% in 2007 and 11.6% in 2010, respectively, whereas the prevalence of adult prediabetes ranged from 15.5% to 50.1% [2, 3].

As a strong association between T2D and dysregulation of lipid metabolism is well established [1], metabolomics techniques, especially lipidomics, represent powerful tools to globally survey metabolites associated with prediabetes and T2D. Further, metabolomic analyses may provide insight into ongoing biochemical processes and identify biomarkers to predict disease risk. In a longitudinal study comprising 2422 normoglycemic individuals followed for 12 years, plasma levels of 3 branched-chain amino acids (BCAA; isoleucine, leucine, valine) and 2 aromatic amino acids (tyrosine and phenylalanine) exhibited highly significant associations with the future development of diabetes [4]. By comparing metabolomic profiles of obese versus lean humans, Newgard et al. have further revealed a BCAA-related metabolite signature that correlates with insulin resistance, and the concomitant specific increases in C3 and C5 acyl-carnitine levels suggested increased catabolism of BCAA [5]. Rat studies based on these findings demonstrated that supplementation of a high-fat diet with BCAA caused insulin resistance despite reduced food intake and body weight [5]. Compared with lean subjects, obese nondiabetic and T2D subjects exhibit increased levels of long-chain acyl-carnitines, suggesting impairment of entry of fatty acids into mitochondria [6]. Additionally, T2D adults of comparable body mass index (BMI) exhibit increased levels of several short- and medium-chain acyl-carnitines, suggesting that diabetic subjects generally suffer from a complex oxidation defect [6]. The strong association between prediabetes, T2D, and dysregulation of lipid metabolism is further supported by the plasma profiling of 117 T2D, 64 prediabetes, and 170 normal glucose tolerant (NGT) participants using targeted lipidomics [7]. This study revealed that over 100 individual lipid species, including sphingolipids, phospholipids, glycerolipids, ceramides, and cholesterol esters, were tightly associated with T2D and prediabetes [7]. Additionally, T2D risk classification models of potential value for clinical use have been developed and evaluated by Meikle and coworkers to stratify individuals with a fasting plasma glucose (FPG) < 6.1 mmol/L using lipidomics profiles with or without non-lipid risk factors [8]. Non-targeted lipidomics further represents a tool to discover metabolic regulatory networks and thereby increases our understanding of lipid metabolism in the pathophysiology of metabolic diseases including T2D [9]. Of note, it has been reported that East and South Asians develop T2D at a lower mean BMI compared to the Western population [10], and Asians have more body fat and a higher tendency to visceral adiposity for a given BMI than the Western diabetes population [10]. Hence, characterizing the T2D lipid profile in an Asian population (represented by Chinese) could help fill the gaps in understanding these interethnic differences.

The conventional data-dependent acquisition (DDA) mass spectrometry (MS) mode has been widely used for lipidomics in most laboratories, in which the detection parameters, such as the number of precursor ions selected per cycle and dynamic exclusion to minimize repeat precursor ions, can be optimized to identify complex lipid molecules [11]. DDA performance, however, has several inherent limitations, such as limited dynamic range, a bias toward high-abundance ions, and a long duty cycle accompanying increase in sample complexity. The strategy of data-independent acquisition (DIA) has recently been developed to alleviate these limitations [12, 13]. By collecting all fragment ions simultaneously without any preselection, DIA could thus enable in-depth analysis for both qualification and quantification of lipids with improved detection sensitivity and analysis reproducibility. However, the DIA method is not easily applicable in lipidomics as the annotation and false discovery rate (FDR) evaluation of MS features in large complex lipid datasets require more sophisticated software and an integrated reference database [14].

Here, we conducted a global lipidomics analysis of 293 Chinese individuals, including 114 T2D patients, 81 prediabetic subjects, and 98 individuals with NGT, using DIA-based liquid chromatography–tandem mass spectrometry (LC-MS/MS). Using commercial and in-house software to analyze the highly complex dataset, we demonstrate that Chinese T2D patients possessed significant lipid changes in plasma compared with prediabetic and NGT individuals. Further, we identified several lipid features, including lysophosphatidylcholine (lysoPC) and acylcarnitine species, that are potential indicators for predicting diabetic risk.

## Data Description

To delineate global lipidomic profiles in Chinese prediabetic and T2D diabetic patients, fasting blood samples together with the corresponding clinical and phenotypic data were collected from 114 T2D patients, 81 prediabetic individuals, and 98 NGT individuals in Suzhou, Jiangsu Province, China (Additional file 1). Lipids were extracted from individual plasma samples and then injected on the Waters XEVO-G2XS-QTOF instrument in both positive and negative modes, with pooled extraction quality control (QC) samples at certain intervals (Additional file 2). The raw datasets were subjected to nonlinear alignment and normalization by the commercial software Progenesis QI 2.0 and further analyzed by the in-house pipeline metaX [15] to generate lipid profiles. Univariate and multivariate analyses were conducted using R statistics software to identify and evaluate the significant lipidomic features among the groups.

### Analyses

**Figure 1:**
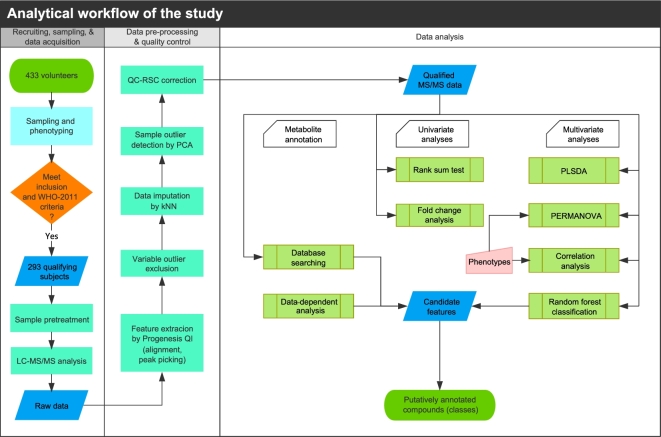
Flowchart for participant recruitment and data processing. The recruitment of participants was based on the 2011 World Health Organization criteria for diabetes and prediabetes diagnoses. Blood and clinical data were acquired from 293 qualifying subjects, and untargeted lipidomics liquid chromatography–tandem mass spectrometry analysis was performed. The raw data were preprocessed with Progenesis QI 2.0 to extract metabolic features. Unqualified variables and samples were detected and discarded using the BGI in-house program metaX [15]. Several types of statistical analyses such as rank sum tests, fold change analysis, partial least squares discriminant analysis, and random forest analysis were performed to identify metabolites that differed significantly between the diagnostic groups. The lipid compounds selected by the RF classifiers were identified by matching their accurate masses and mass spectrometry^E^ ion spectral fragmentation patterns to those in the database. Data-dependent acquisition analysis was applied to improve the resolving power for identification. See the Methods for more details.

The 3 phases of the integrated workflow for this study are illustrated in Fig. 1: phase 1, collecting information of clinical specimens and acquiring mass signals from LC-MS/MS; phase 2, extracting metabolic features through the software of MS data analysis; and phase 3, identifying lipid candidates in response to the development of T2D.

### Assessment of clinical characteristics and plasma lipidomic features

The clinical information including physiological and biochemical parameters of the individuals included in the cohort is summarized in Table 1. The levels of FPG, 2-hour postprandial glucose (2h-PG), glycated hemoglobin (HbA1c), fasting C-peptide, and insulin resistance index (HOMA-IR) were significantly higher in both T2D patients and prediabetic individuals than in NGT individuals, with T2D patients exhibiting higher values compared with prediabetic individuals (Dunn's post hoc test, *P* < 0.05). T2D patients had higher age, BMI, waist–hip ratio, systolic blood pressure (SBP), triglyceride, total cholesterol (TC), and low-density lipoprotein (LDL) levels than NGT individuals (Dunn's post hoc test, *P* < 0.05). Further, the proportion of people taking calcium channel blockers (CCBs) for hypertension was higher in T2D patients than in prediabetic and NGT individuals (chi-square test, *P* < 0.05).

**Table 1:** Baseline characteristics in 3 groups of the study.

	T2D	Pre-DM*	NGT		T2D vs	T2D vs	Pre-DM
Variables	(*n* = 114)	(*n* = 81)	(*n* = 98)	*P*-value^a^	Pre-DM^c^	NGT^c^	vs NGT^c^
Gender (female), no. (%)	68 (59.65)	40 (49.38)	66 (67.35)	0.0513			
Smoking, no. (%)	18 (15.80)	24 (29.63)	17 (17.35)	0.068			
Hypertension, no. (%)	52 (45.61)	38 (46.91)	21 (21.43)	0.0002			
CCB use, no. (%)	29 (25.43)	20 (24.69)	12 (12.24)	0.0372			
Alcohol drinking, no. (%)	12 (10.53)	19 (23.46)	12 (12.24)	0.0769			
				*P-*value^b^			
Age, year	65.11 ± 8.77	61.99 ± 8.48	59.11 ± 9.15	4.52E–06	0.0356	4.81E–06	0.026
BMI	25.25 ± 3.14	25.23 ± 3.13	24.23 ± 3.26	0.0425	0.9965	0.0569	0.0569
Waist–hip ratio	0.92 ± 0.06	0.91 ± 0.06	0.89 ± 0.06	0.0166	0.6237	0.0291	0.0846
FPG, mmol/l	7.87 ± 1.99	5.91 ± 0.62	5.34 ± 0.36	2.20E–16	1.47E–13	3.49E–37	4.30E–06
2h-PG, mmol/l	15.1 ± 3.76	8.21 ± 1.63	6.01 ± 1.01	2.20E–16	3.23E–13	6.41E–41	1.30E–08
HbA1c, %	7.51 ± 2.06	5.51 ± 0.54	5.04 ± 0.42	2.20E–16	4.87E–15	5.19E–38	1.64E–05
Insulin, ulU/ml	8.85 ± 3.51	8.27 ± 3.75	7.39 ± 2.93	0.0017	0.2406	0.0004	0.0263
C-peptide, ng/ml	2.31 ± 0.99	2.07 ± 0.85	1.75 ± 0.69	4.55E–05	0.1524	1.70E–05	0.0079
HOMA-IR	3.13 ± 1.57	2.17 ± 1.03	1.76 ± 0.74	2.20E–16	3.64E–07	1.80E–21	0.0001
SBP, mm Hg	136.57 ± 24.26	130.91 ± 15.73	123.28 ± 16.81	3.69E–08	0.0129	9.75E–09	0.0036
DBP, mm Hg	79.73 ± 12.21	80.37 ± 8.12	77.35 ± 9.33	0.0345	0.6724	0.0653	0.0653
TG, mmol/l	2.04 ± 1.55	1.92 ± 1.19	1.55 ± 0.91	0.0051	0.6202	0.0149	0.0131
CHO, mmol/l	5.43 ± 1.39	5.22 ± 1.18	5.17 ± 1.43	0.2418	0.2083	0.2083	0.9262
LDL, mmol/l	3.86 ± 3.36	3.17 ± 2.00	2.56 ± 1.21	0.0014	0.2047	0.0005	0.0386
HDL, mmol/l	1.16 ± 0.35	1.14 ± 0.37	1.23 ± 0.32	0.2891	0.2225	0.6406	0.2029
Leptin, ng/ml	5.09 ± 1.91	4.29 ± 2.12	4.56 ± 1.67	0.0066	0.0013	0.0281	0.2107
GAD-Ab, lU/ml	13.66 ± 14.61	14.05 ± 13.14	12.92±17.45	0.066	0.3562	0.3173	0.1236
HsCRP, mg/l	2.62 ± 2.41	2.17 ± 1.81	2.16 ± 1.84	0.4485	0.7873	0.7873	0.7873
Adiponectin, ng/ml	37.41 ± 13.53	37.91 ± 16.57	39.2 ± 13.18	0.5321	0.6446	0.5684	0.5684

Values are given as mean ± SD or number of individuals (%).

*The prediabetes (Pre-DM) group consisted of 7 isolated impaired fasting glucose (iIFT), 35 isolated impaired glucose tolerance (iIGT), 24 combined IFG/IGT, and 15 raised HbA1c.

^a^ *P*-value of chi-square test.

^b^ *P*-value of Kruskal–Wallis test.

^c^ *P*-value of Dunn's post hoc test.

We assessed both coverage and reproducibility of the non-targeted lipidomic data on our large sample cohorts. Using Progenesis QI 2.0 and metaX, the untargeted lipidomic analysis yielded 11 077 features, with an average coefficient of variation (CV) of 14.8% in the positive ion mode (PIM) and 923 features with an average CV of 14.7% in the negative ion mode (NIM) after strict quality control (Additional file 3). Of the features in PIM, approximately 46.77% (5181/11 077) were matched to 1 or more molecular species with characteristic compatible with lipids or lipid-like compounds, whereas the corresponding distribution in NIM was 36.72% (339/923). Principal component analysis (PCA) showed that all of the QC samples spiked at certain intervals clustered together, verifying an acceptable reproducibility and stability of the results (Additional file 4). Twelve positive and 9 negative datasets were considered outliers and were removed (Additional file 4A and B).

The interactions between the clinical parameters (Additional file 1) and the global lipid profiles, obtained by combining datasets from both ion modes (Additional file 3), were further evaluated by permutational multivariate analysis of variance (PERMANOVA). The lipidomic datasets were significantly associated with several plasma indices such as triglyceride, high-density lipoprotein, TC, leptin, C-peptide, and HOMA-IR index, and with physiological conditions, such as gender, waist–hip ratio, BMI, and age (FDR < 0.05) (Additional file 5). Weak interactions were also observed between the global lipid profiles and other clinical parameters such as levels of fasting insulin, FPG, LDL, SBP, and CCB treatment (FDR > 0.05) (*P* < 0.05).

### Prediabetes and T2D-related lipidomic features

**Figure 2:**
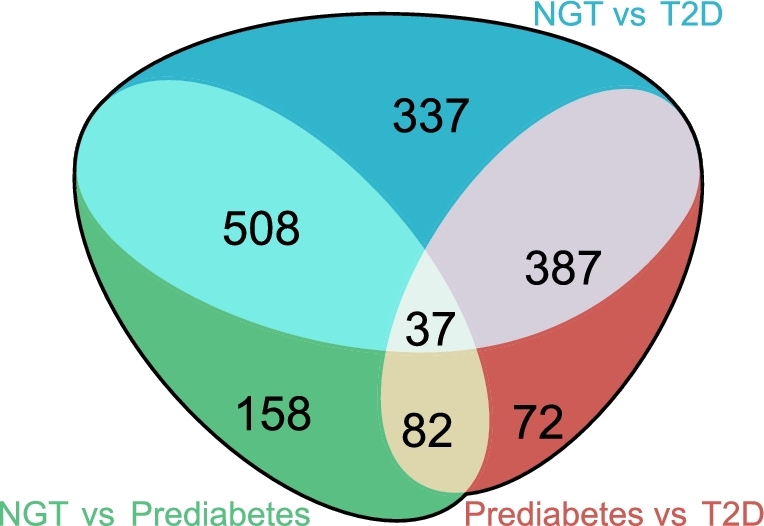
Venn diagram of significant metabolites from the 3 pairwise comparisons. Venn diagram depicting the number of significant metabolic features from 3 pairwise comparisons (the direction of change was ignored, *P* < 0.05, Dunn's post hoc test).

Because of the observed effects of CCB on lipid profiles, we performed a blocked Kruskal–Wallis (KW) test, using CCB treatment as the blocking factor, followed by Dunn's post hoc test for pairwise comparisons. As shown in Additional file 6, 1590 features displayed significant differences between the 3 groups, including 1395 features in PIM and 195 features in NIM (*P* < 0.05, KW test). Of these, 790 potentially matched lipids or lipid-like compounds, including lysolipids, phosphatidylcholines, carnitines, diglyceride, triglyceride, and several free fatty acids (FFAs). As depicted in Fig. 2, pairwise comparisons revealed that 1269 features displayed significant differences between NGT individuals and T2D patients, whereas 785 and 578 features showed significant differences between prediabetic individuals versus NGT individuals and T2D patients, respectively (*P* < 0.05)(Additional file 6). The low number of variables distinguishing prediabetes and T2D suggested that changes in a large fraction of the lipid features in prediabetes and T2D were shared, implying that compared with NGT, lipid profiles characterizing prediabetes and T2D are similar. Further, 117 features maintained significance after controlling the FDR by Benjamini–Hochberg multiple testing correction (FDR < 0.05)(Additional file 6).

To quantify the differential features among the 3 groups, all detected features were assessed using criteria of fold change (FC) in mass intensity ≥1.2 or ≤0.8 and variable importance of the projection (VIP) >1.0 estimated by partial least squares discriminant analysis (PLS-DA). Of the 1590 differential features, 27.80% were significantly different between T2D and NGT, representing 229 and 213 features at higher and lower levels, respectively (Additional file 6). Only 7.36% of the features differed significantly between T2D patients and prediabetic individuals, representing 58 features present in higher abundance and 59 features present in lower abundance in T2D patients compared with prediabetic individuals; 42.79% (98/229) of the lipid features detected at higher levels in T2D patients than in NGT individuals overlapped with metabolites detected at higher levels in prediabetic than in NGT individuals. However, only 18.31% (39/213) of the features detected at lower levels in T2D patients compared with NGT individuals overlapped with metabolites detected at lower level in prediabetic compared with NGT individuals. These findings suggest that the pattern of changes in the levels of a subset of metabolites reflect a continuous progression from NGT to T2D via prediabetes.

### T2D risk evaluation using random forest classifier

**Figure 3:**
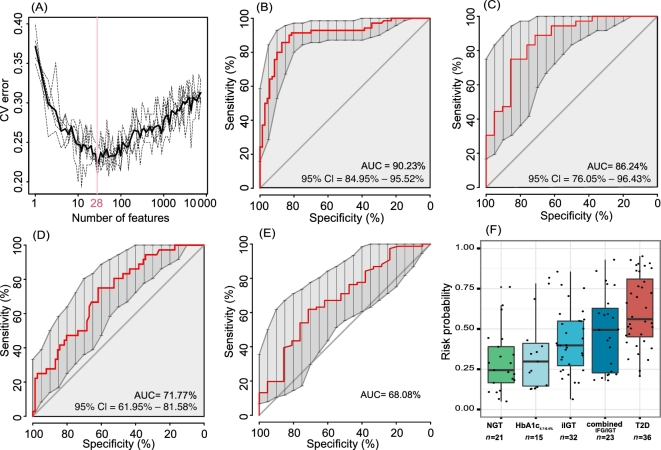
Random forest classification based on untargeted lipidomic profiling. **(A)** Distribution of 5 trials of 10-fold cross-validation error in random forest classifiers. The model was trained using relative intensity of the detected features from both positive ion mode and negative ion mode in the training set of normal glucose tolerant and type 2 diabetes patients (*n* = 70 and 70). The black solid curve indicates the average of the 5 trials (dash lines). The pink line marks the number of selected features in the optimal set. **(B)** Receiver operating characteristic curve and area under the ROC curve for the training set. **(C–E)** ROC and AUC for validation set with NGT and T2D (*n* = 21 and 36), prediabetes and T2D (*n* = 76 and 36), and NGT and prediabetes (*n* = 21 and 76), respectively. **(F)** Box-and-whisker plot presents the risk probability of developing T2D among the validated NGT (*n* = 21), subgroups of prediabetes including HbA1c_5.7–6.4%_ (*n* = 15) to iIGT (*n* = 32) to combined IFG/IGT (*n* = 23), and T2D (*n* = 36) according to the RF model.

As the qualitative and quantitative analyses revealed significant differences in the lipid levels between the 3 groups and indicated a gradual change from NGT to T2D via prediabetes, we investigated if the lipid profile could predict risk of further T2D development. To evaluate this possibility, arandom forest (RF) classifier was used. Samples for T2D and NGT groups were randomly divided into 2 sets, with 70 T2D and 70 NGT as the training set and the rest as the validation set. As illustrated in Fig. 3A, a model containing 28 features was successfully generated. The model exhibited excellent performance on the training set, with an area under the receiver operating characteristic (ROC) curve (AUC) of 90.23% (95% confidence interval [CI] = 84.95–95.52%) (Fig. 3B) and a high validated performance of AUC of 86.24% (95% CI = 76.05−96.43%) (Fig. 3C). The T2D risk prediction model was further used to evaluate the performance in relation to distinguishing prediabetes from T2D or NGT. As depicted in Fig. 3D–E, T2D or NGT was basically different from prediabetes, with an AUC of 71.77% (95% CI = 61.95−81.58%) or of 68.08 (95% CI = 54.87−81.28%), respectively. Additionally, the risk probability (RP) for each sample was estimated and indicated a gradually increasing risk from NGT to prediabetes to T2D. Interestingly, similar increasing trends of RP were observed in subgroups of the prediabetic individuals. A low risk was estimated in subjects with high HbA1c levels of 5.7–6.4%, a slightly higher riskwas estimated in subjects with isolated impaired glucose tolerance (iIGT), and a high risk was estimated in subjects with combined impaired fasting glucose (IFG) and impaired glucose tolerance (IGT) (Fig. 3F). Information on selected features, such as retention times (RTs), parent mass-to-charge ratio (*m/z*), matching compounds, and lipid categories are presented in Additional file 7, and the relative intensity levels of the features are shown in Additional file 8 (A–AB). The relative intensities of all selected features in T2D patients were significantly different from the levels in NGT subjects (Dunn's post hoc test, *P* < 0.05). In prediabetic individuals, the relative intensities of these features were intermediate between T2D and NGT, and the abundance of only a fraction of the features was significantly different when comparing prediabetes with T2D or NGT. Together, these results indicate that the lipid profile is regulated in a complex manner during development of prediabetes and T2D.

### Statistical analysis to define the correlation of T2D-related lipid compounds and diabetic parameters

**Figure 4:**
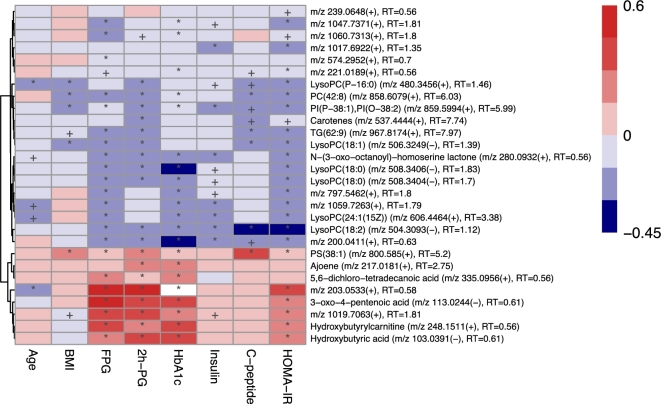
Heatmap of association between clinical parameters and 28 random forest-selected features. A hierarchically clustered heatmap of standardized regression coefficient (beta) of genera linear model analysis showing the correlations between the relative abundances of the 28 significant metabolites and the phenotypes. Red indicates positive correlations, and blue indicates negative correlations. The asterisk denotes a false discovery rate of <0.05 for each regression correlation. The cross denotes a *P-*value <0.05 and an FDR > 0.05, and the space denotes a *P-*value ≥ 0.05.

To explore the association structure between the lipidomic features and clinical diabetic parameters, we performed a general linear model regression analysis. In total, 81.38% (1294/1590) differential features were significantly correlated with at least 1 of the diabetes-related indices after adjustment for age, gender, and BMI, as well as hypertension, hyperlipidemia, smoking, and alcohol history. All RF-selected features passed the cutoff point of *P* < 0.05, and 65.69% (850/1294) met an FDR < 0.05 including 27 RF selected features except *m/z 1019.7063* (electrospray ionization [ESI]+, RT = 1.81 min) (Additional file 9). Among these 27 RF features, we observed stronger relationships between metabolites and glycemic variables (FPG, 2h-PG, and HbA1c) than beta-cell function indices (fasting insulin, C-peptide, and HOMA-IR), and weaker associations with age or BMI after adjusting for diabetes status and other confounders (Fig. 4). For instance, *m/z 203.0533* (ESI+, RT = 0.58 min) showed the highest positive correlations with glycemic variables (beta = 0.625, 0.588, and 0.466 for HbA1c, FPG, and 2h-PG, respectively), whereas a relatively weak negative correlation with age (beta = –0.197) was observed (Additional file 9). No statistically significant relationship between *m/z 203.0533* (ESI+, RT = 0.58 min) and fasting C-peptide or insulin levels was found (Additional file 9). Further, we observed an inverse relationship between lysoPC (P-16:0) at *m/z 480.3456* (ESI+, RT = 1.46 min) and the 6 diabetes-related indices. The abundance of lysoPC (P-16:0) correlated strongly with beta-cell functions (Beta = –0.334, –0.308, and –0.282 for HOMA-IR, C-peptide, and insulin, respectively) but did not correlate with age, gender, or BMI. Conversely, triglyceride (62:9) at *m/z 967.8174* (ESI+, RT = 7.97 min) was negatively correlated with both diabetes-related indexes and BMI. Among the 1590 differential features, several potential triglyceride species, including triglyceride (48:1) at *m/z 832.7403* (ESI+, RT = 8.25 min), triglyceride (50:1) at *m/z 855.7427* (ESI+, RT = 8.46 min), triglyceride (50:2) at *m/z 853.7262* (ESI+, RT = 8.35 min), and triglyceride (50:3) at *m/z 851.7108* (ESI+, RT = 8.25 min), showed high positive correlations with both BMI and C-peptide levels (*P* < 0.05) (Additional file 9).

### T2D-related lipid identification using DDA

The RF-selected features were further identified by DDA and classified by Metabolomics Standards Initiative (MSI) according to their degree of physicochemical and/or spectral similarity to available reference lipid standards or to published data [16]. The *m/z 248.1511* (ESI+, RT = 0.56 min) with a reported prominent fragment ion at *m/z* 85 was annotated as hydroxybutyrylcarnitine (Additional file 10) [17]. The highest abundance of this metabolite was detected in T2D, whereas the lowest levels were found in NGT (Additional file 8C). By comparing mass spectra of precursor ions using Progenesis QI 2.0, 2 RF-selected negative features, namely *m/z 508.3404* (RT = 1.7 min) and *m/z 508.3406* (RT = 1.83 min), with different retention times but almost identical *m/z* values, were both annotated as LysoPC (18:0). The identity of the compound was subsequently confirmed by comparison with the authentic reference standard, with the most abundant peak at *m/z 283.2641* corresponding to the acyl anion fragments of stearic acid (Additional file 11). Further, *m/z 506.3249* (ESI–, RT = 1.39 min) and *m/z 504.3093* (ESI–, RT = 1.12 min) were annotated as LysoPC (18:1) and LysoPC (18:2), with characteristic peaks at *281.2483* and *279.2326* as fragments of oleic and linoleic acid, respectively (Additional files 12 and 13). We suggest that the peak with *m/z 224* present in spectra of all 3 compounds is the product of ketene loss of the demethylated lysoPCs (Additional files 11–13) [18, 19]. The levels of the 3 LysoPC species were similar in NGT and prediabetes; however, the levels of all 3 were significantly lower in T2D patients than in NGT and prediabetic individuals (Additional file 8B, G, P, and R).

## Discussion

Prediabetes, representing a high-risk condition that precedes onset of T2D, has attracted significant attention in relation to T2D prevention and pathogenesis research. To evaluate the risk of T2D development, Morris et al. examined 70 follow-up studies where prediabetic subjects were defined by different criteria and estimated the progression rates per 1000 person-years from prediabetes to T2D. The meta-analysis demonstrated T2D incidences of 35.54/1000 for HbA1c_6.0–6.4%_, 45.46/1000 for iIGT, and 70.36/1000 for combined IFG/IGT [20]. The T2D risk of similar prediabetic subgroups in this study, HbA1c_5.7–6.4%_, iIGT, and combined IFG/IGT, was assessed using the RF algorithm, which predicted a gradually increased T2D risk from HbA1c_5.7–6.4%_ to iIGT to combined IFG/IGT. This prediction is overall consistent with the progression rates determined in the analysis of Morris et al. [20], but we also emphasize that a thorough statistical validation using longitudinal studies ofa larger cohort will be required to demonstrate the clinical utility of our model in the Han Chinese population. The low risk probability predicted for prediabetic individuals diagnosed with elevated HbA1c suggests that appropriate HbA1c criteria for Chinese prediabetic individuals should also be determined. Interestingly, Meikle's group has developed several T2D risk classification models to evaluate their performances in relation to stratifying T2D and IGT from NGT, all with FPG < 6.1 mmol/L. As reported, their combined model with common risk factors and certain plasma lipids showed a maximum AUC of 0.826 and significant gains in both a mean AUC of 0.049 (*P* < 0.001) and a net reclassification improvement of 10.5% (*P* < 0.001) compared with the model based solely on common risk factors such as BMI, gender, and HbA1c [8]. Diglyceride and triglyceride species are the 2 most frequently incorporated lipid classes in the classification models, including the diglycerides 16:0/16:0, 16:0/22:5, and 16:0/22:6, and triglyceride 14:1/16:1/18:0. As mentioned above, a significant number of lipids from these 2 classes showed significant differences between the3 clinical groups in our study, though, with few species incorporated in the risk prediction model. Our results, together with results from earlier studies, indicate that plasma lipid species might reflect some of the more subtle pathological changes during diabetic development.

In the present study, the major lipidomic alterations associated with T2D were characterized by enhanced levels of acylcarnitines and decreased levels of lysophosphatidylcholines. At MSI level 2, the abundance of hydroxybutyrylcarnitine at *m/z 248.1511* (ESI+, RT = 0.56 min) (Additional file 8C) was found to increase from NGT to prediabetes to T2D (KW test, *P* = 2.39E-9, FDR = 1.32E-5) (Additional file 6). *In vivo*, D-3-hydroxybutyrylcarnitine can be converted to D-3-hydroxybutyric acid, which is the predominant ketone body found during diabetic ketoacidosis [21–23]. Notably at MSI level 4, the abundance of hydroxybutyric acid at *m/z 103.0391* (ESI–, RT = 0.61 min) (Additional file 8J) was also higher in T2D patients than in both prediabetic and NGT individuals (KW test, *P* = 2.38E-8, FDR = 1.01E-5) (Additional file 6) in this study. These 2 features also showed high positive correlations with glycemic variables and HOMA-IR (Fig. 4, Additional file 9). Increased D-3-hydroxybutyric acid level has also been suggested as an early biomarker of insulin resistance, which may be linked to mitochondrial dysfunction and the resultant oxidative stress [21].

Both T2D and prediabetes displayed relatively higher concentrations of several short- and long-chain AcylCNs, such as L-acetylcarnitine (C2) at *m/z 203.1160n* (ESI+, RT = 0.57 min; *P* = 6.66E-04, FDR = 0.066), tetradecanoylcarnitine (C14) at *m/z 372.3115* (ESI+, RT = 0.99 min; *P* = 1.46E-03, FDR = 0.094), 2-hydroxyhexadecanoylcarnitine (C16OH) at *m/z 416.3371* (ESI+, RT = 1.02 min; *P* = 1.60E-04, FDR = 0.044), and 12-hydroxy-12-octadecanoylcarnitine (C18OH) at *m/z 444.3667* (ESI+, RT = 1.38 min; *P* = 5.70E-04, FDR = 0.066) (KW test, Additional file 6). As reported, accumulation of these incompletely oxidized lipid species, likely derived from fatty acid or amino acid metabolism, may contribute to insulin resistance [6, 24]. Increased acylcarnitine concentrations in the plasma of patients with T2D and prediabetes were also reported in the German population [25].

Lysophosphatidylcholine, an important signaling molecule and fatty acid carrier, constitutes 5–20% of total plasma phospholipids [26]. The alterations in species of LysoPC have been widely studied in relation to diabetes and obesity. Significantly lower levels of several LysoPC species in patients with IGT and T2D, including LysoPC (18:2), LysoPC (18:1), LysoPC (18:0), and LysoPC (17:0), were reported in a large cross-sectional study [27]. In addition, LysoPC (18:2) and glycine were selected and validated as strong baseline predictors for the risk of developing IGT and/or T2D in a prospective analysis [27]. Finally, shotgun lipidomics and data mining approaches revealed multiple independent associations between plasma lipidomic parameters and insulin sensitivity indices, including a negative correlation between LysoPC (22:5) and HOMA-IR [28]. It has been reported that LysoPC species could enhance glucose-dependent insulin secretion via G-protein-coupled receptor G119 both *in vivo* and *in vitro* [29].

Comparing NGT and prediabetic individuals, we observed a strongly significant reduction of lysophosphatidylcholine species in the Chinese T2D patients including lysophosphatidylcholine (18:0) at *m/z 508.3406* (ESI–, RT = 1.83 min; *P* = 3.01E–7, FDR = 5.55E–05) and *m/z 508.3404* (ESI–, RT = 1.7 min; *P* = 2.93E–05, FDR = 1.88E–03), lysophosphatidylcholine (18:1) at *m/z 506.3249* (ESI–, RT = 1.39 min; *P* = 7.95E–06, FDR = 6.67E–04), and lysophosphatidylcholine (18:2) at *m/z 504.3093* (ESI–, RT = 1.12 min; *P* = 6.28E–06, FDR = 3.41E–03), which were selected by the T2D prediction model (KW test, Additional file 6). Also, lysophosphatidylcholine (22:5) at *m/z 569.3440n* (ESI+, RT = 1.01 min) showed a slight decrease in T2D and prediabetes (*P* = 0.027, FDR = 0.310), while no significant associations with HOMA-IR or other diabetes-related indices were observed. The differences suggest the presence of distinct lipid profiles characterizing T2D populations in China and Germany.

Some T2D-associated features are also tightly correlated with obesity. Compared with lean control, Melissa et al. reported a reduction of several LysoPC species in both obese T2D and obese non-T2D subjects, while no differences were observed between the 2 obese groups [30]. In this study, 641 significant features selected by the KW test displayed significant associations with BMI, by adjusting age, gender, diabetes status, hypertension history, hyperlipidemia history, smoking history, and alcohol history, with 107 of them solely correlating with BMI (*P* < 0.05, Additional file 9). Notably, 7 of the RF-selected features showed additional significant associations with BMI, including the positively related PS (38:1) at *m/z 800.5850* (ESI+, RT = 5.20 min) and negatively associated LysoPC (18:1) at *m/z 506.3249* (ESI–, RT = 1.39 min), TG (62:9) at *m/z* 967.8174 (ESI+, RT = 7.97 min), and LysoPC (18:2) at *m/z 504.3093* (ESI–, RT = 1.12 min) (Fig. 4, Additional file 9). Lower levels of LysoPC (18:1) have also been observed in obese subjects [31, 32]. In addition, a recent review listing metabolic biomarkers of obesity and T2D noted a large number of shared lipid biomarkers between the 2 disorders [33]. Additionally, 5 of the RF-selected features showed additional significant negative associations with age, including *m/z 203.0533* (ESI+, RT = 0.58 min) and LysoPC (18:2) at *m/z 504.3093* (ESI–, RT = 1.12 min) (Fig. 4, Additional file 9). This indicates that there are common alterations in lipid metabolism associated with T2D, obesity, and aging, supporting the idea that greater BMI and aging are the 2 main risk factors for developing T2D [34–36].

We observed that CCB treatment (*n* = 61) for hypertension showed slight effects on plasma lipid profiles (*P* = 0.0324 and FDR = 0.0825) by PERMANOVA (Additional file 5), which was consistent with an earlier report [37]. No significant effects were found in relation to other medications, probably because of smaller sample sizes or limited impacts (Additional file 5). Although further validation is required to confirm and validate drug effects, we suggest that the medications as possible interfering factors on lipid metabolism [37, 38] should be carefully considered in lipidomics-based investigations. Hence, we used strict statistical analyses to control for possible effects of treatment on T2D-associated plasma lipids.

In summary, by using LC-MS/MS-based untargeted lipidomics analysis, our study is the first large-scale study to explore the alterations in the plasma lipid patterns of individuals with NGT, prediabetes, and T2D from East China. We describe a large number of plasma lipids providing a broad coverage of major lipid categories. We identify thousands of plasma lipids exhibiting remarkable difference in abundance between the 3 diagnostic groups, with a large proportion displaying similar trends in prediabetes and T2D. Additionally, we describe stratification of predicted diabetes risk between subgroups of prediabetes based on 28 selected plasma lipids. Several of the diabetes-related candidates have not previously been reported. Together, this study provides a better biological understanding of the insidious progression to diabetes from a lipid perspective. More comprehensive studies combining genomics, metabolomics, proteomics, and metagenomics should be conducted to describe the detailed variations among the prediabetes subgroups and to support precise prevention and intervention steps for T2D.

## Methods

### Participant recruitment, sampling, and grouping

Four-hundred and thirty-three participants submitted written informed consent forms and were enrolled in the study from the community health service centers of Suzhou Center for Disease Prevention and Control (CDC). All participants underwent a 2-step enrollment process. At the first visit, all participants were subjected to physical examinations including height, weight, blood pressure, and waist and hip circumference and completed a face-to-face questionnaire on demographics, medication history, family health history, and other lifestyle factors via well-trained local staff. The study enrolled only those participants who met the following criteria based on questionnaire: 1) age 40 or older; 2) free of cardiovascular disease, severe renal disease, cancer, type 1 or monogenic diabetes, and other autoimmune diseases, as determined by self-reporting; and 3) no antibiotic use during the past 2 months. Approximately 81.7% of the participants in the cohort (354 out of 433) meeting the above criteria were admitted to a blood screening test for diabetes according to the 2011 WHO criteria [39]. The qualified participants without a self-reported history of T2D were given a 2-hour 75-g oral glucose tolerance test. Participants with fasting or postprandial blood glucose levels above the diagnostic cut-off point were asked to repeat the test on the next day. Blood medical tests were performed by a Nanjing Kingmed Center for Clinical Laboratory, which included FPG, insulin, C-peptide, HbA1c, leptin, adiponectin, and blood lipid levels in addition to routine blood tests. Fasting plasma was prepared within 1 hour after blood withdrawal by centrifuged at 1600 g for 15 minutes. The upper layers were carefully collected to avoid disturbing the buffy coat cells. The isolated plasma samples were stored at –80°C and transported on dry ice to BGI-Shenzhen.

Finally, a total of 293 subjects were divided into the 3 diagnostic groups, namely the normal glucose tolerance group (*n* = 98), the prediabetes group (*n* = 81), and the T2D group (*n* = 114; including 77 newly diagnosed and 37 self-reported patients). The prediabetes samples were further classified into 4 subgroups: (i) raised HbA1c _5.7–6.4%_ (defined by the WHO–HbA1c criteria only; *n* = 15); (ii) isolated IFG (defined by an FPG level of 6.1–7.0 mmol/l and a normal 2h-PG level; *n* = 7); (iii) isolated IGT (defined by a normal FPG level and a 2h-PG level of 7.8–11.0 mmol/l; *n* = 35); and (iv) combined IFG/IGT (defined by an FPG of 6.1–7.0 mmol/l and a 2h-PG level of 7.8–11.0 mmol/l; *n* = 24). The study was approved by the Institutional Review Board of BGI-Shenzhen and the ethical review committee of Suzhou CDC.

### Lipidome data processing and analysis (Lipidomics)

#### Lipid preparation and extraction

The collected plasma samples were thawed on ice, and lipids were extracted with isopropanol (IPA) using a previously described method [40]. Briefly, 40 μL of plasma was extracted with 120 μL of precooled IPA, vortexed for 1 minute, and incubated at room temperature for 10 minutes; the extraction mixture was then stored overnight at –20°C. After centrifugation at 4000 g for 20 minutes, the supernatants were transferred into new 96-well plates and diluted to 1:10 with IPA/acetonitrile (ACN)/H_2_O (2:1:1, v:v:v). The samples were stored at –80°C prior to the LC-MS analysis. In addition, pooled plasma samples were also prepared by combining 10 μL of each extraction mixture.

#### Ultra-performance liquid chromatography–mass spectrometry method for lipidomics

Samples were analyzed with an ACQUITY UPLC (Waters, Manchester, NH, USA) connected to a XEVO-G2XS quadrupole time-of-flight (QTOF) mass spectrometer (Waters) with ESI. The lipids were separated using an Acquity UPLC charged surface hybrid C18 column (2.1 × 100 mm, 1.7 μm, Waters) with a gradient mobile phase comprised of 10 mM ammonium formate with 0.1% formic acid in acetonitrile/water (A, 60:40, *v/v*) and 10 mM ammonium formate with 0.1% formic acid in isopropanol/acetonitrile (B, 90:10, *v/v*). Before the large-scale study, pilot experiments including 10-minute, 15-minute, and 20-minute elution periods were conducted to evaluate the potential effects of mobile phase composition and flow rate on lipids retention time. Both abundant lipid precursors ions and fragments were separated in the same order with similar peak shapes and ion intensities in PIM (Additional file 14A–C, Additional file 15A–C). Furthermore, the mixed QC samples with the 10-minute elution period also showed similar base peak intensities of precursors and fragments with the test sample (Additional file 14D, Additional file 15D). Considering the large sample size of this study, we used an accelerated elution profile of 10 minutes, described in the following sections. The mobile phase was delivered at a flow rate of 0.4 mL/min. The column was initially eluted with 40% B, followed by a linear gradient to 43% B over 2 minutes, and then the percentage of B was increased to 50% within 0.1 minutes. Over the next 3.9 minutes, the gradient was further ramped to 54% B, and the amount of B was then increased to 70% in 0.1 minutes. In the final part of the gradient, the amount of B was increased to 99% over 1.9 minutes. Finally, solution B was returned to 40% in 0.1 minutes, and the column was equilibrated for 1.9 minutes before the next injection. The injection volume was 10 μL. Lipids were detected with a XEVO-G2XS QTOF mass spectrometer in positive and negative mode, which was operated in MS^E^ mode from *m/z 50–2000*, with an acquisition time of 1 second per scan. The source temperature was set at 120°C. The desolvation temperature and gas flow were 600°C and 800 L/h, respectively, and nitrogen was used as the flow gas. The capillary and cone voltages were 2.0 kV(+)/1.5 kV(–), and 30 V, respectively. Leucine encephalin (molecular weight = 555.62; 200 pg/μL in 1:1 acetonitrile:H_2_O) was used as a lock mass for accurate mass measurements, and 0.5-mM sodium formate solution was used for calibration. The samples were randomly ordered, and 10 QC samples were initially injected to condition the column. One QC sample was injected and analyzed every 10 samples to investigate the repeatability of the data [41].

#### Acquisition of the high-quality non-targeted metabolic profile and metabolite identification

The raw tandem mass spectrometry datasets were generated on the Waters XEVO-G2XS QTOF instrument and processed using commercial software Progenesis QI 2.0 (Nonlinear Dynamics, Newcastle, UK), consisting of raw data import, selection of possible adducts, peak set alignment, peak detection, deconvolution, dataset filtering, noise reduction, compound identifi-cation, and normalization with sum method. The analysis parameters used were as follows: (i) possible adducts of [M+H]^+^, [M+H–H2O]^+^, [M+Na]^+^, and [M+K]^+^ for ESI+ and [M–H]^–^ for ESI–; (ii) the retention time of 0.5–9 minutes; (iii) the peak width of 1–30s; (iv) 10-ppm mass tolerance for the precursors; and (iv) 10-ppm fragment mass tolerance for theoretical fragmentation searching to improve the confidence in compound identification. The normalized peak data were further preprocessed by an in-house software metaX [15]. The k-nearest neighbor algorithm to further improve the data quality. PCA was performed for outlier detection and batch effects evaluation using the pre-processed dataset. Quality controlrobust spline correction (QC-RSC) was fitted to the QC data with respect to the order of injection to minimize signal intensity drift over time. In addition, the relative standard deviations of the metabolic features were calculated across all QC samples, and those >30% were then removed. The high-resolution LC-MS/MS features were identified using Progenesis QI 2.0 by searching in the public databases including Human Metabolome Database (HMDB) [42], LIPID MAPS Structure Database (LMSD) [43], and LipidBlast [44] with the mentioned parameters. To obtain reliable identification of the high-quality features, matching lipids were filtered by defined retention time ranges in terms of the application note of the CSHC_18_ UPLC System provided by Waters Corporation [45]. RTs were 0.5–4 minutes for lysophospolipids including lysophosphatidylcholine, lysophosphatidylethanolamine, lysophosphatidylglycerol, lysophosphatidylserine, lysophosphatidic acid, and lysophosphatidylinositol species, 3–8.1 minutes for sphingolipids, including sphingomyelin, ceramide and lactosylceramide, glucosylceramide, and galactosylceramide species, 4–7.8 minutes for phosphatidylcholine, phosphatidylethanolamine, phosphatidylglycerol, phosphatidylserine, phosphatidic acid, and phosphatidylinositol species, 7.8–9.5 minutes for diglyceride, triglyceride, and cholesteryl ester species in PIM (Additional file 14D, Additional file 15D), 0.5–4 minutes for lysophospolipids and FFA species, and 4–9 minutes for phospholipids, including phosphatidylcholine, phosphatidylethanolamine, phosphatidylglycerol, phosphatidylserine, phosphatidic acid, and phosphatidylinositol, and sphingolipid species in NIM. The metabolites that could be matched to LMSD, LipidBlast, or the aliphatic compounds of HMDB at the molecular framework level were considered lipids and lipid-like features. DDA, which covered the desired mass scan range of interest, was performed to further aid in identifying the metabolites. Pure authentic standard of lysophosphatidylcholine (18:0; product code: 855775P from Avanti Polar Lipids Inc, Alabaster, AL, USA) was used to validate the lipids by comparing their MS/MS spectra and retention time on the Waters XEVO-G2XS QTOF instrument. The reported features were classified into MSI levels according to the reported guidelines [16].

## Data analysis

### Univariate analysis: KW testing and fold-change analysis

Blocked KW tests were conducted to detect differences in metabolite concentrations among the 3 diagnostic groups after controlling for the potential confounding effects of CCBs. The analysis was performed using the tools implemented in the COIN software package (coin 1.1–2 in R 3.2.5). The *P* value was adjusted for multiple tests using an FDR (Benjamini–Hochberg). Dunn's post hoc tests followed by pairwise comparisons were performed: an equals sign indicates no significant difference, and a greater than symbol indicates *P-*values < 0.05. Fold-changes were calculated by comparing the mean concentrations of each feature between groups.

To improve the performance of the subsequent multivariate statistical analyses, all features were normalized to the range of [0, 1] to stabilize the variance using a modified range-scaling method with the following formula [46]:

\begin{equation*}
\tilde{x}_{ij} = \frac{x_{ij} - {x_{{i_{\min}}}}}{{\left( {{x_{{i_{\max}} - }}{x_{{i_{\min}}}}} \right)}},
\end{equation*}

where ${\tilde{x}_{ij}}$ indicates the scaled value for *i*th variable (compound) in the *j*th sample (which is valued by *x_ij_*,) and ${x_{{i_{\max}}}}$ and ${x_{{i_{\min}}}}$ represent the maximum and minimum values for the *i*th variable among the samples, respectively.

### PERMANOVA for the influence of clinical and lifestyle factors

PERMANOVA was performed on the normalized lipid metabolite profiles and phenotypes with Bray–Curtis distance (adonis function, vegan package in R 3.2.5). The number of permutations was 9999. The *P-*value was corrected for multiple tests using an FDR (Benjamini–Hochberg) cut-off of 0.05.

### PLS-DA analysis

Supervised PLS-DA was conducted through metaX [15] to discriminate the different variables between groups. The VIP value was calculated. A VIP cut-off value of 1.0 was used to select important features.

### Random forest (ROC/AUC) analysis

The RF classifier (randomForest 4.6-12 in R 3.2.5) was trained on 140 randomly selected subjects (70 NGT and 70 T2D) from the 273 samples and then tested on the remaining subjects. All of the features were supplied to the classifier. The analysis was conducted with 5 repetitions of the 10-fold cross-validation, using cross-validation error curves to selected features as described by Feng et al. [47]. The risk probability of T2D for each subject was computed by the selected features, and a ROC curve was drawn, for which the AUC was calculated (pROC1.8 in R 3.2.5). The selection frequencies of features were listed to measure the importance of the variables, with a higher frequency indicating the greater importance of a given metabolite for classifying T2D and NGT. The RF model was further tested on the validation sets.

### General linear model regression analysis

GLM regression analysis was conducted to investigate the associations between metabolites and multiple clinical phenotypes. In a model applied with metabolites and diabetes-related indexes including FPG, 2h-PG, HbA1c, fasting insulin, C-peptide, and HOMA-IR, the confounding factors including age, BMI, gender, and CCB use were adjusted. The model applied with metabolites and age was adjusted for diabetes status, BMI, gender, and CCB use. The model applied with metabolites and BMI was adjusted for diabetes status, age, gender, and CCB use. For each feature, the standardized regression coefficient (beta) and 2-tailed *P-*value for the coefficient was calculated (glm in R 3.2.5). A *P*-value of less than 0.05 was regarded as significant.

### Additional files

Additional file 1: phenotypic and clinical information for 293 enrolled subjects.

Additional file 2: batch numbers and run orders for biological samples and quality controls.

Additional file 3: detailed list of total detected plasma features.

Additional file 4: principal component analysis of plasma lipid profiling from biological samples and quality controls. Principal component analysis was performed on all samples to identify run outliers and check for possible batch effects in both positive (A) and negative modes (B). The colors represent the different sample classes: green for normal glucose tolerant, blue for prediabetes, red for type 2 diabetes, orange for quality controls, and black for outliers.

Additional file 5: permutational multivariate analysis of variance of the influence of clinical records or life habits on lipid profile.

Additional file 6: detailed list of significant features among 3 groups.

Additional file 7: detailed list of 28 metabolic features selected by random forest classifiers.

Additional file 8: box plot displays the relative intensity levels of 28 selected diabetic-related features in normal glucose tolerance, prediabetes, and type 2 diabetes. The features are presented in order of decreasing importance according to the selection frequencies in a random forest model. One asterisk denotes *P <* 0.05, 2 denote *P <* 0.01, and 3 denote *P <* 0.001 (Dunn's post hoc test).

Additional file 9: a generalized linear model analysis on 1590 significant features and clinical phenotypes.

Additional file 10: tandem mass spectrometry spectra of *m/z 248.1511* (ESI+, RT = 0.56 min) and its inferred chemical structure. Product ion spectra obtained from MS/MS of *m/z 248.1511* [M+H]^+^ in the positive ion mode. Each arrow indicates a possible site of fragmentation, including a product ion at *m/z 85*, which could be produced by all acylcarnitine butyl esters, and a product at *m/z 103*, which has been reported as aliphatic hydroxyl group-containing fragment to produce the ion at *m/z 85*. These spectra indicate that m/z *248.1511* corresponds to hydroxybutyrylcarnitine +H. ESI+: positive electrospray ionization; RT: retention times.

Additional file 11: extracted-ion chromatogram and tandem mass spectrometry spectra of *m/z 508.34* (RT = 1.70 minutes and RT = 1.83 min) in quality control samples and lysophosphatidylcholine (18:0) standard reference. **(A)** and **(C)** display the extracted-ion chromatogram of m/z *508.34* [M–CH3]^–^ in quality control sample and lysophosphatidylcholine (18:0) standard acquired in the negative ion mode. **(B)** and **(D)** exhibit the tandem mass spectrometry spectra of *m/z 508.34* [M–CH3]^–^ in the QC sample and lysophosphatidylcholine (18:0) standard. Each arrow in the MS/MS spectrum of lysophosphatidylcholine (18:0) indicates a reported site of fragmentation, with the most intense product ion at *m/z 283.2639* corresponding to fatty acid 18:0. The other less abundant product ion at *m/z 168* corresponds to N-dimethylaminoethylphosphate anion, and ions at *m/z 224* and *m/z 242* to the products of ketene losses from demethylated lysophosphatidylcholine (18:0). These spectra confirmed the identification of *m/z 508.34* (RT = 1.70 min, RT = 1.83 min) as lysophosphatidylcholine (18:0)–CH3.

Additional file 12: tandem mass spectrometry spectra of *m/z 506.3249* (ESI–, RT = 1.38 min) and its inferred chemical structure. Product ion spectra obtained from tandem mass spectrometry of *m/z 506.3249* [M–CH3]^–^ in the negative ion mode. Each arrow indicates a possible site of fragmentation, with the most intense product ion at *m/z 281.2483* corresponding to 18:1 fatty acid. The spectra indicate that m/z *506.3249* corresponds to lysophosphatidylcholine (18:1)–CH3.

Additional file 13: tandem mass spectrometry spectra of *m/z 504.3093* (ESI–, RT = 1.12 min) and its inferred chemical structure. Product ion spectra obtained from tandem mass spectrometry of *m/z 504.3093* [M–CH3]^–^ in the negative ion mode. Each arrow indicates a possible site of fragmentation, with the most intense product ion at *m/z 279.2326* corresponding to 18:2 fatty acid. These spectra indicate that m/z *504.3093* corresponds to lysophosphatidylcholine (18:2)–CH3.

Additional file 14: the base peak intensity of precursors (MS1) in positive ion mode across the whole mass range. **(A–C)** indicate test plasma samples with liquid chromatography gradients of 20 minutes, 15 minutes, and 10 minutes. **(D)** indicates a quality control sample from this study with a retention time of 10 minutes. As shown in (D), the common high abundant precursor ions may represent characteristic patterns corresponding to certain lipid species extracted from human plasma. For instance, the ions at *m/z 496.35* (RT = 1.31 min), *m/z 524.38* (RT = 1.81 min), and *m/z 758.58* (RT = 6.30 min) may be suggested as [lysophosphatidylcholine (16:0)+H]^+^, [lysophosphatidylcholine (18:0)+H]^+^, and [lysophosphatidylcholine (16:0/18:2)+H]^+^, respectively [50], the ions at *m/z 780.56* (RT = 5.15 min) and *m/z 782.57* (RT = 5.33 min) as [lysophosphatidylcholine (36:5)+H]^+^ and [lysophosphatidylcholine (34:1)+Na]^+^, and ions at *m/z 369.35* (RT = 8.47 min) as [cholesterol–H2O+H]^+^, a cholestadiene cation generated from cholesteryl esters [51]. The abundant ion at *m/z 577.52* (RT = 8.42 min) has been reported to indicate the sodiated 18:2 fatty acyl group containing a keto moiety formed by triglyceride species [52].

Additional file 15: the base peak intensity of fragments (MS2) in positive ion mode across the whole mass range. **(A–C)** indicate test plasma samples with liquid chromatography gradients of 20 minutes, 15 minutes, and 10 minutes. **(D)** indicates a quality control sample from this study with a retention time of 10 minutes. The most abundant fragment ions at *m/z 184* have been reported as protonated-phosphocholine moieties that are diagnostic for the phosphatidylcholine head group class [53, 54].

## Abbreviations

2h-PG: 2-hour postprandial glucose; AUC: area under the curve; BCAA: branched-chain amino acid; BMI: body mass index; CCB: calcium channel blocker; CI: confidence interval; CV: coefficient of variation; DDA: data-dependent acquisition; DIA: data-independent acquisition; ESI: electrospray ionization; FDR: false discovery rate; FPG: fasting plasma glucose; FFA: free fatty acid; HbA1c: glycated hemoglobin; HMDB: Human Metabolome Database; HOMA-IR: insulin resistance index; IFG: impaired fasting glucose; IGT: impaired glucose tolerance; iIGT: isolated impaired glucose tolerance; KW test: Kruskal–Wallis test; LC-MS/MS: liquid chromatography-tandem mass spectrometry; LDL: low-density lipoprotein; LMSD: LIPID MAPS Structure Database; MS: mass spectrometry; MSI: Metabolomics Standards Initiative; NGT: normal glucose tolerant; NIM: negative ion mode; PCA: principal component analysis; PERMANOVA: permutational multivariate analysis of variance; PIM: positive ion mode; PLS-DA: partial least squares discriminant analysis; QC: quality control; QTOF: quadrupole time-of-flight; RP: risk probability; RT: retention time; RF: random forest; ROC: receiver operating characteristic; SBP: systolic blood pressure; T2D: type 2 diabetes mellitus; TC: total cholesterol; UPLC-MS: ultra-performance liquid chromatography–mass spectrometry; VIP: variable importance of the projection; WHO: World Health Organization.

## Supplementary Material

GIGA-D-16-00114_Original-Submission.pdf

GIGA-D-16-00114_Revision-1.pdf

GIGA-D-16-00114_Revision-2.pdf

GIGA-D-16-00114_Revision-3.pdf

Response-to-reviewers_Original-Submission.pdf

Response-to-Reviewers_Revision-1.pdf

Reviewer-1-Report-(Original-Submission).pdf

Reviewer-1-Report-(Revision-1).pdf

Reviewer-2-Report-(Original-Submission).pdf

Reviewer-3-Report-(Original-Submission).pdf

Additional file 1: phenotypic and clinical information for 293 enrolled subjects.

Additional file 2: batch numbers and run orders for biological samples and quality controls.

Additional file 3: detailed list of total detected plasma features.

Additional file 4: principal component analysis of plasma lipid profiling from biological samples and quality controls. Principal component analysis was performed on all samples to identify run outliers and check for possible batch effects in both positive (A) and negative modes (B). The colors represent the different sample classes: green for normal glucose tolerant, blue for prediabetes, red for type 2 diabetes, orange for quality controls, and black for outliers.

Additional file 5: permutational multivariate analysis of variance of the influence of clinical records or life habits on lipid profile.

Additional file 6: detailed list of significant features among 3 groups.

Additional file 7: detailed list of 28 metabolic features selected by random forest classifiers.

Additional file 8: box plot displays the relative intensity levels of 28 selected diabetic-related features in normal glucose tolerance, prediabetes, and type 2 diabetes. The features are presented in order of decreasing importance according to the selection frequencies in a random forest model. One asterisk denotes *P <* 0.05, 2 denote *P <* 0.01, and 3 denote *P <* 0.001 (Dunn's post hoc test).

Additional file 9: a generalized linear model analysis on 1590 significant features and clinical phenotypes.

Additional file 10: tandem mass spectrometry spectra of *m/z 248.1511* (ESI+, RT = 0.56 min) and its inferred chemical structure. Product ion spectra obtained from MS/MS of *m/z 248.1511* [M+H]^+^ in the positive ion mode. Each arrow indicates a possible site of fragmentation, including a product ion at *m/z 85*, which could be produced by all acylcarnitine butyl esters, and a product at *m/z 103*, which has been reported as aliphatic hydroxyl group-containing fragment to produce the ion at *m/z 85*. These spectra indicate that m/z *248.1511* corresponds to hydroxybutyrylcarnitine +H. ESI+: positive electrospray ionization; RT: retention times.

Additional file 11: extracted-ion chromatogram and tandem mass spectrometry spectra of *m/z 508.34* (RT = 1.70 minutes and RT = 1.83 min) in quality control samples and lysophosphatidylcholine (18:0) standard reference. **(A)** and **(C)** display the extracted-ion chromatogram of m/z *508.34* [M–CH3]^–^ in quality control sample and lysophosphatidylcholine (18:0) standard acquired in the negative ion mode. **(B)** and **(D)** exhibit the tandem mass spectrometry spectra of *m/z 508.34* [M–CH3]^–^ in the QC sample and lysophosphatidylcholine (18:0) standard. Each arrow in the MS/MS spectrum of lysophosphatidylcholine (18:0) indicates a reported site of fragmentation, with the most intense product ion at *m/z 283.2639* corresponding to fatty acid 18:0. The other less abundant product ion at *m/z 168* corresponds to N-dimethylaminoethylphosphate anion, and ions at *m/z 224* and *m/z 242* to the products of ketene losses from demethylated lysophosphatidylcholine (18:0). These spectra confirmed the identification of *m/z 508.34* (RT = 1.70 min, RT = 1.83 min) as lysophosphatidylcholine (18:0)–CH3.

Additional file 12: tandem mass spectrometry spectra of *m/z 506.3249* (ESI–, RT = 1.38 min) and its inferred chemical structure. Product ion spectra obtained from tandem mass spectrometry of *m/z 506.3249* [M–CH3]^–^ in the negative ion mode. Each arrow indicates a possible site of fragmentation, with the most intense product ion at *m/z 281.2483* corresponding to 18:1 fatty acid. The spectra indicate that m/z *506.3249* corresponds to lysophosphatidylcholine (18:1)–CH3.

Additional file 13: tandem mass spectrometry spectra of *m/z 504.3093* (ESI–, RT = 1.12 min) and its inferred chemical structure. Product ion spectra obtained from tandem mass spectrometry of *m/z 504.3093* [M–CH3]^–^ in the negative ion mode. Each arrow indicates a possible site of fragmentation, with the most intense product ion at *m/z 279.2326* corresponding to 18:2 fatty acid. These spectra indicate that m/z *504.3093* corresponds to lysophosphatidylcholine (18:2)–CH3.

Additional file 14: the base peak intensity of precursors (MS1) in positive ion mode across the whole mass range. **(A–C)** indicate test plasma samples with liquid chromatography gradients of 20 minutes, 15 minutes, and 10 minutes. **(D)** indicates a quality control sample from this study with a retention time of 10 minutes. As shown in (D), the common high abundant precursor ions may represent characteristic patterns corresponding to certain lipid species extracted from human plasma. For instance, the ions at *m/z 496.35* (RT = 1.31 min), *m/z 524.38* (RT = 1.81 min), and *m/z 758.58* (RT = 6.30 min) may be suggested as [lysophosphatidylcholine (16:0)+H]^+^, [lysophosphatidylcholine (18:0)+H]^+^, and [lysophosphatidylcholine (16:0/18:2)+H]^+^, respectively [50], the ions at *m/z 780.56* (RT = 5.15 min) and *m/z 782.57* (RT = 5.33 min) as [lysophosphatidylcholine (36:5)+H]^+^ and [lysophosphatidylcholine (34:1)+Na]^+^, and ions at *m/z 369.35* (RT = 8.47 min) as [cholesterol–H2O+H]^+^, a cholestadiene cation generated from cholesteryl esters [51]. The abundant ion at *m/z 577.52* (RT = 8.42 min) has been reported to indicate the sodiated 18:2 fatty acyl group containing a keto moiety formed by triglyceride species [52].

Additional file 15: the base peak intensity of fragments (MS2) in positive ion mode across the whole mass range. **(A–C)** indicate test plasma samples with liquid chromatography gradients of 20 minutes, 15 minutes, and 10 minutes. **(D)** indicates a quality control sample from this study with a retention time of 10 minutes. The most abundant fragment ions at *m/z 184* have been reported as protonated-phosphocholine moieties that are diagnostic for the phosphatidylcholine head group class [53, 54].

## Availability of supporting data and materials

The raw mass spectrometry datasets have been deposited in the MetaboLights open access data repository under accession number MTBLS352. The commercial software Progenesis QI 2.0 (Nonlinear Dynamics, Newcastle, UK [48]) was purchased from Waters Corporation. The in-house software metaX was available for Bioconductor [15]. Supplemental data and custom scripts are available in the *GigaScience* database, GigaDB [49].
