# Supplementary material for: Lipidomic profiling reveals distinct differences in plasma lipid composition in healthy, prediabetic, and type 2 diabetic individuals
Source: Gigascience. 2017 May 15;6(7):1–12. doi: 10.1093/gigascience/gix036 (PMC5502363; doi:10.1093/gigascience/gix036)
Supplement: GIGA-D-16-00114_Original-Submission.pdf [file gix036_GIGA-D-16-00114_Original-Submission.pdf]

Manuscript

Click here to download Manuscript main MS. Lipidomic profiling reveals progressive changes of plasma lipids from normal to

Click here to view linked References

Lipidomic profiling reveals progressive changes of plasma lipids from normal to type 2 diabetes

Abstract

Background: Dyslipidemia was found a tight association with type 2 diabetes (T2D), whereas how to globally monitor the lipid responses to the T2D progression is remained to be uncovered, especially in East Asia population.

Results: Liquid chromatography coupled with tandem mass spectrometry (LC MS/MS) was employed for globally detecting lipidomes in the fasting plasma of 293 individuals, including 114 T2D patients, 81 prediabetes and 98 individuals with normal glucose tolerance (NGT). Both analyses of qualification and quantification revealed that the lipid features in T2D were relatively close to that in prediabetes, whereas were significantly different from that in NGT. On the basis of the lipidomic data, a random forest (RF) classifier was built to assess the T2D risk during diabetic progression from NGT or prediabetes. The model with 28 lipid features generated from RF could effectively discriminate T2D from NGT or prediabetes. The individual lipid features were further evaluated their correlations with clinical parameters for diabetes using Spearman's correlation and regression analysis. Most selected lipid features were found in the significant correlations with diabetes, typically like hydroxybutyrylcarnitine with positive correlation, and glycerophosphocholines such as LysoPC (17:0), LysoPC (17:1) and PC (17:2(9Z,12Z)/0:0) with negative correlation.

Conclusions: The impaired plasma lipidome in T2D and prediabetes was first confirmed in Chinese population. The lipid features are not only simply as the biomarkers that differ T2D and NGT, but are also as the references to evaluate the diabetic progression.

Keywords: Lipidomics, Type 2 diabetes, Prediabetes, Plasma

Background

Type 2 diabetes mellitus (T2D) is a progressive and complex disease that is tightly associated with heterogeneous metabolic disorders, particularly in glucose and lipid metabolism[1]. Recently the two documents of national epidemiology revealed that diabetes was a major health problem in China, appearing the largest population of diabetic patients in the world, not only huge number of diabetic patients but also the disease incidence rate. The total prevalence of adult diabetes in China was estimated approximate 9.7% in 2007 using the 1999 World Health Organization (WHO) criteria, moreover that was accessed about 11.6% in 2010 according to the American Diabetes Association (ADA) criteria[2,3]. For the sake of monitoring T2D development, the

concept of prediabetes was introduced, in which prediabetes represents a person whose blood sugar level is higher than normal, but not high enough for a diagnosis of diabetes. The uniform criteria to define prediabetes in clinical practice are still in argument. For instance, both WHO and ADA have proposed 2-hour postprandial glucose (2h-PG) of 7.8–11.0 mmol/L as the cut-off value to define prediabetes, whereas that have recommended the level of fasting plasma glucose (FPG) as another criterion with different thresholds of 6.1–6.9 mmol/L from WHO and 5.6-6.9 mmol/L from ADA, respectively[4,5]. Also, hemoglobin A1c (HbA1c) at level of 5.7% to 6.4% is a prediabetes criterion endorsed by ADA. According to the ADA criteria, the prediabetes prevalence in Chinese adults was 50.1% in 2010, approximately 38.2% of the prediabetic individuals only over the HbA1c threshold[3]. Obviously, diabetes is emerging a serious challenge to public health in China. Pursing the pathologic mechanisms and developing the medical treatments are hence becoming an urgent task in the field of diabetes study.

It is well accepted that T2D is a typical disease of metabolic disorder accompanying with activation/inactivation of metabolic pathways or augment/attenuation of metabolite concentrations. Metabolomics has arisen as a power tool to globally survey the diabetes-related metabolites in recent years[6–10]. Wang et al assessed the metabolite profiles from over 2000 normal glycemic individuals using liquid chromatography tandem mass spectrometry (LC-MS/MS) to aim the predictive indicators of diabetic development. Five branched-chain and aromatic amino acids, isoleucine, leucine, valine, tyrosine, and phenylalanine, were found highly-significant associations with diabetes, and a combination of three amino acids with five-fold higher risk for individuals were predicted future diabetes[6]. Although impaired glucose utilization through insulin resistance and β-cell defect is involved in T2D, dysregulation of fatty acids (FFA) is generally observed in these patients as well. In concert with the elevated amino acids, acylcarnitine (AcylCN) species likely derived from amino acid oxidation were concomitant increases in the T2D plasma. Arslanian's group demonstrated that plasma long-chain AcylCN species and short AcylCN intermediate were increased in the T2D adults, consequent to an augment in FFA flux into the mitochondria and in oxidation defect[7]. Furthermore, Meikle et al made a cohort study of lipidomics analysis, including 117 T2D, 64 prediabetes and 170 normal glucose tolerant (NGT) participants, and revealed that over hundred individual lipid species, such as sphingolipids, phospholipids, glycerolipids and cholesterol esters, were positively associated with T2D and prediabetes[8]. The lipidomic changes in response to diabetic development in tissues and plasma thus really pave a new avenue to deepen the pathological mechanisms and to explore the biomarkers for T2D.

As an emerging approach, study on the T2D relevant lipodomics has remained a big room to be filled. In technique side, how to acquire high quality MS/MS signals corresponding to lipid molecules is an urgent demand for both qualification and quantification

of lipids. Current analysis strategies for collecting lipidomic data are based upon the mode of data-dependent acquisition (DDA) in most laboratories, in which the parameters of mass spectrometry, such as number of precursor ions selected per cycle and dynamic exclusion to minimize repeat precursor ions, can be optimized to identify the extensive lipid molecules. DDA performance, however, is suffered as sample complexity increases due to limited dynamic range and bias toward high abundance ions. The strategy of data-independent acquisition (DIA) has recently received a considerable attention[11,12]. In contrast to DDA, DIA can obtain all fragment ions for all precursors simultaneously, thereby increasing the MS/MS signals and reducing the false negatives. Unfortunately, this approach is not easily applicable to metabolomics because the spectra annotation and false-discovery rate (FDR) evaluation require more sophisticated software to deal with the acquisition data. Furthermore, in a large-scale of lipidomic study, a dataset with good quality and reliability typically undergoes a number of processes, which covers from very beginning of experiment design to data analysis. For instance, in sample collection, the interethnic difference of diabetes is an important factor to be deliberated. Ma and Chan made an epidemic investigation towards diabetes in Asia and Europe, and found that compared with their Europe counterparts, T2D developed in East Asian patients exhibited a lower mean BMI with a greater amount of body fat and a tendency to visceral adiposity[13]. Also East Asian patients with T2D was found at a higher risk of developing renal complications than Europeans. The issue of interethnic diabetes thus prompted us to pursue what is the typical characteristics of lipidomics for the T2D and prediabetes in Chinese population.

With evaluation to these challenges in the diabetes-related lipidomics, we initiated this study through collecting 293 Chinese individuals, including T2D, prediabetes and NGT people, conducting lipidomic profiling using LC-MS/MS and an in-house software, metaX[14], for analysis of multivariate data generated from lipodomics. Our data, for the first time, demonstrated that the Chinese T2D patients possessed significant lipid changes in plasma as compared with the NGT people, and further unveiled that lipid features potentially valuable in prediction of diabetic progress and classification of prediabetes.

Data Description

To survey the lipid metabolites in the normal and diabetic samples, a total of 293 fasting blood samples was collected, including 114 T2D patients, 81 prediabetic individuals and 98 individuals with normal glucose tolerance (NGT) according to the diagnostic criteria of WHO 2011 (Additional file 1). To profile the lipid metabolites in blood samples, the lipids were extracted from plasma, and the individual extracts were injected into Waters LC-MS platform followed by acquisition of mass signals at both positive and negative mode. The raw data was subjected to nonlinear data alignment and normalization by Progenesis QI

2.0 (Nonlinear Dynamics, Newcastle, UK), and was treated with the in-house pipeline to generate lipid profiles. Univariate and multivariate statistical analyses were applied to the lipid profiles to identify and evaluate the significant features among the groups (details in Methods). The quality control (QC) for lipid identification was performed by mixing equal amounts of plasma from all subjects as described in [15] and acquiring mass signals from the pool at the certain intervals (Additional file 2). The raw datasets were deposited in the MetaboLights open access data repository [MTBLS352].

Analyses

The workflow designed for the entire project is illustrated in Fig. 1, which consists of three phases: phase 1 for collecting the information of clinical specimens and acquiring mass signals from LC-MS/MS; phase 2 for extracting the metabolic features through the software of MS data analysis; and phase 3 for identifying the lipid candidates in response to the development of T2D.

Clinical characteristics of the specimens

The clinical information including physiological and biochemical parameters for these specimens was summarized in Table 1. Typical diabetes characteristics showed marked significance among three groups (Kruskal-Wallis test (KW test), $p<0.05$). The levels of FPG, 2h-PG, HbA1c, C-peptide, insulin and insulin resistance (HOMA-IR) index in the specimens of T2D and prediabetes were significant higher than that in NGT, while that in T2D was the highest values compared with prediabetes and NGT (*Dunn's* post hoc test, $p<0.05$). The common biochemical indicators in the T2D plasma, such as TG (triacylglycerol), TC (total cholesterol) and LDL (low-density lipoprotein) levels, were significant higher than that in NGT as well (*Dunn's* post hoc test, $p<0.05$). And all the physiological indexes in NGT, such as age, BMI, waist-hip ratio and systolic blood pressure (SBP), exhibited the lower values as compared with that in T2D (*Dunn's* post hoc test, $p<0.05$). In contrast to prediabetes and NGTs, moreover, the ratio of antihypertensive medication use (Calcium channel blockers, CCBs) in T2D was significantly higher (Chi-square test, $p<0.05$). The clinical evidences were in a good agreement with other observations that suggested advanced age, obesity and hypertension tightly associated with T2D.

Lipidomic analysis on the specimens using LC-MS/MS

Principal component analysis (PCA) was taken to evaluate the data quality using metaX[14]. As shown in Additional file 3, all of the QC samples at different intervals were clustered together, indicating the MS signals highly reproducible. On the basis of analysis with Progenesis QI and metaX, totally 11,077 features with average coefficient of variation (CV) of 14.8% were acquired

at positive ion mode (PIM), and 923 features with average CV of 14.7% were yielded at negative ion mode (NIM) from all the lipid extracts (Additional file 4). Of the features at PIM, approximately 52.83% (5,852 /11,077) were matched to one or more the likelihood of lipids or lipid-like compounds using HMDB (http://www.hmdb.ca/), LIPID MAPS (http://www.lipidmaps.org/) and LipidBlast (http://fiehnlab.ucdavis.edu/projects/LipidBlast), while of the feature at NIM, about 35.43% (327/923) were achieved. Therefore, the results of lipid identification upon LC-MS/MS demonstrated that the lipids extraction from plasma was successful and the identification information was constructive for latter determination of T2D-related lipids. The interactions between the clinical parameters (Additional file 1) and the total features identified from both ion modes (Additional file 2) were further evaluated by PERMANOVA (permutational multivariate analysis of variance). Right before the evaluation, some datasets were considered as the outliers and removed (12 from PIM and 9 from NIM, Additional file 3A, B) by metaX. The data in Additional file 5 summarized the evaluation results derived from PERMANOVA and presented that the total features were significantly interacted with the abundance of some plasma indexes, such as TG, HDL, TC, C-peptide, leptin and HOMA-IR index, and with the physiological conditions, such as gender, waist-hip ratio, BMI and age (FDR<0.05). However, other clinical parameters such as insulin, FPG, LDL, SBP levels and CCBs use, appeared relatively weak interactions with the total features ($p<0.05$ and FDR>0.05).

The T2D-related lipidome features

As CCBs use seemed some effects to the interactions with the total features, its perturbation to lipid profiles was considered in further data analysis. Blocked KW test was thus adopted to estimate the feature comparability among multiple groups, in which CCBs treatment was set as the blocking factor in statistical evaluation. After finding of the differential features by blocked KW test, the selected features between two groups were further assessed through pairwise comparisons by *Dunn's* post hoc test. The results of the blocked KW test were shown in Additional file 6, and of the *Dunn*'s post hoc test were plotted with Venn diagram in Fig. 2. A total of 1,590 features displayed significant differences among the 3 groups, including 1,395 features at PIM and 195 at NIM (Additional file 6, $p<0.05$), and approximately 55.91% (889/1,590) matched to lipids or lipid-like compounds in public databases. As depicted in Fig. 2, the differential features of NGT vs T2D possessed the largest number (1269) in all the pairwise comparisons, whereas 785 differential features were found in NGT vs prediabetes and 578 ones in prediabetes vs T2D. The gradual changes of the differential features from NGT to prediabetes to T2D suggested that the plasma lipids might be treated as the indicators for diabetic development.

To clearly quantify the differential features among these groups, the total features were assessed through the filterers with the criteria of fold change (FC) in mass intensity ≥ 1.2 or ≤ 0.8 and variable importance of the projection (VIP) > 1.0 estimated by Partial Least Squares Discriminant Analysis (PLS-DA). With comparison of quantitative features between T2D and NGT or prediabetes and NGT, 27.80% or 16.42% of the 1,590 features were significantly different in T2D or prediabetes from that in NGT, 229 or 186 up-regulated and 213 or 75 down-regulated (Additional file 6), respectively, while in the comparison between T2D and prediabetes, 7.36% were significantly changed in the two groups with 58 elevated and 59 lowered in T2D. The evidence derived from quantitative comparison supported the conclusion drawn from the blocked KW test that the lipid indicators could reflect the gradual changes of lipid features from NGT to prediabetes and to T2D. Interestingly, about 42.79% (98/229) of the up-regulated lipid features in T2D were overlapped with that in prediabetes, whereas only 18.31% (39/213) of the down-regulated features in T2D were crossover with prediabetes. Thus whether the lipid features with abundance increase in diabetes status are more sensitive to indicate the diabetic development is a direction worth for further study.

The T2D risk evaluation in light of the lipidomic data using Random Forest (RF) classifier

As the qualitative and quantitative analysis revealed the significant differences of lipidomes between T2D or prediabetes and NGT, a question was naturally raised what was an efficient way to utilize such information for evaluation of T2D risk. The random forest (RF) classifier was employed for the purpose. All the specimens of T2D and NGT were randomly divided into two sets, 70 T2D and 70 NGT as the training set and the rest as the validation set. As illustrated in Fig. 3A, the model containing 28 features was successfully generated from RF algorithm. Applying the model to the training set, the prediction accuracy was 86.43% and the AUC (Area under curve) was 90.23% (95% confidence interval (CI) =84.95-95.52%) (Fig. 3B), while that to validation set, a similar prediction result was gained with the accuracy of 80.70 % and the AUC of 86.24% (95% CI= 76.05−96.43%) (Fig. 3C). This implies that the model acquired from RF classifier could well distinguish T2D from NGT. The model therefore was further taken to evaluate T2D risk in another validation sets that contained the same specimen of T2D and NGT as mentioned above but plus 76 untrained prediabetes. As depicted in Fig. 3D-3E, T2D or NGT was basically different from prediabetes with the accuracy of 66.07 % and the AUC of 71.77% (95% CI= 61.95−81.58%) or of 63.91% and the AUC of 68.08 (95% CI= 54.87−81.28%)%. The relevant information of selected features such as retention times (RT), precursor ions (mass to charge ratio, *m/z*), matched compounds and lipid categories, was presented in Additional file 7. Hence, the RF model derived from the lipidomic data was likely to reflect the status of diabetic development. In addition, the risk probability (RP) was assigned to each sample once the model was taken in RF analysis. For instance, in the prediabetes, the median of the RP value for the prediabetes with raised $HbA1c_{5.7\text{-}6.4\%}$ was 0.298, for iIGT was 0.398 and for combined IFG/IGT was 0.494, respectively, in which the RP of raised

$HbA1c_{5.7\text{-}6.4\%}$ was similar to NGT and of combined IFG/IGT was more close to T2D (Fig. 3F). The evidence offered another clue for estimation of T2D risk, the prediabetes sub-classification potentially relied on the RF model.

Statistical analysis to define the correlation of T2D-related lipid compounds and diabetic parameters

Whether is there any individual lipid feature correlated with clinical parameters in these specimens? Analysis of Spearman's rank correlation coefficients was taken to assess the correlations (Fig. 4). Of the 28 selected features derived from the RF model, 20 individual features were strongly correlated with at least one of the diabetes-related indexes, like FPG, 2h-PG, HbA1c, C-peptide, insulin and HOMA-IR levels ($p<0.01$, absolute value of Spearman's correlation coefficient (rho)>0.3). For instance, *m/z 203.0533* at ESI (+) was found in higher positive correlations with FPG, HbA1c and 2h-PG at rho=0.5577, 0.5665 and 0.4135, whereas was observed in less correlations with fasting C-peptide and insulin levels at rho=0.1017 and 0.1827, respectively. PS (38:1) with *m/z 800.5850* at ESI (+) was positively correlated with BMI and insulin ($p<0.01$, rho>0.3), whereas TG (62:9) with *m/z 967.8174* at ESI (+) was negatively correlated with these indexes ($p<0.01$, rho<−0.3). Moreover, both of them displayed weaker correlations with FPG and HbA1c (absolute value of rho<0.3). For the sake of the potential influences of age, gender, BMI and the medicine used to treat hypertension to the lipid features, a general linear model **(**glm) was further conducted. The 26 out of 28 selected features remained significantly different between T2D and NGT after adjustment of the potential influences (adjusted $p<0.05$; Additional file 8). The two metabolites filtrated from the glm, namely NeuAcalpha2-3Galbeta-Cer (d18:1/18:0) with *m/z 1019.7063* at ESI (+) and DG (43:6) with *m/z 1017.692*2 at (ESI (+), were found weak negative correlation with diabetic indexes ($p<0.05$, rho>−0.3). The results combined Spearman's correlation and glm analysis thus revealed that the most lipid features elicited from the RF model had the tight correlations with diabetes. The deduction was further supported by the comparison of relative intensities for the lipid features as shown in Additional file 9 (A-Z). As plotted, the intensities of all features for T2D were significantly different from that for NGT, whereas such values for prediabetes were generally located between the values for T2D and NGT and were not always significantly difference from the either group. The different tendencies of the plasma lipids in prediabetes indicate that the development and progression of T2D is highly complex. The 26 RF selected features were further identified by DDA and classified by Metabolomics Standards Initiative (MSI) according to their degree of physicochemical and/or spectral similarity to available reference lipid standards or to published data[16]. Of the 5 features, the 2 features, which shared similar *m/z* values under ESI (-) and appeared at different RT, were identified as one compound, LysoPC (17:0). Thus totally 4 lipid compounds were annotated upon DDA, hydroxybutyrylcarnitine with *m/z 248.1511* at ESI (+), LysoPC (17:0) with *m/z 508.3404* or *m/z 508.3406* at ESI (-), LysoPC (17:1) with *m/z 506.3249* at ESI (-) and PC (17:2(9Z, 12Z)/0:0)

with *m/z 504.3093* at ESI (-) (Additional file 10-13). The concentrations of hydroxybutyrylcarnitine were dramatically higher in T2D as compared of that in NGT and prediabetes, the highest level in T2D and the lowest level in NGT (Additional file 9C). The concentrations of the other three lipids, LysoPC (17:0), PC (17:2(9Z,12Z)/0:0) and LysoPC (17:1), in NGT and prediabetes were comparable, but were significantly higher than those in T2D (Additional file 9B, 9F, 9P, 9R).

Discussion

Prediabetes, which represents a high-risk condition that precedes the onset of T2D, has attracted significant attention on T2D prevention and pathogenesis research. To evaluate the risk of T2D development in different prediabetes definitions, Morris et al collected the 70 follow-up studies and estimated the progression rates per 1,000 person-years from prediabetes to T2D. The meta-analysis demonstrated that the T2D incidences of 35.54/1000 for HbA1c $_{6.0\text{-}6.4\%}$, 45.46/1000 for iIGT and 70.36/1000 for combined IFG/IGT[17]. As shown in Fig.3F, the prediabetes in this study were basically divided into 3 subgroups, raised HbA1c$_{5.7\text{–}6.4\%}$, iIGT and combined IFG/IGT, and were assessed their diabetes risk with RF algorithm. We reached a conclusion that the risk probability based on lipid features were the lowest for HbA1c $_{5.7\text{-}6.4\%}$ while the highest for combined IFG/IGT. The MS data analysis was in basic agreement with the large-scale clinical observation, and suggested that lipidomic data could reflect some of the different pathological changes during the diabetic development at the prediabetic states. As mentioned in Xu's report, the prediabetes diagnostic performance with HbA1c$_{5.7\text{–}6.4\%}$ was significantly higher than FPG$_{5.6\text{-}6.9\text{mmol/L}}$ or 2h-PG$_{7.8\text{-}11.0\text{mmol/L}}$ in Chinese population[3]. We also observed that lower diabetes risks in prediabetes with HbA1c$_{5.7\text{–}6.4\%}$ and NGT upon the lipomic data as criterion parameters. Both evidences lead to a postulation that prediabetes defined upon HbA1c criterion seems independent from race or country, even though a statistical validation at large cohort is still required.

Annotation of the T2D-related lipid feature acquired from MS/MS is still a long way to go, while the lipids identified in this study offered a strong evidence of which lipids did play an indicative role for diabetic progression. Our findings revealed that plasma lipid levels were altered in response to the T2D progression, accompanying with enhanced acylcarnitines and declined lysophosphatidylcholines. At MSI level 2, hydroxybutyrylcarnitine with *m/z 248.1511* at ESI (+) (Additional file 9C) was found a significantly elevated abundance during progression from NGT to prediabetes to T2D (KW test, *p*=2.39E10-9, FDR=1.32E10-5, Additional file 6). Generally D-3-hydroxybutyrylcarnitine could be converted to D-3-hydroxybutyric acid, which is the predominant ketone body present in diabetic ketoacidosis [18–20]. Notably at MSI level 4, hydroxybutyric acid with *m/z 103.0391* at ESI (-) (Additional file 9I) was also observed much higher level in T2D (KW test, *p*=2.38E10-8, FDR=1.01E10-5, Additional file 6) in this study. As a matter of fact, hydroxybutyrylcarnitine is a metabolite closely associated with insulin resistance and

T2D, and increased D-3-hydroxybutyric acid has been suggested as an early biomarker of insulin resistance [18]. In addition, both T2D and prediabetes displayed relatively higher concentrations of several short- and long-chain AcylCNs, such as L-acetylcarnitine (C2) with *m/z 203.1160n* (*p*=6.66E10-4, FDR=0.066), tetradecanoylcarnitine (C14) with *m/z 372.3115* (*p*=1.46E10-3, FDR=0.094), 2-hydroxyhexadecanoylcarnitine (C16OH) with *m/z 416.3371* (*p*=1.60E10-4, FDR=0.044), and 12-hydroxy-12-octadecanoylcarnitine (C18OH) with *m/z 444.3667* (*p*=5.70E10-4, FDR=0.066), with all detected at PIM (KW test, Additional file 6). Most acylcarnitines are derived from fatty acid oxidation, while plasma acylcarnitines are largely contributed from muscle or liver and delivered to circulation system through their transporters across cell membrane. The plasma acetylcarnitine levels were reported significant positive correlation with HbA1c levels over a wide range of insulin sensitivity, suggesting upregulation of carnitine acetyl-CoA transferase in the mitochondrial of insulin-resistant tissues[21]. The increased acylcarnitine concentrations in the plasma of T2D and prediabetes were reported in German population as well[22]. Lysophosphatidylcholine (LysoPC) is a major phospholipid component of oxidized low-density lipoprotein, and is implicated as a critical factor in diabetes. A particularly interesting finding was that a number of triacylglycerol and diacylglycerol species were elevated along with of a number of sphingolipids in diabetes, whereas LysoPC levels were reduced[23]. The lipidomic measurement in this study supported the observation. As compared with NGT, the abundance of LysoPC (17:0) with *m/z 508.3404* at ESI (-) (*p*=2.93E10-5, FDR=1.88E-03) and *m/z 508.3406* at ESI (-) (*p*=3.01E10-7, FDR=5.55E-05) was observed a significant reduction in T2D individuals (KW test, Additional file 6), and PC (17:2(9Z,12Z)/0:0) with *m/z 504.3093* at ESI (-) (*p*=6.28E10-5, FDR=3.41E-03) and LysoPC (17:1) with *m/z 506.3249* at ESI (-) (*p*=7.95E10-6, FDR=6.67E-04) were found lower level in diabetic patients (KW test, Additional file 6). Although alterations in species of LysoPC and their PC precursors are commonly accepted in diabetic and obese status, the regulatory mechanisms underlying the phenomenon have remained largely unclear. Using LC MS/MS, we identified several LysoPC compounds that had not been reported by others. Therefore, lipidomic analysis could offer more lipid candidates, including LysoPCs, which are likely to be informative data for mechanism study of LysoPC metabolism.

In addition, we observed that the CCBs treatment (n=61) for hypertension showed slight effects on plasma lipid profiles (*p*=0.0324 and FDR=0.0825) by PERMANOVA analysis (Additional file 5), which was consistent with previous report[24]. No significant effects were found in other medication records, which may probably because of smaller sample sizes than CCBs or the limited impacts they indeed had (Additional file 5). Although further validation is required for drug effects, we suggest that the medications, as the reported interfering factors on lipid metabolism[24,25], should be carefully considered in lipidomics-

based investigations. Hence, we used strict statistical analyses to control for possible disturbances of treatment on reporting of T2D-related plasma lipids.

In summary, by using the LC-MS/MS based untargeted lipidomics analysis, our study is the first large-scale study to explore the alterations in the plasma lipid patterns in individuals with NGT, different prediabetic states and T2D from East China. We describe greater number of plasma lipids than previous reports, providing a broad coverage of major lipid categories. We identify thousands of plasma lipids with remarkable difference among three diagnostic groups, with large members displaying similar trends in prediabetes and type 2 diabetes. Additionally, we observed the stratification of predicted diabetes risk among subgroups of prediabetes based on 28 selected plasma lipids. Several of the diabetic-related candidates have not previously been reported. Together, this study improves disease prediction and provides a better biological understanding of the insidious progression to diabetes from a lipid perspective. More comprehensive studies combining genomics, metabolomics, proteomics and metagenomics should be conducted to describe the detailed variations among prediabetes subgroups and to support precise prevention and intervention steps for T2D.

Methods

Participant recruitment, sampling and grouping

Total 433 participants submitted written informed consent form, and have been entered from the community health service centers of Suzhou Center for Disease Prevention and Control (CDC). All participants were processed the two-steps enrollment criteria. At the first visit time, all participants obtained physical examinations including height, weight, blood pressure, waist and hip circumference and completed a face-to-face questionnaire on demographics, medication history, family health history and other lifestyle factors via well-trained local staffs. The study only enrolled the participants who met those criteria based on questionnaire, including 1) age 40 or older; 2) free of cardiovascular disease, severe renal disease, cancer, type 1 or monogenic diabetes and other autoimmune diseases, as determined by self-report; and 3) no antibiotic use during the past 2 months. Approximately 81.7% of the participants in the cohort (354 out of 433) met above criteria, had been asked to attend the blood screening tests for diabetes according to the 2011 WHO criteria[26]. The qualified participants without a self-reported history of type 2 diabetes were administered a 2-hour 75 g oral glucose tolerance test (OGTT). Participants with fasting or postprandial blood glucose levels above the diagnostic cut-off point were asked to repeat the corresponding test on the next day. Blood medical tests were performed by a Nanjing Kingmed Center for Clinical Laboratory, which included FPG, insulin, C-peptide, HbA1c, leptin, adiponectin, and blood lipid levels in addition to routine blood tests. The fasting plasma would be collected within 1 hour after blood withdrawal by centrifuged at 1,600 g for 15 minutes. The upper layers were carefully collected to avoid disturbing the buffy coat cells. The isolated plasma samples were stored at -80°C and transported on dry ice to BGI-Shenzhen.

Finally, a total of 293 subjects were definitively divided into 3 diagnostic groups, namely the normal glucose tolerance group (NGT, n=98), the prediabetes group (Pre-DM, n=81) and the type 2 diabetes group (T2D, n=114; including 77 newly diagnosed and 37 self-reported patients). The Pre-DM samples were further classified into 4 subgroups: a) raised HbA1c $_{5.7\text{-}6.4\%}$ (defined by the WHO-HbA1c criteria only; n=15); b) isolated IFG (defined by an FPG level of 6.1-7.0 mmol/l and a normal 2h-PG level; n=7); c) isolated IGT (defined by a normal FPG level and a 2h-PG level of 7.8–11.0 mmol/l; n=35); and d) combined IFG/IGT (defined by an FPG of 6.1-7.0 mmol/l and a 2h-PG level of 7.8–11.0 mmol/l; n=24). The study was approved by the Institutional Review Board of BGI-Shenzhen and the ethical review committee of Suzhou CDC.

Lipidome data processing and analysis (Lipidomics)

1. Lipid preparation and extraction

The collected plasma samples were thawed on ice, and lipids were extracted with isopropanol (IPA) using a previously described method[12]. Briefly, 40 μL of plasma was extracted with 120 μL of precooled IPA, vortexed for 1 min, and incubated at room temperature for 10 min; the extraction mixture was then stored overnight at -20°C. After centrifugation at 4,000 g for 20 min, the supernatants were transferred into new 96-well plates and diluted to 1:10 with IPA/acetonitrile (ACN)/H_2O (2:1:1, v:v:v). The samples were stored at -80°C prior to the LC-MS analysis. In addition, pooled plasma samples were also prepared by combining 10 μL of each extraction mixture.

2. UPLC-MS method for lipidomics

Samples were analyzed with an ACQUITY UPLC (Waters, Manchester, USA) connected to a XEVO-G2XS QTOF mass spectrometer (Waters) with electrospray ionization (ESI). The lipids were separated using an Acquity UPLC CSH C18 column (2.1×100 mm, 1.7 μm, Waters) with a gradient mobile phase comprised of 10 mM ammonium formate with 0.1% formic acid in acetonitrile/water (A, 60:40, v/v) and 10 mM ammonium formate with 0.1% formic acid in isopropanol/acetonitrile (B, 90:10, v/v). The mobile phase was delivered at a flow rate of 0.4 mL/min. The column was initially eluted with 40% B, followed by a linear gradient to 43% B over 2 min, and then the percentage of B was increased to 50% within 0.1 min. Over the next 3.9 min, the gradient was further ramped to 54% B, and the amount of B was then increased to 70% in 0.1 min. In the final part of the gradient, the amount of B was increased to 99% over 1.9 min. Finally, solution B was returned to 40% in 0.1 min, and the column was equilibrated for 1.9 min before the next injection. The injection volume was 10 μL. Lipids were detected with a XEVO-G2XS QTOF mass spectrometer in positive and negative mode, which was operated in MS^E mode from *m/z 50-2,000*, with an acquisition time of 1 s per scan. The source temperature was set at 120°C. The desolvation temperature and gas flow were 600°C and 800 L/h, respectively, and nitrogen was used as the flow gas. The capillary and cone voltages were 2.0 kV (+) / 1.5 kV (-) and 30 V, respectively. Leucine encephalin (molecular weight (MW) = 555.62; 200 pg/μL in 1:1 ACN:H_2O) was used as a lock mass for accurate mass measurements, and 0.5 mM sodium formate solution was used for calibration. The samples were randomly ordered, and 10 QC samples were initially injected to condition the column. One QC sample was injected and analyzed every 10 samples to investigate the repeatability of the data[27].

3. Acquisition of the high quality non-targeted metabolic profile and Metabolite identification

The raw UPLC-MS data were imported into Progenesis QI 2.0 data analysis software (Nonlinear Dynamics, Newcastle, UK) for peak selection and alignment. The intensity of each peak was normalized to all compounds, and peak intensities for the retention times and m/z data pairs were produced. The retention time (0.5-9.0 min) and peak width (1 s-30 s) tolerance were

configured with Progenesis QI to reduce low quality peaks and to produce comparable peaks. The normalized peak data was further preprocessed by an in-house software metaX [14].

Those features that were detected in less than 50% of QC samples or 80% of biological samples were removed, the remaining peaks with missing values were imputed with kNN (k-Nearest Neighbor) algorithm to further improve the data quality. PCA was used to detect outliers and was performed with metaX using the pre-processed dataset. QC-RLSC (quality control–based robust LOESS signal correction) implemented in metaX, which was fitted to the QC data with respect to the order of injection, was conducted to minimize signal intensity drift over time. In addition, the relative standard deviations (RSDs) of the metabolic features were calculated across all QC samples. The features with RSDs >30% were then removed.

The metabolites were identified using Progenesis Metascope based on the HMDB (version 3.6) (http://www.hmdb.ca/), LIPID MAPS (http://www.lipidmaps.org/) and LipidBlast (http://fiehnlab.ucdavis.edu/projects/LipidBlast), with a mass tolerance of 10 ppm for the precursors. The metabolites that could be matched to LIPID MAPS, LipidBlast or the aliphatic compounds of HMDB at the Molecular Framework level were considered lipids and lipid-like features. Data-dependent analysis (DDA), which covered the desired mass scan range of interest, was performed to further aid in identifying the metabolites. The reported molecules were classified into Metabolomics Standards Initiative (MSI) levels according to the reported guidelines[16].

4. Data analysis

4.1 Univariate analysis: Kruskal-Wallis testing and fold change analysis

Blocked Kruskal-Wallis tests were conducted to detect differences in metabolite concentrations among the 3 diagnostic groups after controlling for the potential confounding effects of CCB drugs. The analysis was performed using the tools implemented in the COIN software package (coin 1.1-2 in R 3.2.5). The *p* value was adjusted for multiple tests using an FDR (Benjamini-Hochberg). *Dunn's* post hoc tests followed by pairwise comparisons were performed; "=" indicates no significant difference and ">" indicates *p* values <0.05. Fold changes were calculated by comparing the mean concentrations of each feature between groups.

4.2 Multivariate analysis

To improve the performance of the subsequent statistical analyses, all features were normalized to the range of [0, 1] to stabilize the variance using a modified range-scaling method with the following formula [28]:

$$\tilde{x}_{ij} = \frac{x_{ij} - x_{i_{min}}}{(x_{i_{max}} - x_{i_{min}})}$$

where $\tilde{x}_{ij}$ indicates the scaled value for *i*-th variable (compound) in the *j*-th sample (which is valued by x_{ij},) and $x_{i_{max}}$ and $x_{i_{min}}$ represent the maximum and minimum values for the *i*-th variable among the samples, respectively.

PERMANOVA for the influence of clinical and lifestyle factors

Permutational multivariate analysis of variance (PERMANOVA) was performed on the normalized lipid metabolite profiles using the adonis function with the bray distance implemented in the vegan package (vegan 2.4-0 in R 3.2.5, https://cran.r-project.org, https://github.com/vegandevs/vegan). The number of permutations was 9,999. The *p* value was corrected for multiple tests using an FDR (Benjamini-Hochberg) cut-off of 0.05.

PLS-DA analysis

A supervised partial least-squares discriminant analysis (PLS-DA) was conducted through metaX to discriminate between the different variables between groups. The variable importance of the projection (VIP) value was calculated. A VIP cut-off value of 1.0 was used to select important features.

Random forest (ROC/AUC) analysis

The RF classifier (randomForest 4.6-12 in R 3.2.5) was trained on 140 randomly selected subjects (70 NGT and 70 T2D) from the 273 samples and then tested on the remaining subjects. All of the features were supplied to the classifier. The analysis was conducted with 5 repetitions of the 10-fold cross-validation, using cross-validational error curves to selected features as described by Feng et al[29]. The risk probability (RP) of T2D was assessed using the selected features and a receiver operation characteristic (ROC) curve was drawn, for which the AUC was calculated (pROC1.8 in R 3.2.5). The selection frequencies of features were listed to measure the importance of the variables, with a higher frequency indicating the greater importance of a given metabolite for classifying T2D and NGT. The RF model was further tested on the validation sets.

4.3 Correlation analysis and analysis of the general linear model

Spearman's rank correlations between metabolites or between the phenotypes and metabolites were calculated with R 3.2.5. The R package *q* value was used to generate *q* values for each Spearman correlation. A general linear model was applied to each selected metabolite. Putative confounding factors, including age, BMI, gender and 2 major antihypertension drugs, namely CCBs (61 people used these drugs) and angiotensin receptor blockers (20 people used these drugs), were added as covariates in the glm analysis. The *p* value of the coefficient was calculated using the glm function in R 3.2.5.

Availability of supporting data

The data supporting this article is available in the MetaboLights [MTBLS352].

List of abbreviations used

T2D: Type 2 diabetes
Pre-DM: prediabetes
NGT: Normal glucose tolerant
AUC: area under the curve
HbA1c: glycated hemoglobin
(i)IGT: (isolated) impaired glucose tolerance
(i)IFG: (isolated) impaired fasting glucose
WHO: World Health Organization
ADA: American Diabetes Association
2h-PG: 2-hour postprandial glucose
FPG: fasting plasma glucose
LC-MS/MS: liquid chromatography/mass spectrometry
GC/MS: gas chromatography/mass spectrometry
FFA: free fatty acid
DDA: data-dependent analysis
DIA: data-independent acquisition
FDR: false discovery rate
BMI: body mass index
QC: quality control
KW test: Kruskal-Wallis test
HOMA-IR: insulin resistance index
TG: triacylglycerol
TC: total cholesterol
LDL: low-density lipoprotein
SBP: systolic blood pressure
DBP: diastolic blood pressure
CCB: calcium channel blocker
PCA: Principal components analysis
CV: coefficient of variation
PIM: positive ion mode
NIM: negative ion mode
PERMANOVA: Permutational multivariate analysis of variance
HDL: high-density lipoprotein
FC: fold change
VIP: Variable importance of the projection
PLS-DA: Partial Least Squares Discriminant Analysis
RF: random forest
CI: Confidence Interval
RT: retention times
RP: risk probability
ESI: electrospray ionization
Glm: general linear model
MSI: Metabolomics Standards Initiative
LysoPC: lysophosphatidylcholine
OGTT: oral glucose tolerance test
IPA: isopropanol
ACN: acetonitrile
UPLC-MS: Ultra Performance Liquid Chromatography-Tandem Mass Spectrometry
MW: molecular weight
KNN: k-Nearest Neighbor
QC-RLSC: quality control–based robust LOESS signal correction
RSDs: relative standard deviations
ROC：Receiver-Operating Characteristic

Competing financial interests

The authors declare no competing interests.

Author contributions

J.L., S.L. and C.N. conceived and directed the project. Y.L, G.Z., J.C., Y.H., Y.G. and J.Z oversaw the sample collection and provided phenotypic information. J.L., and B.W. routinely managed the project at BGI-Shenzhen. G.H., J.Z. and Y.F. contributed to the experiment. H.Z., C.F., Y.F., F.Y., H.R., J.W., Z.Y. and Y.P. performed the bioinformatic analyses, and prepared figures and texts for manuscript. H.Z. and S.L. wrote the manuscript. All authors contributed to the revision of the manuscript.

Acknowledgments

We thank all the volunteers participating in this study, the staffs from Suzhou CDC and its affiliated organizations for collecting samples and physical and daily phenotypes. This study was supported by the Shenzhen Municipal Government of China (JSGG20160229172752028, JSGG20140702161403250, CXB201108250098A, DRC-SZ [2015]162) and Suzhou Biobank (SS201111). And we gratefully acknowledge colleagues at BGI-Shenzhen for lipid extraction, LC/MS analysis and helpful discussions. We also thank Professor Juergen Graessler and his colleagues from Dresden University of Technology for their useful suggestion on lipid identification.

References

1. Stumvoll M, Goldstein BJ, van Haeften TW. Type 2 diabetes: principles of pathogenesis and therapy. Lancet. 2010;365:1333–46.

2. Yang W, Lu J, Weng J, Jia W, Ji L, Xiao J, et al. Prevalence of diabetes among men and women in China. N. Engl. J. Med. 2010;362:1090–101.

3. Xu Y, Wang L, He J, Bi Y, Li M, Wang T, et al. Prevalence and control of diabetes in Chinese adults. Jama. 2013;310:948–59.

4. WHO. Definition and Diagnosis of Diabetes Mellitus and Intermediate Hyperglycemia. Who22006;50.

5. Association AD. Diagnosis and Classification of Diabetes Mellitus. Diabetes Care. 2006;29:43–8.

6. Wang TJ, Larson MG, Vasan RS, Cheng S, Rhee EP, McCabe E, et al. Metabolite profiles and the risk of developing diabetes. Nat. Med. 2011;17:448–53.

7. Mihalik SJ, Michaliszyn SF, de las Heras J, Bacha F, Lee S, Chace DH, et al. Metabolomic profiling of fatty acid and amino acid metabolism in youth with obesity and type 2 diabetes: evidence for enhanced mitochondrial oxidation. Diabetes Care. 2012;35:605–11.

8. Meikle PJ, Wong G, Barlow CK, Weir JM, Greeve MA, MacIntosh GL, et al. Plasma Lipid Profiling Shows Similar Associations with Prediabetes and Type 2 Diabetes. PLoS One. 2013;8.

9. Drogan D, Dunn WB, Lin W, Buijsse B, Schulze MB, Langenberg C, et al. Untargeted Metabolic Profiling Identifies Altered Serum Metabolites of Type 2 Diabetes Mellitus in a Prospective, Nested Case Control Study. Clin. Chem. 2015;61:487–97.

10. Fiehn O, Timothy Garvey W, Newman JW, Lok KH, Hoppel CL, Adams SH. Plasma metabolomic profiles reflective of glucose homeostasis in non-diabetic and type 2 diabetic obese African-American women. PLoS One. 2010;5:1–10.

11. Mapstone M, Cheema AK, Fiandaca MS, Zhong X, Mhyre TR, MacArthur LH, et al. Plasma phospholipids identify antecedent memory impairment in older adults. Nat. Med. 2014;20:415–8.

12. Cai X, Perttula K, Pajouh SK, Hubbard A, Nomura DK, Rappaport SM. Untargeted lipidomic profiling of human plasma reveals differences due to race, gender and smoking status. Metabolomics Open Access. OMICS International; 2014;2014.

13. Ma RCW, Chan JCN. Type 2 diabetes in East Asians : similarities and differences with populations in Europe and the United States. 2013;1281:64–91.

14. Wen B, Mei Z, Broadhurst DI, Zeng C, Liu S. metaX: a flexible and comprehensive software for processing metabolomics data. under Submitt. 2016.

15. Sarafian MH, Gaudin M, Lewis MR, Martin FP, Holmes E, Nicholson JK, et al. Objective set of criteria for optimization of sample preparation procedures for ultra-high throughput untargeted blood plasma lipid profiling by ultra performance liquid chromatography-mass spectrometry. Anal. Chem. 2014;86:5766–74.

16. Sumner LW, Amberg A, Barrett D, Beale MH, Beger R, Daykin CA, et al. Proposed minimum reporting standards for chemical analysis. Metabolomics. 2007;3:211–21.

17. Morris DH, Khunti K, Achana F, Srinivasan B, Gray LJ, Davies MJ, et al. Progression rates from HbA1c 6.0-6.4% and other prediabetes definitions to type 2 diabetes: A meta-analysis. Diabetologia. 2013;56:1489–93.

18. An J, Muoio DM, Shiota M, Fujimoto Y, Cline GW, Shulman GI, et al. Hepatic expression of malonyl-CoA decarboxylase reverses muscle, liver and whole-animal insulin resistance. Nat. Med. 2004;10:268–74.

19. Hack A, Busch V, Pascher B, Busch R, Bieger I, Gempel K, et al. Monitoring of ketogenic diet for carnitine metabolites by subcutaneous microdialysis. Pediatr. Res.. 2006;60:93–6.

20. Soeters MR, Serlie MJ, Sauerwein HP, Duran M, Ruiter JP, Kulik W, et al. Characterization of D-3-hydroxybutyrylcarnitine (ketocarnitine): An identified ketosis-induced metabolite. Metabolism. 2012;61:966–73.

21. Adams SH, Hoppel CL, Lok KH, Zhao L, Wong SW, Minkler PE, et al. Plasma Acylcarnitine Profiles Suggest Incomplete Long-Chain Fatty Acid b-Oxidation and Altered Tricarboxylic Acid Cycle Activity in Type 2 Diabetic African-American Women 1–3. J. Nutr. Genomics Proteomics, Metabolomics J. Nutr. 2009;139:1073–81.

22. Mai M, T??njes A, Kovacs P, Stumvoll M, Fiedler GM, Leichtle AB. Serum levels of acylcarnitines are altered in prediabetic conditions. PLoS One. 2013;8.

23. Barber MN, Risis S, Yang C, Meikle PJ, Staples M, Febbraio MA, et al. Plasma lysophosphatidylcholine levels are reduced in obesity and type 2 diabetes. PLoS One. 2012;7.

24. Kaur M, Kaur K, Bedi GK, Sidhu GS, Sikand R. Effect of Felodipine on the Serum Lipid Profile of Patients With Hypertension. 2000;15:63–7.

25. Zhang Y, Hu C, Hong J, Zeng J, Lai S, Lv A, et al. Lipid profiling reveals different therapeutic effects of metformin and glipizide in patients with type 2 diabetes and coronary artery disease. Diabetes Care. 2014;37:2804–12.

26. Report A, Consultation WHO. Use of glycated haemoglobin (HbA1c) in the diagnosis of diabetes mellitus. Diabetes Res. Clin. Pract. 2011;93:299–309.

27. Want EJ, Wilson ID, Gika H, Theodoridis G, Plumb RS, Shockcor J, et al. Global metabolic profiling procedures for urine using UPLC-MS. Nat. Protoc. 2010;5:1005–18.

28. van den Berg R a, Hoefsloot HCJ, Westerhuis J a, Smilde AK, van der Werf MJ. Centering, scaling, and transformations: improving the biological information content of metabolomics data. BMC Genomics. 2006;7:142.

29. Feng Q, Liang S, Jia H, Stadlmayr A, Tang L, Lan Z, et al. Gut microbiome development along the colorectal adenoma-carcinoma sequence. Nat. Commun. 2015;6:6528.

Tables and captions

Table 1 Baseline characteristics in three groups of the study

**Variables**	**T2D (n =114)**	**Prediabetes (n = 81)**	**NGT (n=98)**	***p*-value (Kruskal-Wallis test)**
Age, year	65.11 ± 8.77	61.99 ± 8.48	59.11 ± 9.15	4.52E-06
BMI	25.25 ± 3.14	25.23 ± 3.1	24.23 ± 3.26	0.0425
waist /hip Ratio	0.92 ± 0.06	0.91 ± 0.06	0.89 ± 0.06	0.0166
FPG, mmol/l	7.87 ± 1.99	5.88 ± 0.55	5.34 ± 0.36	2.20E-16
2h-PG, mmol/l	15.1 ± 3.76	8.21 ± 1.63	6.01 ± 1.01	2.20E-16
HbA1c, % mmol/mol	7.51 ± 2.06	5.51 ± 0.54	5.04 ± 0.42	2.20E-16
Insulin, ulU/ml	8.85 ± 3.51	8.27 ± 3.75	7.39 ± 2.93	0.0017
C-Peptide, ng/ml	2.31 ± 0.99	2.07 ± 0.85	1.75 ± 0.69	4.55E-05
HOMA-IR	3.13 ± 1.57	2.17 ± 1.03	1.76 ± 0.74	2.20E-16
SBP, mm Hg	136.6 ± 23.51	130.91 ± 15.65	123.28 ± 16.91	3.69E-08
DBP, mm Hg	79.73 ± 12.31	80.37 ± 7.52	77.35 ± 8.84	0.0345
TG, mmol/l	2.04 ± 1.55	1.9 ± 1.19	1.55 ± 0.91	0.0051
CHO, mmol/l	5.4 ± 1.39	5.22 ± 1.18	5.17 ± 1.43	0.2418
LDL, mmol/l	3.86 ± 3.36	3.17 ± 2.00	2.56 ± 1.21	0.0014
HDL, mmol/l	1.16 ± 0.35	1.14 ± 0.37	1.2 ± 0.32	0.2891
Leptin, ng/ml	5.09 ± 1.91	4.29 ± 2.1	4.56 ± 1.67	0.0066
GAD-Ab, lU/ml	13.66 ± 14.61	14.05 ± 13.14	12.92 ± 17.45	0.0660
HsCRP, mg/l	2.62 ± 2.4	2.17 ± 1.81	2.16 ± 1.84	0.4485
Adiponectin, ng/ml	37.41 ± 13.53	37.91 ± 16.57	39.2 ± 13.18	0.5321
				***p*-value (Chi-square test)**
Gender (Female, n (%))	68 (59.65%)	40 (49.38%)	66 (67.35%)	0.0513
Smoking, n (%)	18 (15.80%)	24 (29.63%)	17 (17.35%)	0.068
Hypertension, n (%)	52 (45.61%)	38 (46.91%)	21 (21.43%)	0.0002
CCBs use[#], n (%)	29 (25.43%)	20 (24.69%)	12 (12.24%)	0.0372
Alcohol Drinking, n (%)	12 (10.53%)	19 (23.46%)	12 (12.24%)	0.0769
Hyperlipidemia, n (%)	10 (8.77%)	11 (13.58%)	5 (5.10%)	0.1497

Values are given as mean ± SD or number of patients (%).

#：CCBs, Calcium channel blockers

Figures and captions

Figure 1 Flowchart for participant recruitment and data processing

The study began with the recruitment of participants based on the 2011 WHO criteria for diabetes and prediabetes diagnoses. Blood and clinical data were acquired from 293 qualifying subjects, and untargeted lipidomics LC-MS data were obtained. The raw data were then preprocessed with Progenesis QI to extract metabolic features. Unqualified variables and samples were detected and discarded using the BGI in-house program metaX[14]. Several types of statistical analyses were performed to identify metabolites that differed significantly between the diagnostic groups. The lipid compounds selected by the RF classifiers were identified by matching their accurate masses and MS^E ion spectral fragmentation patterns to those in the database. Data-dependent analysis (DDA) was applied to improve the resolving power for identification. See the Methods for more details.

Figure 2 Venn diagram of significant metabolites from the 3 pairwise comparisons

The Venn diagram depicts the number of significant metabolic features from 3 pairwise comparisons and notes the overlap (the direction of change was ignored, $p<0.05$, *Dunn's* post hoc test).

Figure 3 Random forest classification based on untargeted lipidomics data

(A) Distribution of 5 trials of 10-fold cross-validation error in RF classifiers. The model was trained using relative intensity of the detected features from both PIM and NIM in the trained NGT and T2D (n =70 and 70). The black solid curve indicates average of the five trials (dash lines). The pink line marks the number of selected features in the optimal set. (B) Receiver operating curve (ROC) and area under receiver operating curve (AUC) for the training set. (C-E) ROC and AUC for validation set with NGT and T2D (n=21 and 36), prediabetes and T2D (n=76 and 36), NGT and prediabetes (n= 21 and 76), respectively. (F) Box-and-whisker plot presents the risk probability of developing T2D among the validated NGT (n=21), subgroups of prediabetes including raised $HbA1c_{5.7\text{-}6.4\%}$ (n=15) to iIGT (n=32) to combined IFG/IGT (n=23), and T2D (n=36) according to the RF model.

Figure 4 Spearman's correlations between the phenotypes and 28 RF selected features

Hierarchically clustered heatmap showing the Spearman's rank sum correlations between the levels of the 28 significant metabolites and the phenotypes. Red indicates positive correlations and blue indicates negative correlations. The asterisk (*)

denotes a q value of <0.01 for each Spearman correlation. The cross (+) denotes a p value of <0.01 and a q value of ≥0.01 and. The space denotes a p value of ≥0.01.

Additional file 1 Phenotypic and clinical information for 293 enrolled subjects

Additional file 2 Batch numbers and run orders for biological samples and QCs

Additional file 3 Principal components analysis of plasma lipid profiling from biological samples and QCs

Principal components analysis (PCA) was performed on all samples to identify run outliers and check for possible batch effects in both positive (A) and negative modes (B). The colors represent the different sample classes: green for NGT, blue for prediabetes (Pre-DM), red for T2D, orange for QC and black for outlier.

Additional file 4 Detailed list of total detected plasma features

Additional file 5 PERMANOVA of the influence of clinical records or life habits on lipid profile

Additional file 6 Detailed list of significant features among three groups

Additional file 7 Detailed list of 28 metabolic features selected by random forest classifier

Additional file 8 A multivariate generalized linear model (GLM) analysis on 28 selected features and type 2 diabetes

Additional file 9 Box plot displays the intensity levels of 26 selected diabetic-related features in the normal glucose tolerance (NGT), prediabetes (Pre-DM) and type 2 diabetes (T2D)

The features are presented in an order of decreasing importance according to the selection frequencies in RF model. Asterisks (*) denote p<0.05 (*Dunn's* post hoc test).

Additional file 10 LC-MS/MS spectrum of *m/z 248.1511* and its estimated chemical structure

A precursor ion at *m/z 248.1511* $[M+H]^+$ was detected in the positive ion mode. Each arrow indicates a possible site of fragmentation for the corresponding ion of hydroxybutyrylcarnitine.

Additional file 11 LC-MS/MS spectrum of *m/z 508. 3404* (RT=1.70min) and *m/z 508.3406* (RT=1.83min) and their estimated chemical structure

Precursor ions at *m/z 508.3404* $[M-H]^-$ and *m/z 508.3406* $[M-H]^-$ were detected in the negative ion mode. Each arrow indicates a possible site of fragmentation for the corresponding ion of LysoPC (17:0).

Additional file 12 LC-MS/MS spectrum of *m/z 506.3249* and its estimated chemical structure

A precursor ion at *m/z 506.3249* [M-H]$^-$ was detected in the negative ion mode. Each arrow indicates a possible site of fragmentation for the corresponding ion of LysoPC (17:1).

Additional file 13 LC-MS/MS spectrum of *m/z 504.3093* and its estimated chemical structure

A precursor ion at *m/z 504.3093* [M-H]$^-$ was detected in the negative ion mode. Each arrow indicates a possible site of fragmentation for the corresponding ion of PC (17:2(9Z, 12Z)/0:0).

Figure 1

Click here to download Figure Figure 1.pdf

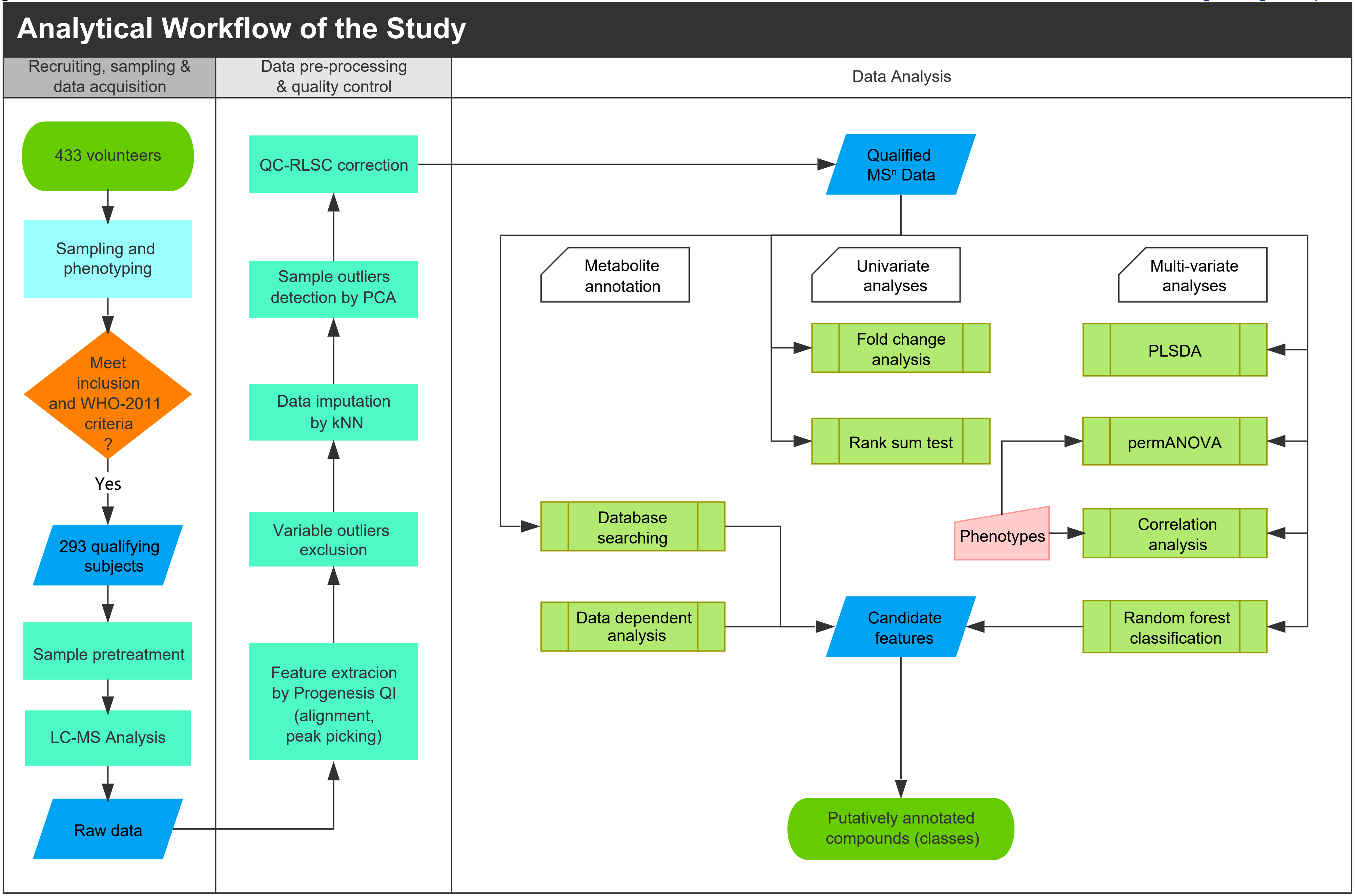

Figure 2

Click here to download
Figure Figure 2.pdf

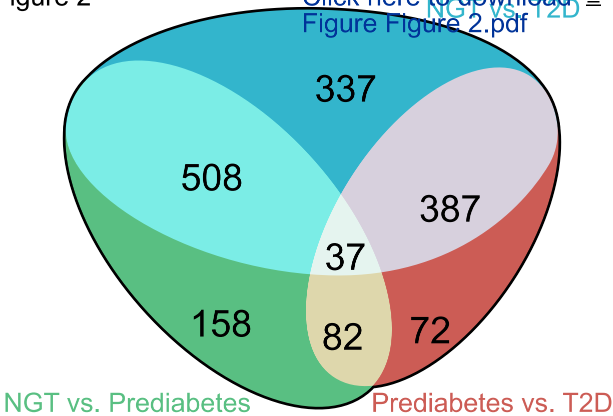

Figure 3

Click here to download Figure Figure 3.pdf

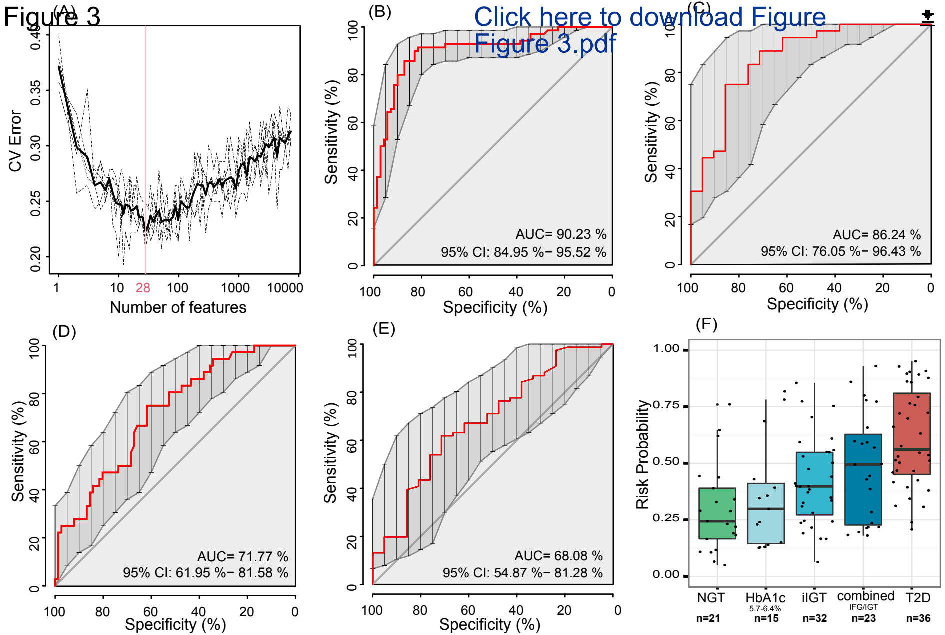

Figure 4

Click here to download Figure Figure 4.pdf

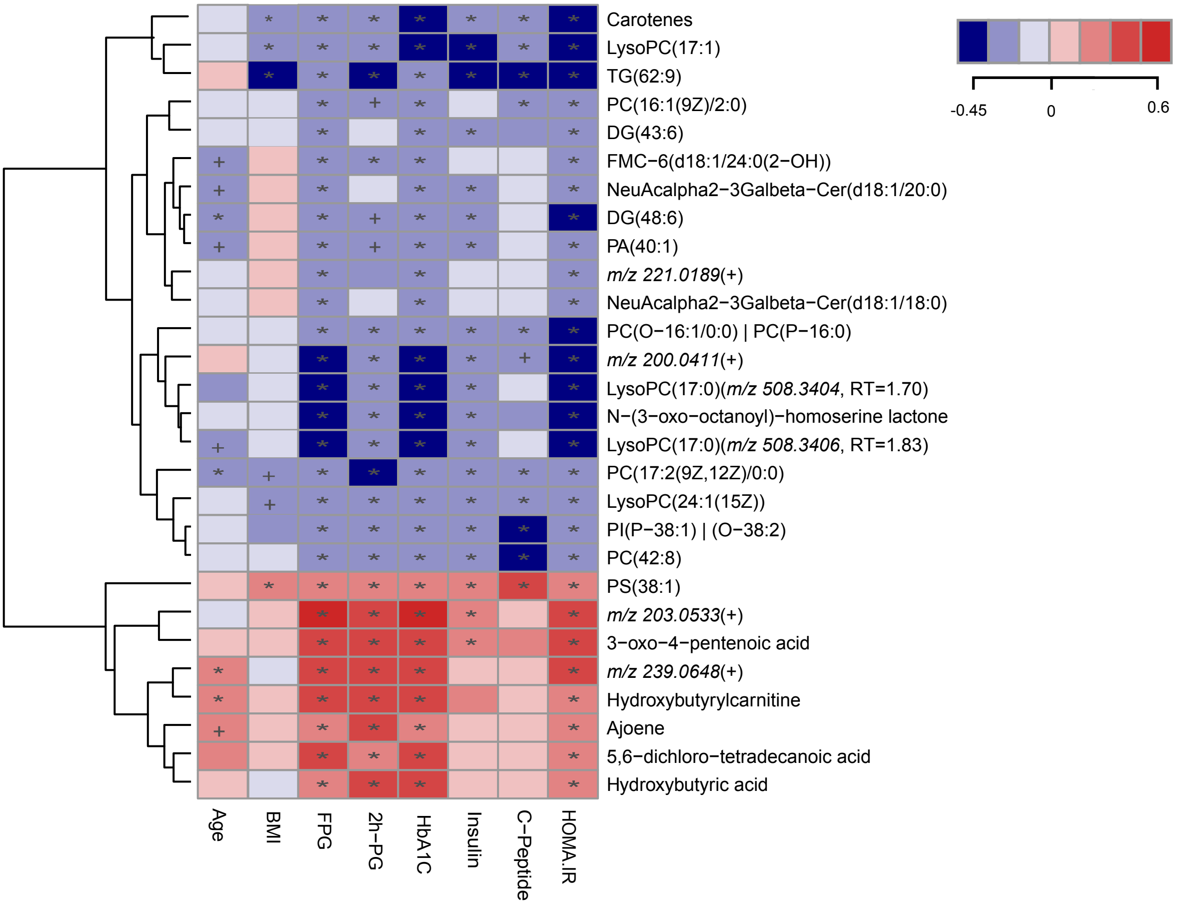

Addtional file 1

Click here to access/download
Supplementary Material
Additional file 1.xlsx

Addtional file 2

Click here to access/download
Supplementary Material
Additional file 2.xlsx

Addtional file 3

Click here to access/download

Supplementary Material

Additional file 3.pdf

Addtional file 4

Click here to access/download
Supplementary Material
Additional file 4 final.xlsx

Addtional file 5

Click here to access/download
Supplementary Material
Additional file 5.xlsx

Addtional file 6

Click here to access/download
Supplementary Material
Additional file 6-final.xlsx

Addtional file 7

Click here to access/download
Supplementary Material
Additional file 7.xlsx

Addtional file 8

Click here to access/download
Supplementary Material
Additional file 8.xlsx

Addtional file 9

Click here to access/download
Supplementary Material
Additional file 9.pdf

Click here to access/download
Supplementary Material
Additional file 10-13.docx

Cover letter

Click here to access/download
Supplementary Material
Coverletter-GigaScience.docx
