# Supplementary material for: Lipidomic profiling reveals distinct differences in plasma lipid composition in healthy, prediabetic, and type 2 diabetic individuals
Source: Gigascience. 2017 May 15;6(7):1–12. doi: 10.1093/gigascience/gix036 (PMC5502363; doi:10.1093/gigascience/gix036)
Supplement: GIGA-D-16-00114_Revision-2.pdf [file gix036_GIGA-D-16-00114_Revision-2.pdf]

Manuscript

Click here to download Manuscript 2017-04-18 GIGA-D-16-00114-FIN.docx

Lipidomic profiling reveals distinct differences in plasma lipid composition in healthy, prediabetic and type 2 diabetic individuals

Abstract

Background: The relationship between dyslipidemia and type 2 diabetes (T2D) has been extensively reported, but the global lipid profiles, especially in the East Asia population, associated with the development of T2D remain to be characterized.

Results: Liquid chromatography coupled to tandem mass spectrometry (LC-MS/MS) was applied to detect the global lipidome in fasting plasma of 293 Chinese individuals, including 114 T2D patients, 81 prediabetic subjects and 98 individuals with normal glucose tolerance (NGT). Both qualitative and quantitative analyses revealed a gradual change in plasma lipid features going from NGT individuals over prediabetic to T2D individuals with T2D patients exhibiting characteristics close to those in prediabetic individuals, whereas they differed significantly from individuals with NGT. We constructed and validated a random forest (RF) classifier with 28 lipidomic features that effectively discriminated T2D from NGT or prediabetes. The majority of the selected features showed significant correlations with diabetic clinical indices. Hydroxybutyrylcarnitine was positively correlated with fasting plasma glucose (FPG), 2-hour postprandial glucose (2h-PG), glycated hemoglobin (HbA1c) and insulin resistance index (HOMA-IR), and lysophosphatidylcholines such as LysoPC (18:0), LysoPC (18:1) and LysoPC (18:2) were all negatively correlated with HOMA-IR.

Conclusions: The altered plasma lipidome in Chinese T2D and prediabetic subjects suggest that lipid features may play a role in the pathogenesis of T2D and that such features may provide a basis for evaluation of risk and monitoring of disease development.

Keywords: Lipidomics, Type 2 diabetes, Prediabetes, Plasma

Background

Type 2 diabetes mellitus is a progressive and complex disease that is tightly associated with heterogeneous metabolic disorders, particularly in glucose and lipid metabolism [1]. The prevalence of prediabetes (Pre-DM),

defined by blood glucose levels between normal and diabetic levels, is increasing rapidly worldwide. Hence, characterization of abnormalities in glucose and lipid metabolism at the prediabetic state is warranted. The high prevalence of prediabetes and type 2 diabetes is rapidly increasing in highly populated countries such as China. Using criteria defined by World Health Organization (WHO) and American Diabetes Association (ADA), two national epidemiological studies reported that the prevalence of adult diabetes in China was 9.7% in 2007 and 11.6% in 2010, respectively, whereas, the prevalence of adult prediabetes ranged from 15.5% to 50.1% [2,3]. As a strong association between T2D and dysregulation of lipid metabolism is well established[1], metabolomics techniques, especially lipidomics, represent powerful tools to globally survey metabolites associated with prediabetes and T2D. Further, metabolomic analyses may provide insight into ongoing biochemical processes and identify biomarkers to predict disease risk. In a longitudinal study, comprising 2,422 normoglycemic individuals followed for 12 years, plasma levels of three branched-chain amino acids (BCAA; isoleucine, leucine, valine) and two aromatic amino acids (tyrosine and phenylalanine) exhibited highly significant associations with future development of diabetes [4]. By comparing metabolomic profiles of obese versus lean humans, Newgard et al. have further revealed a BCAA-related metabolite signature that correlates with insulin resistance, and the concomitant specific increases in C3 and C5 acyl-carnitine levels suggested increased catabolism of BCAA [5]. Rats studies based on these findings demonstrated that supplementation of a high-fat diet with BCAA caused insulin resistance despite reduced food intake and body weight [5]. Compared with lean subjects, obese nondiabetic and T2D subjects exhibit increased levels of long-chain acyl-carnitines, suggesting impairment of entry of fatty acids into mitochondria [6]. Additionally, T2D adults of comparable BMI exhibit increased levels of several short- and medium-chain acyl-carnitines, suggesting that diabetic subjects generally suffer from a complex oxidation defect [6]. The strong association between prediabetes, T2D and dysregulation of lipid metabolism is further supported by plasma profiling of 117 T2D, 64 prediabetes and 170 normal glucose tolerant (NGT) participants using targeted lipidomics [7]. This study revealed that over hundred individual lipid species, including sphingolipids, phospholipids, glycerolipids, ceramides and cholesterol esters, were tightly associated with T2D and prediabetes [7]. Additionally, T2D risk classification

models of potential value for clinical use have been developed and evaluated by Meikle and coworkers to stratify individuals with an FPG <6.1 mmol/L using lipidomics profiles with or without non-lipid risk factors [8]. Non-targeted lipidomics further represents a tool to discover metabolic regulatory networks and thereby increases our understanding of lipid metabolism in the pathophysiology of metabolic diseases including T2D [9]. Of note, it has been reported that East and South Asians develop T2D at a lower mean BMI compared to the Western population [10] and Asians have more body fat and a higher tendency to visceral adiposity for a given BMI than the Western diabetes population [10]. Hence, characterization of the lipid profile of T2D in an Asian population (represented by Chinese) could contribute to filling the gaps in understanding these interethnic differences.

Conventional data-dependent acquisition (DDA) mode of mass spectrometry (MS) has been widely used for lipidomics in most laboratories, in which the detection parameters, such as the number of precursor ions selected per cycle and dynamic exclusion to minimize repeat precursor ions, can be optimized to identify complex lipid molecules [11]. The DDA performance, however, has several inherent limitations, such as limited dynamic range, a bias toward high abundance ions and long duty cycle accompanying increase in sample complexity. The strategy of data-independent acquisition (DIA) has recently been developed to alleviate these limitations [12,13]. By collecting all fragment ions simultaneously without any preselection, DIA could thus enable in-depth analysis for both qualification and quantification of lipids with the improved detection sensitivity and analysis reproducibility. However, the DIA method is not easily applicable in lipidomics as the annotation and false-discovery rate (FDR) evaluation of mass spectral features in large complex lipid datasets require more sophisticated software and integrated reference database[14].

Here, we conducted a global lipidomics analysis of 293 Chinese individuals, including 114 T2D patients, 81 prediabetic subjects and 98 individuals with NGT, using DIA-based LC-MS/MS technology. Using commercial and in-house software for analyses of the highly complex dataset, we demonstrate that the Chinese T2D patients possessed significant lipid changes in plasma as compared with the prediabetic and NGT individuals. Further, we identified a number of lipid features, including LysoPC and acylcarnitines species that hold potential as

potential indicators in prediction of diabetic risk.

Data Description

To delineate global lipidomic profiles in Chinese prediabetic and T2D diabetic patients, fasting blood samples together with the corresponding clinical and phenotypic data were collected from 114 T2D patients, 81 prediabetic individuals and 98 individuals with normal glucose tolerance in Suzhou, Jiangsu Province, China (Additional file 1). The lipids were extracted from individual plasma samples and then injected on the Waters LC-MS/MS platform in both positive and negative mode, with pooled extraction quality control (QC) samples at certain intervals (Additional file 2). The raw datasets were subjected to nonlinear alignment and normalization by the commercial software Progenesis QI 2.0 and further analyzed by the in-house pipeline, metaX [15], to generate lipid profiles. Univariate and multivariate analyses were conducted using the R statistics software to identify and evaluate the significant lipidomic features among the groups (see Methods for details).

Analyses

The integrated workflow of this study, comprising three phases is illustrated in Fig. 1. Phase 1, collecting information of clinical specimens and acquiring mass signals from LC-MS/MS; phase 2, extracting metabolic features through the software of MS data analysis; and phase 3, identifying lipid candidates in response to the development of T2D.

Assessment of clinical characteristics and plasma lipidomic features

The clinical information including physiological and biochemical parameters of the individuals included in the cohort is summarized in Table 1. The levels of FPG, 2h-PG, HbA1c, fasting C-peptide and HOMA-IR were significantly higher in both T2D patients and prediabetic individuals than in NGT individuals, with T2D patients exhibiting higher values compared with prediabetic individuals (*Dunn's* post hoc test, $p<0.05$). T2D

patients had higher age, BMI, waist-hip ratio, systolic blood pressure (SBP), triglyceride (TG), total cholesterol (TC) and low-density lipoprotein (LDL) levels than NGT individuals (*Dunn's* post hoc test, $p<0.05$). Further, the proportion of people taking calcium channel blockers (CCBs) for hypertension was higher in T2D patients than in prediabetic and NGT individuals (Chi-square test, $p<0.05$).

We assessed both coverage and reproducibility of the non-targeted lipidomic data on our large sample cohorts. Using Progenesis QI 2.0 and metaX, the untargeted lipidomic analysis yielded 11,077 features with average coefficient of variation (CV) of 14.8% in the positive ion mode (PIM) and 923 features with average CV of 14.7% in the negative ion mode (NIM) after strict quality control (Additional file 3). Of the features in PIM, approximately 46.77% (5,181/11,077) were matched to one or more molecular species with characteristic compatible with lipids or lipid-like compounds, whereas the corresponding distribution in NIM was 36.72% (339/923). Principal component analysis (PCA) showed that all of the QC samples spiked at certain intervals clustered together, verifying an acceptable reproducibility and stability of the results (Additional file 4). In addition, 12 positive and 9 negative datasets were considered as outliers and were removed (Additional file 4A, B).

The interactions between the clinical parameters (Additional file 1) and the global lipid profiles, obtained by combining datasets from both ion modes (Additional file 4), were further evaluated by permutational multivariate analysis of variance (PERMANOVA). The lipidomic datasets were significantly associated with several plasma indices such as TG, HDL, TC, leptin, C-peptide, and HOMA-IR index, and with physiological conditions, such as gender, waist-hip ratio, BMI and age (FDR<0.05) (Additional file 5). Moreover, weak interactions were observed between the global lipid profiles and other clinical parameters such as levels of fasting insulin, FPG, LDL, SBP and CCBs treatment ($p<0.05$ and FDR>0.05).

Prediabetes and T2D-related lipidomic features

Due to the observed effects of CCB use on the lipid profiles, we performed a blocked Kruskal-Wallis test, using CCBs treatment as the blocking factor, followed by *Dunn's* post hoc test for pairwise comparisons. As shown in

Additional file 6, 1,590 features displayed significant differences between the 3 groups, including 1,395 features in PIM and 195 features in NIM (p<0.05, KW test). Of these, 790 potentially matched lipids or lipid-like compounds, including lysolipids, PC (phosphatidylcholines), carnitines, DG (diglyceride), TG and several free fatty acids. As depicted in Fig. 2, pairwise comparisons revealed that 1269 features displayed significant differences between NGT individuals and T2D patients, whereas 785 and 578 features showed significant differences between prediabetic individuals vs. NGT individuals and T2D patients, respectively (Additional file 6, p<0.05). The low number of variables distinguishing prediabetes and T2D, suggested that changes in a large fraction of the lipid features in prediabetes and T2D were shared, implying that compared with NGT, lipid profiles characterizing prediabetes and T2D are similar. Further, 117 features maintained significances after controlling the FDR by Benjamini-Hochberg multiple testing correction (Additional file 6, FDR <0.05).

To quantify the differential features among the three groups, all detected features were assessed using criteria of fold change (FC) in mass intensity $\geq$ 1.2 or $\leq$ 0.8 and variable importance of the projection (VIP) > 1.0 estimated by Partial Least Squares Discriminant Analysis (PLS-DA). Of the 1,590 differential features, 27.80% were significantly different between T2D and NGT, representing 229 and 231 features at higher and lower levels, respectively (Additional file 6). Only 7.36% of the features differed significantly between T2D patient and prediabetic individuals, representing 58 features present in higher abundance and 59 features present in lower abundance in T2D patients compared with prediabetic individuals. 42.79% (98/229) of the lipid features detected at higher levels in T2D patients than in NGT individuals overlapped with metabolites detected at higher levels in prediabetic than in NGT individuals. However, only 18.31% (39/213) of the features detected at lower levels in T2D patients compared with NGT individuals overlapped with metabolites detected at lower level in prediabetic compared with NGT individuals. These findings suggest that the pattern of changes in the levels of a subset of metabolites reflect a continuous progression from NGT to T2D via prediabetes.

T2D risk evaluation using Random Forest (RF) classifier

As the qualitative and quantitative analyses revealed significant differences in the lipid levels between the three groups and indicated a gradual change from NGT to T2D via prediabetes, we investigated if the lipid profile

could predict risk of further T2D development. To evaluate this possibility, a random forest (RF) classifier was used. Samples for T2D and NGT groups were randomly divided into two sets, with 70 T2D and 70 NGT as the training set and the rest as the validation set. As illustrated in Fig. 3A, a model containing 28 features was successfully generated. The model exhibited excellent performance on the training set with area under the Receiver Operating Characteristic (ROC) curve (AUC) of 90.23% (95% confidence interval (CI)=84.95-95.52%) (Fig. 3B), and a high validated performance of AUC of 86.24% (95% CI= 76.05−96.43%) (Fig. 3C). The T2D risk prediction model was further used to evaluate the performance in relation to distinguishing prediabetes from T2D or NGT. As depicted in Fig. 3D-3E, T2D or NGT was basically different from prediabetes with the AUC of 71.77% (95% CI= 61.95−81.58%) or of 68.08 (95% CI= 54.87−81.28%), respectively. Additionally, the risk probability (RP) for each sample was estimated and indicated a gradually increasing risk from NGT to prediabetes to T2D. Interestingly, similar increasing trends of RP were observed in subgroups of the prediabetic individuals. A low risk was estimated in subjects with high HbA1c levels of 5.7-6.4%, a slightly higher risk in subjects with isolated impaired glucose tolerance (iIGT) and a high risk was estimated in subjects with combined impaired fasting glucose and impaired glucose tolerance (IFG/IGT) (Fig. 3F). Information on selected features, such as retention times (RT), parent mass-to-charge ratio (*m/z*), matching compounds and lipid categories are presented in Additional file 7 and the relative intensity levels of the features are shown in Additional file 8 (A-AB). The relative intensities of all selected features in T2D patients were significantly different from the levels in NGT subjects (*Dunn's* post hoc test, $p < 0.05$). In prediabetic individuals, the relative intensities of these features were intermediate between T2D and NGT, and the abundance of only a fraction of the features was significantly different comparing prediabetes with T2D or NGT. Together, these results indicate that the lipid profile is regulated in a complex manner during development of prediabetes and T2D.

Statistical analysis to define the correlation of T2D-related lipid compounds and diabetic parameters

To explore the association structure between the lipidomic features and clinical diabetic parameters, we performed a general linear model (GLM) regression analysis. In total, 81.38% (1294/1590) differential features

were significantly correlated with at least one of the diabetes-related indices after adjustment for age, gender, BMI, as well as hypertension, hyperlipidemia, smoking and alcohol history. All RF selected features passing the cutoff point of $p<0.05$, and 65.89% (850/1590) met an FDR<0.05 including 27 RF selected features except *m/z*1019.7063 (ESI+, RT=1.81min) (Additional file 9). Among these 27 RF features, we observed stronger relationships between metabolites and glycemic variables (FPG, 2h-PG and HbA1c) than beta-cell function indices (fasting insulin, C-peptide and HOMA-IR), and weaker associations with age or BMI after adjusting diabetes status and other confounders (Fig. 4). For instance, *m/z 203.0533* (ESI+, RT=0.58min) showed highest positive correlations with glycemic variables (Beta= 0.625, 0.588 and 0.466 for HbA1c, FPG and 2h-PG, respectively), whereas a relatively weak negative correlation with age (Beta=-0.197) was observed (Additional file 9). No statistically significant relationship between *m/z 203.0533* (ESI+, RT=0.58min) and fasting C-peptide or insulin levels was found (Additional file 9). Further, we observed an inverse relationship between LysoPC (P-16:0) at *m/z 480.3456* (ESI+, RT=1.46min) and the 6 diabetes-related indices. The abundance of LysoPC (P-16:0) correlated strongly with beta-cell functions (Beta= -0.334, -0.308 and -0.282 for HOMA-IR, C-peptide and insulin, respectively), but did not correlate with age, gender or BMI. Conversely, TG (62:9) at *m/z* 967.8174 (ESI+, RT=7.97min) was negatively correlated with both diabetes related indexes and BMI. Among the 1590 differential features, several potential TG species, including TG (48:1) at *m/z 832.7403* (ESI+, RT=8.25min), TG (50:1) at *m/z 855.7427* (ESI+, RT=8.46min), TG (50:2) at *m/z 853.7262* (ESI+, RT=8.35min) and TG (50:3) at *m/z 851.7108* (ESI+, RT=8.25min) showed high positive correlations with both BMI and C-peptide levels ($p<0.05$, Additional file 9).

T2D-related lipid identification using DDA

The RF selected features were further identified by DDA and classified by Metabolomics Standards Initiative (MSI) according to their degree of physicochemical and/or spectral similarity to available reference lipid standards or to published data[16]. The *m/z 248.1511* (ESI+, RT=0.56min) with a reported prominent fragment ion at *m/z* 85 was annotated as hydroxybutyrylcarnitine (Additional file 10) [17]. The highest abundance of this

metabolite was detected in T2D, whereas the lowest levels were found in NGT (Additional file 8C). By comparing mass spectra of precursor ions using Progenesis QI 2.0, two RF selected negative features, namely *m/z 508.3404* (RT=1.7min) and *m/z 508.3406* (RT=1.83min) with different retention times, but almost identical *m/z* values were both annotated as LysoPC (18:0). The identity of the compound was subsequently confirmed by comparison with the authentic reference standard, with the most abundant peak at *m/z 283.2641* corresponding to the acyl anion fragments of stearic acid (Additional file 11). Further, *m/z 506.3249* (ESI-, RT=1.39min) and *m/z 504.3093* (ESI-, RT=1.12min) were annotated as LysoPC (18:1) and LysoPC (18:2) with characteristic peaks at *281.2483* and *279.2326* as fragments of oleic and linoleic acid, respectively (Additional file 12-13). We suggest that the peak with *m/z 224* present in spectra of all three compounds is the product of ketene loss of the demethylated lysoPCs (Additional file 11-13) [18,19]. The levels of the three LysoPC species were similar in NGT and prediabetes, however, the levels of all three were significantly lower in T2D patients than in NGT and prediabetic individuals (Additional file 8B, 8G, 8P, 8R).

Discussion

Prediabetes, representing a high-risk condition that precedes onset of T2D, has attracted significant attention in relation to T2D prevention and pathogenesis research. To evaluate the risk of T2D development, Morris et al. examined 70 follow-up studies, where prediabetic subjects were defined by different criteria, and estimated the progression rates per 1,000 person-years from prediabetes to T2D. The meta-analysis demonstrated T2D incidences of 35.54/1000 for HbA1c$_{6.0\text{-}6.4\%}$, 45.46/1000 for iIGT and 70.36/1000 for combined IFG/IGT[20]. The T2D risk of similar prediabetic subgroups in this study, HbA1c$_{5.7–6.4\%}$, iIGT and combined IFG/IGT , was assessed using the RF algorithm, which predicted a gradually increased T2D risk from HbA1c$_{5.7–6.4\%}$ to iIGT to combined IFG/IGT. This prediction is overall consistent with the progression rates determined in the analysis of Morris et al. [20], but we also emphasize that a thorough statistical validation using longitudinal studies of larger cohort will be required to demonstrate the clinical utility of our model in the Han Chinese population. The low risk probability predicted for prediabetic individuals diagnosed with elevated HbA1c suggests that

appropriate HbA1c criteria for Chinese prediabetic individuals should also be determined. Interestingly, Meikle's groups has developed several T2D risk classification models to evaluate their performances in relation to stratifying T2D and IGT from NGT, all with FPG <6.1 mmol/L. As reported, their combined model with common risk factors and certain plasma lipids showed a maximum AUC of 0.826 and significant gains in both mean AUC of 0.049 ($p < 0.001$) and net reclassification improvement of 10.5% ($p < 0.001$) compared with the model based solely on common risk factors such as BMI, gender and HbA1c[8]. DG and TG species are the two most frequently incorporated lipid classes in the classification models, including DG 16:0/16:0, DG 16:0/22:5, DG 16:0/22:6 and TG 14:1/16:1/18:0. As mentioned above, a significant number of lipids from these two classes showed significant differences between the three clinical groups in our study, though, with few species incorporated in the risk prediction model. Our results together with results from earlier studies indicate that plasma lipid species might reflect some of the more subtle pathological changes during diabetic development.

In the present study, the major lipidomic alterations associated with T2D were characterized by enhanced levels of acylcarnitines and decreased levels of lysophosphatidylcholines. At MSI level 2, the abundance of hydroxybutyrylcarnitine at *m/z 248.1511* (ESI+, RT=0.56min) (Additional file 8C) was found to increase from NGT to prediabetes to T2D (KW test, p=2.39E-9, FDR=1.32E-5, Additional file 6). *In vivo*, D-3-hydroxybutyrylcarnitine can be converted to D-3-hydroxybutyric acid, which is the predominant ketone body found during diabetic ketoacidosis [21–23]. Notably at MSI level 4, the abundance of hydroxybutyric acid at *m/z 103.0391* (ESI-, RT=0.61min) (Additional file 8J), was also higher in T2D patients than in both prediabetic and NGT individuals (KW test, p=2.38E-8, FDR=1.01E-5, Additional file 6) in this study. These two features also showed high positive correlations with glycemic variables and HOMA-IR (Fig. 4, Additional file 9). Increased D-3-hydroxybutyric acid levels has also been suggested as an early biomarker of insulin resistance, which may be linked to mitochondrial dysfunction and the resultant oxidative stress[21].

Both T2D and prediabetes displayed relatively higher concentrations of several short- and long-chain AcylCNs, such as L-acetylcarnitine (C2) at *m/z 203.1160n* (ESI+, RT=0.57min) (*p*=6.66E-04, FDR=0.066), tetradecanoylcarnitine (C14) at *m/z 372.3115* (ESI+, RT=0.99min) (*p*=1.46E-03, FDR=0.094), 2-hydroxyhexadecanoylcarnitine (C16OH) at *m/z 416.3371* (ESI+, RT=1.02min) (*p*=1.60E-04, FDR=0.044), and 12-hydroxy-12-octadecanoylcarnitine (C18OH) at *m/z 444.3667* (ESI+, RT=1.38min) (*p*=5.70E-04, FDR=0.066) (KW test, Additional file 6). As reported, accumulation of these incompletely oxidized lipid species, likely derived from fatty acid or amino acid metabolism, may contribute to insulin resistance[6,24]. Increased acylcarnitine concentrations in the plasma of patients with T2D and prediabetes were also reported in the German population [25].

Lysophosphatidylcholine, an important signaling molecule and fatty acid carrier, constitutes 5-20% of total plasma phospholipids [26]. The alterations in species of LysoPC have been widely studies in relation to diabetes and obesity. Significantly lower levels of several LysoPC species in patients with IGT and T2D, including LysoPC (18:2), LysoPC (18:1), LysoPC (18:0) and LysoPC (17:0), were reported in a large cross-sectional study [27]. In addition, LysoPC (18:2) and glycine were selected and validated as strong baseline predictors for the risks of developing IGT and/or T2D in a prospective analysis [27]. Finally, shotgun lipidomics and data mining approaches revealed, multiple independent associations between plasma lipidomic parameters and insulin sensitivity indices, including a negative correlation between LysoPC (22:5) and HOMA-IR[28]. It has been reported that LysoPC species could enhance glucose-dependent insulin secretion via G-protein-coupled receptor G119 both *in vivo* and *in vitro*[29].

Comparing NGT and prediabetic individuals, we observed a strongly significant reduction of LysoPC species in the Chinese T2D patients including LysoPC (18:0) at *m/z 508.3406* (ESI-, RT=1.83min) (*p*=3.01E-7, FDR=5.55E-05) and *m/z 508.3404* (ESI-, RT=1.7min) (*p*=2.93E-05, FDR=1.88E-03), LysoPC (18:1) at *m/z 506.3249* (ESI-, RT=1.39min) (*p*=7.95E-06, FDR=6.67E-04) and LysoPC (18:2) at *m/z 504.3093* (ESI-, RT=1.12min) (*p*=6.28E-06, FDR=3.41E-03), which were selected by the T2D prediction model (KW test,

Additional file 6). Also, LysoPC (22:5) at *m/z 569.3440n* (ESI+, RT=1.01min) showed a slight decrease in T2D and prediabetes (*p*=0.027, FDR=0.310), while no significant associations with HOMA-IR or other diabetes-related indices was observed. The differences suggest the presence of distinct lipid profiles characterizing T2D populations in China and Germany.

Some T2D associated features are also tightly correlated with obesity. Compared with lean control, Melissa et al. reported a reduction of several LysoPC species in both obese T2D and obese non-T2D subjects, while no differences were observed between the two obese groups[30]. In this study, 641 significant features selected by the KW test displayed significant associations with BMI, by adjusting age, gender, diabetes status, hypertension history, hyperlipidemia history, smoking history and alcohol history, with 107 of them solely correlating with BMI (*p* <0.05, Additional file 9). Notably, 7 of the RF selected features showed additional significant associations with BMI, including the positively related PS (38:1) at *m/z 800.5850* (ESI+, RT=5.20min) and negatively associated LysoPC (18:1) at *m/z 506.3249* (ESI-, RT=1.39min), TG (62:9) at *m/z* 967.8174 (ESI+, RT=7.97min) and LysoPC (18:2) at *m/z 504.3093* (ESI-, RT=1.12min) (Fig. 4, Additional file 9). Lower levels of LysoPC(18:1) have also been observed in obese subjects[31,32]. In addition, a recent review listing metabolic biomarkers of obesity and T2D, noted a large number of shared lipid biomarkers between the 2 disorders[33]. Additionally, 5 of the RF selected features showed additional significant negative associations with age, including *m/z 203.0533* (ESI+, RT=0.58 min) and LysoPC (18:2) at *m/z 504.3093* (ESI-, RT=1.12min) (Fig. 4, Additional file 9). This indicates that there are common alterations in lipid metabolism associated with T2D, obesity and aging, supporting the idea that greater BMI and aging are the 2 main risk factors for developing T2D [34–36].

We observed that CCBs treatment (n=61) for hypertension showed slight effects on plasma lipid profiles (*p*=0.0324 and FDR=0.0825) by PERMANOVA (Additional file 5), which was consistent with an earlier report[37]. No significant effects were found in relation to other medications, probably because of smaller sample sizes or limited impacts (Additional file 5). Although further validation is required to confirm and

validate drug effects, we suggest that the medications as possible interfering factors on lipid metabolism[37,38] should be carefully considered in lipidomics-based investigations. Hence, we used strict statistical analyses to control for possible effects of treatment on T2D-associated plasma lipids.

In summary, by using LC-MS/MS based untargeted lipidomics analysis, our study is the first large-scale study to explore the alterations in the plasma lipid patterns in individuals with NGT, prediabetes and T2D from East China. We describe a large number of plasma lipids providing a broad coverage of major lipid categories. We identify thousands of plasma lipids exhibiting remarkable difference in abundance between the three diagnostic groups, with a large proportion displaying similar trends in prediabetes and type 2 diabetes. Additionally, we describe stratification of predicted diabetes risk between subgroups of prediabetes based on 28 selected plasma lipids. Several of the diabetes related candidates have not previously been reported. Together, this study provides a better biological understanding of the insidious progression to diabetes from a lipid perspective. More comprehensive studies combining genomics, metabolomics, proteomics and metagenomics should be conducted to describe the detailed variations among the prediabetes subgroups and to support precise prevention and intervention steps for T2D.

Methods

Participant recruitment, sampling and grouping

433 participants submitted a written informed consent form, and were enrolled in the study from the community health service centers of Suzhou Center for Disease Prevention and Control (CDC). All participants underwent a two-steps enrollment process. At the first visit, all participants were subjected to physical examinations including height, weight, blood pressure, waist and hip circumference and completed a face-to-face questionnaire on demographics, medication history, family health history and other lifestyle factors via well-trained local staffs. The study only enrolled the participants who met the following criteria based on questionnaire, including 1) age 40 or older; 2) free of cardiovascular disease, severe renal disease, cancer, type 1 or monogenic diabetes and other autoimmune diseases, as determined by self-reporting; and 3) no antibiotic use during the past 2 months. Approximately 81.7% of the participants in the cohort (354 out of 433) meeting the above criteria were admitted to a blood screening tests for diabetes according to the 2011 WHO criteria[39]. The qualified participants without a self-reported history of type 2 diabetes were given a 2-hour 75 g oral glucose tolerance test (OGTT). Participants with fasting or postprandial blood glucose levels above the diagnostic cut-off point were asked to repeat the test on the next day. Blood medical tests were performed by a Nanjing Kingmed Center for Clinical Laboratory, which included FPG, insulin, C-peptide, HbA1c, leptin, adiponectin, and blood lipid levels in addition to routine blood tests. Fasting plasma was prepared within 1 hour after blood withdrawal by centrifuged at 1,600 g for 15 minutes. The upper layers were carefully collected to avoid disturbing the buffy coat cells. The isolated plasma samples were stored at -80°C and transported on dry ice to BGI-Shenzhen.

Finally, a total of 293 subjects were divided into the 3 diagnostic groups, namely the normal glucose tolerance group (NGT, n=98), the prediabetes group (Pre-DM, n=81) and the type 2 diabetes group (T2D, n=114; including 77 newly diagnosed and 37 self-reported patients). The Pre-DM samples were further classified into 4 subgroups: a) raised $HbA1c_{5.7\text{-}6.4\%}$ (defined by the WHO-HbA1c criteria only; n=15); b) isolated IFG (defined by an FPG level of 6.1-7.0 mmol/l and a normal 2h-PG level; n=7); c) isolated IGT (defined by a normal FPG

level and a 2h-PG level of 7.8–11.0 mmol/l; n=35); and d) combined IFG/IGT (defined by an FPG of 6.1-7.0 mmol/l and a 2h-PG level of 7.8–11.0 mmol/l; n=24). The study was approved by the Institutional Review Board of BGI-Shenzhen and the ethical review committee of Suzhou CDC.

Lipidome data processing and analysis (Lipidomics)

1. Lipid preparation and extraction

The collected plasma samples were thawed on ice, and lipids were extracted with isopropanol (IPA) using a previously described method[12]. Briefly, 40 μL of plasma was extracted with 120 μL of precooled IPA, vortexed for 1 min, and incubated at room temperature for 10 min; the extraction mixture was then stored overnight at -20°C. After centrifugation at 4,000 g for 20 min, the supernatants were transferred into new 96-well plates and diluted to 1:10 with IPA/acetonitrile (ACN)/H_2O (2:1:1, v:v:v). The samples were stored at -80°C prior to the LC-MS analysis. In addition, pooled plasma samples were also prepared by combining 10 μL of each extraction mixture.

2. UPLC-MS method for lipidomics

Samples were analyzed with an ACQUITY UPLC (Waters, Manchester, USA) connected to a XEVO-G2XS QTOF mass spectrometer (Waters) with electrospray ionization (ESI). The lipids were separated using an Acquity UPLC CSH C18 column (2.1×100 mm, 1.7 μm, Waters) with a gradient mobile phase comprised of 10 mM ammonium formate with 0.1% formic acid in acetonitrile/water (A, 60:40, v/v) and 10 mM ammonium formate with 0.1% formic acid in isopropanol/acetonitrile (B, 90:10, v/v). Before the large-scale study, pilot experiments including 10min, 15min and 20min elution periods were conducted to evaluate the potential effects of mobile phase composition and flow rate on lipids retention time. Both abundant lipid precursors ions and fragments were separated in the same order with similar peak shapes and ion intensities in PIM (Additional file 14A-C, Additional file 15A-C). Furthermore, the mixed QC samples with the 10min elution period also showed similar base peak intensities (BPI) of precursors and fragments with the test sample (Additional file 14D,

Additional file 15D). Considering the large sample size of this study, we used the accelerated elution profile of 10 min described in the following sections. The mobile phase was delivered at a flow rate of 0.4 mL/min. The column was initially eluted with 40% B, followed by a linear gradient to 43% B over 2 min, and then the percentage of B was increased to 50% within 0.1 min. Over the next 3.9 min, the gradient was further ramped to 54% B, and the amount of B was then increased to 70% in 0.1 min. In the final part of the gradient, the amount of B was increased to 99% over 1.9 min. Finally, solution B was returned to 40% in 0.1 min, and the column was equilibrated for 1.9 min before the next injection. The injection volume was 10 μL. Lipids were detected with a XEVO-G2XS QTOF mass spectrometer in positive and negative mode, which was operated in MS^E mode from *m/z 50-2,000*, with an acquisition time of 1 s per scan. The source temperature was set at 120°C. The desolvation temperature and gas flow were 600°C and 800 L/h, respectively, and nitrogen was used as the flow gas. The capillary and cone voltages were 2.0 kV (+) / 1.5 kV (-) and 30 V, respectively. Leucine encephalin (molecular weight (MW) = 555.62; 200 pg/μL in 1:1 ACN:H_2O) was used as a lock mass for accurate mass measurements, and 0.5 mM sodium formate solution was used for calibration. The samples were randomly ordered, and 10 QC samples were initially injected to condition the column. One QC sample was injected and analyzed every 10 samples to investigate the repeatability of the data[41].

3. Acquisition of the high quality non-targeted metabolic profile and Metabolite identification

The raw MS/MS datasets were generated on the Waters XEVO-G2XS QTOF instrument and processed using commercial software Progenesis QI 2.0 (Nonlinear Dynamics, Newcastle, UK), consisting of raw data import, selection of possible adducts, peak set alignment, peak detection, deconvolution, dataset filtering, noise reduction, compound identification and normalization with sum method. The analysis parameters used were as follows: 1) possible adducts of $[M+H]^+$, $[M+H-H2O]^+$, $[M+Na]^+$ and $[M+K]^+$ for ESI+ and $[M-H]^-$ for ESI-, 2) the retention time of 0.5-9min, 3) the peak width of 1-30s, 4) 10 ppm mass tolerance for the precursors, 5) 10ppm fragment mass tolerance for theoretical fragmentation searching to improve the confidence in compound identification. The normalized peak data was further preprocessed by an in-house software metaX [42]. Those features that were detected in less than 50% of QC samples or 80% of biological samples were

removed, the remaining peaks with missing values were imputed with k-NN (k-Nearest Neighbor) algorithm to further improve the data quality. PCA was performed for outlier detection and batch effects evaluation using the pre-processed dataset. QC-RLSC (quality control–based robust LOESS signal correction) was fitted to the QC data with respect to the order of injection to minimize signal intensity drift over time. In addition, the relative standard deviations (RSDs) of the metabolic features were calculated across all QC samples. The features with RSDs >30% were then removed. The high-resolution LC-MS/MS features were identified using Progenesis QI 2.0 by searching in the public databases including Human Metabolome Database (HMDB, version 3.6, http://www.hmdb.ca/), LIPID MAPS Structure Database (LMSD, http://www.lipidmaps.org/) and LipidBlast [43] with the mentioned parameters. To obtain reliable identification of the high quality features, the identification of matching lipids were filtered by defined retention time ranges in terms of application note of CSHC$_{18}$ UPLC System provided by Waters Corporation[44]. The retention times were 0.5-4min for lysophospolipids including LysoPC, lysophosphatidylethanolamine (LysoPE), lysophosphatidylglycerol (LysoPG), lysophosphatidylserine (LysoPS), lysophosphatidic acid (LysoPA) and lysophosphatidylinositol (LysoPI) species, 3-8.1min for sphingolipids, including sphingomyelin (SM), ceramide (Cer) and lactosylceramide (LacCer), glucosylceramide (GluCer) and galactosylceramide (GalCer) species, 4-7.8min for PC, PE, PG, PS, PA and PI species, 7.8-9.5min for DG, TG and cholesteryl ester (CE) species in PIM (Additional file 14D, Additional file 15D); 0.5-4min for lysophospolipids and FFA species and 4-9min for phospholipids (PC, PE, PG, PS, PA and PI) and sphingolipid species in NIM. The metabolites that could be matched to LMSD, LipidBlast or the aliphatic compounds of HMDB at the Molecular Framework level were considered lipids and lipid-like features. DDA, which covered the desired mass scan range of interest, was performed to further aid in identifying the metabolites. Pure authentic standard of LysoPC (18:0) (Product Code: 855775P) from Avanti Polar Lipids Inc (Alabaster, AL) was used to validate the lipids by comparing their MS/MS spectra and retention time on Waters XEVO-G2XS QTOF instrument. The reported features were classified into Metabolomics Standards Initiative (MSI) levels according to the reported guidelines[16].

4. Data analysis

4.1 Univariate analysis: Kruskal-Wallis testing and fold change analysis

Blocked Kruskal-Wallis tests were conducted to detect differences in metabolite concentrations among the 3 diagnostic groups after controlling for the potential confounding effects of CCB drugs. The analysis was performed using the tools implemented in the COIN software package (coin 1.1-2 in R 3.2.5). The *p* value was adjusted for multiple tests using an FDR (Benjamini-Hochberg). *Dunn's* post hoc tests followed by pairwise comparisons were performed; "=" indicates no significant difference and ">" indicates *p* values <0.05. Fold changes were calculated by comparing the mean concentrations of each feature between groups.

4.2 Multivariate analysis

To improve the performance of the subsequent statistical analyses, all features were normalized to the range of [0, 1] to stabilize the variance using a modified range-scaling method with the following formula [45]:

$$\tilde{x}_{ij} = \frac{x_{ij} - x_{i_{min}}}{(x_{i_{max}} - x_{i_{min}})}$$

where $\tilde{x}_{ij}$ indicates the scaled value for *i*-th variable (compound) in the *j*-th sample (which is valued by x_{ij},) and $x_{i_{max}}$ and $x_{i_{min}}$ represent the maximum and minimum values for the *i*-th variable among the samples, respectively.

PERMANOVA for the influence of clinical and lifestyle factors

Permutational multivariate analysis of variance (PERMANOVA) was performed on the normalized lipid metabolite profiles and phenotypes with Bray-Curtis distance (adonis function, vegan package in R 3.2.5). The number of permutations was 9,999. And the *p* value was corrected for multiple tests using an FDR (Benjamini-Hochberg) cut-off of 0.05.

PLS-DA analysis

A supervised partial least-squares discriminant analysis (PLS-DA) was conducted through metaX[42] to discriminate the different variables between groups. The variable importance of the projection (VIP) value was calculated. A VIP cut-off value of 1.0 was used to select important features.

Random forest (ROC/AUC) analysis

The RF classifier (randomForest 4.6-12 in R 3.2.5) was trained on 140 randomly selected subjects (70 NGT and 70 T2D) from the 273 samples and then tested on the remaining subjects. All of the features were supplied to the classifier. The analysis was conducted with 5 repetitions of the 10-fold cross-validation, using cross-validation error curves to selected features as described by Feng et al. [46]. The risk probability (RP) of T2D for each subject was computed by the selected features and a ROC curve was drawn, for which the AUC was calculated (pROC1.8 in R 3.2.5). The selection frequencies of features were listed to measure the importance of the variables, with a higher frequency indicating the greater importance of a given metabolite for classifying T2D and NGT. The RF model was further tested on the validation sets.

General linear model regression analysis

Regression analysis using general linear model was conducted to investigate the associations between metabolites and multiple clinical phenotypes. In model applied with metabolites and diabetes-related indexes including FPG, 2h-PG, HbA1c, fasting Insulin, C-peptide and HOMA-IR, the confounding factors including age, BMI, gender and CCBs use were adjusted. Model applied with metabolites and age were adjusted for diabetes status, BMI, gender and CCBs use. Model applied with metabolites and BMI were adjusted for diabetes status, age, gender and CCBs use. For each feature, the standardized regression coefficient (Beta) and two-tailed *p* value for coefficient were calculated (glm in R 3.2.5). The *p* value of less than 0.05 was regarded as significant.

Abbreviations

T2D	type 2 diabetes
Prediabetes	Pre-DM
NGT	normal glucose tolerant
LysoPC	lysophosphatidylcholine
HbA1c	glycated hemoglobin
(i)IGT	(isolated) impaired glucose tolerance
(i)IFG	(isolated) impaired fasting glucose
WHO	World Health Organization
ADA	American Diabetes Association
2h-PG	2-hour postprandial glucose
FPG	fasting plasma glucose

LC-MS/MS	liquid chromatography-tandem mass spectrometry
FFA	free fatty acid
AcylCNs	acylcarnitines
DG	diglycerides
DDA	data-dependent analysis
DIA	data-independent acquisition
FDR	false discovery rate
BMI	body mass index
QC	quality control
KW test	Kruskal-Wallis test
HOMA-IR	insulin resistance index
TG	triglyceride
TC	total cholesterol
LDL	low-density lipoprotein
SBP	systolic blood pressure
DBP	diastolic blood pressure
CCBs	calcium channel blockers
PCA	Principal Components Analysis
CV	coefficient of variation
PIM	positive ion mode
NIM	negative ion mode
PERMANOVA	Permutational multivariate analysis of variance
HDL	high-density lipoprotein
FC	fold change
PC	phosphatidylcholine
VIP	Variable Importance of the Projection
PLS-DA	Partial Least Squares Discriminant Analysis
RF	random forest
ROC	Receiver Operating Characteristic
AUC	area under the curve
CI	confidence Interval
RT	retention times
RP	risk probability
ESI	electrospray ionization
GLM	general linear model
MSI	Metabolomics Standards Initiative
OGTT	oral glucose tolerance test
IPA	isopropanol
ACN	acetonitrile
UPLC-MS	ultra-performance liquid chromatography-tandem mass spectrometry
MW	molecular weight
k-NN	k-Nearest Neighbor
QC-RLSC	quality control–based robust LOESS signal correction
RSDs	relative standard deviations
LysoPE	lysophosphatidylethanolamine
LysoPG	lysophosphatidylglycerol
LysoPS	lysophosphatidylserine
LysoPA	lysophosphatidic acid

LysoPI	lysophosphatidylinositol
SM	sphingomyelin
Cer	ceramide
LacCer	lactosylceramide
GluCer	glucosylceramide
GalCer	galactosylceramide
PE	phosphatidylethanolamine
PG	phosphatidylglycerol
PS	phosphatidylserine
PA	phosphatidic acid
PI	phosphatidylinositol
CE	cholesteryl ester

Declarations

Availability of supporting data and materials

The raw mass spectrometry datasets have been deposited in the MetaboLights open access data repository [MTBLS352]. The commercial software Progenesis QI 2.0 (Nonlinear Dynamics, Newcastle, UK, http://www.nonlinear.com) was purchased from Waters Corporation. The in-house software metaX was available for Bioconductor (https://bioconductor.riken.jp/packages/3.2/bioc/html/metaX.html). The supplemental data and custom scripts were hosted in the *GigaScience* GigaDB repository (ref).

Competing financial interests

The authors declare no competing interests.

Author contributions

J.L., S.L. and C.N. conceived and directed the project. Y.L, G.Z., J.C., X. B, Y.H., Y.G., J.Z and C.N oversaw the sample collection and provided phenotypic information. J.L. routinely managed the project at BGI-Shenzhen. G.H., J.Z. and Y.F. contributed to the experiment. H.Z., C.F., Y.F., B.W, H.R., F.Y., Z.Y., J.W and Y.P. performed the bioinformatic analyses, and prepared figures and texts for manuscript. H.Z., J.L and S.L. wrote the manuscript. L.M. K.K. and J.L. performed substantial revision of the manuscript. H.Z., C.F., Y.F.,

B.W, G.H, H.R., Z.Y, H.X, Z.J, J.L., K.K. and S.L. participated in discussions. All authors contributed to the revision of the manuscript.

Acknowledgments

We thank all the volunteers participating in this study, the staffs from Suzhou CDC and its affiliated organizations for collecting samples and physical and daily phenotypes. This study was supported by the Shenzhen Municipal Government of China (JSGG20160229172752028, JSGG20140702161403250, CXB201108250098A, DRC-SZ [2015]162) and Suzhou Biobank (SS201111). And we gratefully acknowledge colleagues at BGI-Shenzhen for lipid extraction, LC/MS analysis and helpful discussions. We also thank Professor Xianlin Han from Sanford Burnham Prebys Medical Discovery Institute and Professor Juergen Graessler from Dresden University of Technology for their useful suggestion on lipid identification.

References

1. Stumvoll M, Goldstein BJ, van Haeften TW. Type 2 diabetes: principles of pathogenesis and therapy. Lancet. 2010;365:1333–46.

2. Yang W, Lu J, Weng J, Jia W, Ji L, Xiao J, et al. Prevalence of diabetes among men and women in China. N. Engl. J. Med. 2010;362:1090–101.

3. Xu Y, Wang L, He J, Bi Y, Li M, Wang T, et al. Prevalence and control of diabetes in Chinese adults. Jama. 2013;310:948–59.

4. Wang TJ, Larson MG, Vasan RS, Cheng S, Rhee EP, McCabe E, et al. Metabolite profiles and the risk of developing diabetes. Nat. Med. 2011;17:448–53.

5. Newgard CB, An J, Bain JR, Muehlbauer MJ, Stevens RD, Lien LF, et al. A Branched-Chain Amino Acid-Related Metabolic Signature that Differentiates Obese and Lean Humans and Contributes to Insulin Resistance. Cell Metab. 2009;9:311–26.

6. Mihalik SJ, Goodpaster BH, Kelley DE, Chace DH, Vockley J, Toledo FGS, et al. Increased levels of plasma acylcarnitines in obesity and type 2 diabetes and identification of a marker of glucolipotoxicity. Obesity (Silver Spring). 2010;18:1695–700.

7. Meikle PJ, Wong G, Barlow CK, Weir JM, Greeve MA, MacIntosh GL, et al. Plasma Lipid Profiling Shows Similar Associations with Prediabetes and Type 2 Diabetes. PLoS One. 2013;8.

8. Wong G, Barlow CK, Weir JM, Jowett JBM, Magliano DJ, Zimmet P, et al. Inclusion of Plasma Lipid Species Improves Classification of Individuals at Risk of Type 2 Diabetes. PLoS One. 2013;8.

9. Han X. Lipidomics for studying metabolism. Nat. Rev. Endocrinol. 2016;12:668–79.

10. Ma RCW, Chan JCN. Type 2 diabetes in East Asians : similarities and differences with populations in Europe and the United States. 2013;1281:64–91.

11. Köfeler HC, Fauland A, Rechberger GN, Trötzmüller M. Mass spectrometry based lipidomics: an overview of technological platforms. Metabolites. 2012;2:19–38.

12. Mapstone M, Cheema AK, Fiandaca MS, Zhong X, Mhyre TR, MacArthur LH, et al. Plasma phospholipids identify antecedent memory impairment in older adults. Nat. Med. 2014;20:415–8.

13. Cai X, Perttula K, Pajouh SK, Hubbard A, Nomura DK, Rappaport SM. Untargeted lipidomic profiling of human plasma reveals differences due to race, gender and smoking status. Metabolomics Open Access. OMICS International; 2014;2014.

14. Han X. Lipidomics: Comprehensive Mass Spectrometry of Lipids. Wiley. Wiley; 2016.

15. Wen B, Mei Z, Zeng C, Liu S. metaX : a flexible and comprehensive software for processing metabolomics data. BMC Bioinformatics; 2017;

16. Sumner LW, Amberg A, Barrett D, Beale MH, Beger R, Daykin CA, et al. Proposed minimum reporting standards for chemical analysis. Metabolomics. 2007;3:211–21.

17. Chace DH, Hillman SL, Van Hove JLK, Naylor EW. Rapid diagnosis of MCAD deficiency: Quantitative analysis of octanoylcarnitine and other acylcarnitines in newborn blood spots by tandem mass spectrometry. Clin. Chem. 1997;43:2106–13.

18. Taguchi R, Ishikawa M. Precise and global identification of phospholipid molecular species by an Orbitrap mass spectrometer and automated search engine Lipid Search. J. Chromatogr. A. 2010;1217:4229–39.

19. Ekroos K, Ejsing CS, Bahr U, Karas M, Simons K, Shevchenko A. Charting molecular composition of phosphatidylcholines by fatty acid scanning and ion trap MS3 fragmentation. J. Lipid Res. 2003;44:2181–92.

20. Morris DH, Khunti K, Achana F, Srinivasan B, Gray LJ, Davies MJ, et al. Progression rates from HbA1c 6.0-6.4% and other prediabetes definitions to type 2 diabetes: A meta-analysis. Diabetologia. 2013;56:1489–93.

21. An J, Muoio DM, Shiota M, Fujimoto Y, Cline GW, Shulman GI, et al. Hepatic expression of malonyl-CoA decarboxylase reverses muscle, liver and whole-animal insulin resistance. Nat. Med. 2004;10:268–74.

22. Hack A, Busch V, Pascher B, Busch R, Bieger I, Gempel K, et al. Monitoring of ketogenic diet for carnitine metabolites by subcutaneous microdialysis. Pediatr. Res. 2006;60:93–6.

23. Soeters MR, Serlie MJ, Sauerwein HP, Duran M, Ruiter JP, Kulik W, et al. Characterization of D-3-hydroxybutyrylcarnitine (ketocarnitine): An identified ketosis-induced metabolite. Metabolism. 2012;61:966–73.

24. Adams SH, Hoppel CL, Lok KH, Zhao L, Wong SW, Minkler PE, et al. Plasma Acylcarnitine Profiles Suggest Incomplete Long-Chain Fatty Acid b-Oxidation and Altered Tricarboxylic Acid Cycle Activity in Type 2 Diabetic African-American Women 1–3. J. Nutr. Genomics Proteomics, Metabolomics J. Nutr. 2009;139:1073–81.

25. Mai M, Tönjes A, Kovacs P, Stumvoll M, Fiedler GM, Leichtle AB. Serum levels of acylcarnitines are altered in prediabetic conditions. PLoS One. 2013;8.

26. Virtanen J a, Cheng KH, Somerharju P. Phospholipid composition of the mammalian red cell membrane can be rationalized by a superlattice model. Proc. Natl. Acad. Sci. U. S. A. 1998;95:4964–9.

27. Wang-Sattler R, Yu Z, Herder C, Messias AC, Floegel A, He Y, et al. Novel biomarkers for pre-diabetes identified by metabolomics. Mol. Syst. Biol. 2012;8.

28. Kopprasch S, Dheban S, Schuhmann K, Xu A, Schulte KM, Simeonovic CJ, et al. Detection of independent associations of plasma lipidomic parameters with insulin sensitivity indices using data mining methodology. PLoS One. 2016;11.

29. Soga T, Ohishi T, Matsui T, Saito T, Matsumoto M, Takasaki J, et al. Lysophosphatidylcholine enhances glucose-dependent insulin secretion via an orphan G-protein-coupled receptor. Biochem. Biophys. Res. Commun. 2005;326:744–51.

30. Barber MN, Risis S, Yang C, Meikle PJ, Staples M, Febbraio MA, et al. Plasma lysophosphatidylcholine levels are reduced in obesity and type 2 diabetes. PLoS One. 2012;7.

31. Heimerl S, Fischer M, Baessler A, Liebisch G, Sigruener A, Wallner S, et al. Alterations of plasma lysophosphatidylcholine species in obesity and weight loss. PLoS One. 2014;9.

32. Kim JY, Park JY, Kim OY, Ham BM, Kim HJ, Kwon DY, et al. Metabolic profiling of plasma in overweight/obese and lean men using ultra performance liquid chromatography and Q-TOF Mass spectrometry (UPLC-Q-TOF MS). J. Proteome Res. 2010;9:4368–75.

33. Park S, Sadanala KC, Kim E-K. A Metabolomic Approach to Understanding the Metabolic Link between Obesity and Diabetes. Mol. Cells. 2015;38:587–96.

34. Kahn SE, Hull RL, Utzschneider KM. Mechanisms linking obesity to insulin resistance and type 2 diabetes. Nature. 2006;444:840–6.

35. Sue Kirkman M, Briscoe VJ, Clark N, Florez H, Haas LB, Halter JB, et al. Diabetes in older adults: A consensus report. J. Am. Geriatr. Soc. 2012;60:2242–56.

36. Barzilai N, Huffman DM, Muzumdar RH, Bartke A. The critical role of metabolic pathways in aging. Diabetes. 2012;61:1315–22.

37. Kaur M, Kaur K, Bedi GK, Sidhu GS, Sikand R. Effect of Felodipine on the Serum Lipid Profile of Patients With Hypertension. 2000;15:63–7.

38. Zhang Y, Hu C, Hong J, Zeng J, Lai S, Lv A, et al. Lipid profiling reveals different therapeutic effects of metformin and glipizide in patients with type 2 diabetes and coronary artery disease. Diabetes Care. 2014;37:2804–12.

39. Report A, Consultation WHO. Use of glycated haemoglobin (HbA1c) in the diagnosis of diabetes mellitus. Diabetes Res. Clin. Pract. 2011;93:299–309.

40. Sarafian MH, Gaudin M, Lewis MR, Martin FP, Holmes E, Nicholson JK, et al. Objective set of criteria for optimization of sample preparation procedures for ultra-high throughput untargeted blood plasma lipid profiling by ultra performance liquid chromatography-mass spectrometry. Anal. Chem. 2014;86:5766–74.

41. Want EJ, Wilson ID, Gika H, Theodoridis G, Plumb RS, Shockcor J, et al. Global metabolic profiling procedures for urine using UPLC-MS. Nat. Protoc. 2010;5:1005–18.

42. Wen B, Mei Z, Broadhurst DI, Zeng C, Liu S. metaX: a flexible and comprehensive software for processing metabolomics data. Manuscr. under Submitt. 2016;Unpublished.

43. Kind T, Liu KH, Lee do Y, DeFelice B, Meissen JK, Fiehn O. LipidBlast in silico tandem mass spectrometry database for lipid identification. Nat Methods. 2013;10:755–8.

44. Isaac G, Mcdonald S, Astarita G. Lipid Separtion using UPLC with Charged Surface Hybrid Technology. Waters Corp. Milford, MA, USA. 2011;1–8.

45. van den Berg R a, Hoefsloot HCJ, Westerhuis J a, Smilde AK, van der Werf MJ. Centering, scaling, and transformations: improving the biological information content of metabolomics data. BMC Genomics. 2006;7:142.

46. Feng Q, Liang S, Jia H, Stadlmayr A, Tang L, Lan Z, et al. Gut microbiome development along the colorectal adenoma-carcinoma sequence. Nat. Commun. 2015;6:6528.

47. Chambers E, Wagrowski-Diehl DM, Lu Z, Mazzeo JR. Systematic and comprehensive strategy for reducing matrix effects in LC/MS/MS analyses. J. Chromatogr. B Anal. Technol. Biomed. Life Sci. 2007;852:22–34.

48. Soltwisch J, Kettling H, Vens-Cappell S, Wiegelmann M, Muthing J, Dreisewerd K. Mass spectrometry imaging with laser-induced postionization. Science (80-.). 2015;348:211–5.

49. Hankin JA, Barkley RM, Zemski-Berry K, Deng Y, Murphy RC. Mass Spectrometric Collisional Activation and Product Ion Mobility of Human Serum Neutral Lipid Extracts. Anal. Chem. 2016;88:6274–82.

50. Domingues P, Amado FML, Santana-Marques MGO, Ferrer-Correia AJ. Constant neutral loss scanning for the characterization of glycerol phosphatidylcholine phospholipids. J. Am. Soc. Mass Spectrom. 1998;9:1189–95.

51. Murphy RC, Axelsen PH. Mass spectrometric analysis of long-chain lipids. Mass Spectrom. Rev. 2011;30:579–99.

Tables and captions

Table 1 Baseline characteristics in three groups of the study

Variables	T2D (n =114)	Pre-DM [b] (n = 81)	NGT (n=98)	*p*-value[1]	T2D vs. Pre-DM[3]	T2D vs. NGT[3]	Pre-DM vs. NGT[3]
Gender (Female, n (%))	68 (59.65%)	40 (49.38%)	66 (67.35%)	0.0513			
Smoking, n (%)	18 (15.80%)	24 (29.63%)	17 (17.35%)	0.068			
Hypertension, n (%)	52 (45.61%)	38 (46.91%)	21 (21.43%)	0.0002			
CCBs use [a], n (%)	29 (25.43%)	20 (24.69%)	12 (12.24%)	0.0372			
Alcohol Drinking, n (%)	12 (10.53%)	19 (23.46%)	12 (12.24%)	0.0769			
				***p*-value[2]**			
Age, year	65.11 ± 8.77	61.99 ± 8.48	59.11 ± 9.15	4.52E-06	0.0356	4.81E-06	0.026
BMI	25.25 ± 3.14	25.23 ± 3.13	24.23 ± 3.26	0.0425	0.9965	0.0569	0.0569
waist /hip Ratio	0.92 ± 0.06	0.91 ± 0.06	0.89 ± 0.06	0.0166	0.6237	0.0291	0.0846
FPG, mmol/l	7.87 ± 1.99	5.91 ± 0.62	5.34 ± 0.36	2.20E-16	1.47E-13	3.49E-37	4.30E-06
2h-PG, mmol/l	15.1 ± 3.76	8.21 ± 1.63	6.01 ± 1.01	2.20E-16	3.23E-13	6.41E-41	1.30E-08
HbA1c, %	7.51 ± 2.06	5.51 ± 0.54	5.04 ± 0.42	2.20E-16	4.87E-15	5.19E-38	1.64E-05
Insulin, ulU/ml	8.85 ± 3.51	8.27 ± 3.75	7.39 ± 2.93	0.0017	0.2406	0.0004	0.0263
C-peptide, ng/ml	2.31 ± 0.99	2.07 ± 0.85	1.75 ± 0.69	4.55E-05	0.1524	1.70E-05	0.0079
HOMA-IR	3.13 ± 1.57	2.17 ± 1.03	1.76 ± 0.74	2.20E-16	3.64E-07	1.80E-21	0.0001
SBP, mm Hg	136.57 ± 24.26	130.91 ± 15.73	123.28 ± 16.81	3.69E-08	0.0129	9.75E-09	0.0036
DBP, mm Hg	79.73 ± 12.21	80.37 ± 8.12	77.35 ± 9.33	0.0345	0.6724	0.0653	0.0653
TG, mmol/l	2.04 ± 1.55	1.92 ± 1.19	1.55 ± 0.91	0.0051	0.6202	0.0149	0.0131
CHO, mmol/l	5.43 ± 1.39	5.22 ± 1.18	5.17 ± 1.43	0.2418	0.2083	0.2083	0.9262
LDL, mmol/l	3.86 ± 3.36	3.17 ± 2.00	2.56 ± 1.21	0.0014	0.2047	0.0005	0.0386
HDL, mmol/l	1.16 ± 0.35	1.14 ± 0.37	1.23 ± 0.32	0.2891	0.2225	0.6406	0.2029
Leptin, ng/ml	5.09 ± 1.91	4.29 ± 2.12	4.56 ± 1.67	0.0066	0.0013	0.0281	0.2107
GAD-Ab, lU/ml	13.66 ± 14.61	14.05 ± 13.14	12.92±17.45	0.066	0.3562	0.3173	0.1236
Hs-CRP, mg/l	2.62 ± 2.41	2.17 ± 1.81	2.16 ± 1.84	0.4485	0.7873	0.7873	0.7873
Adiponectin, ng/ml	37.41 ± 13.53	37.91 ± 16.57	39.2 ± 13.18	0.5321	0.6446	0.5684	0.5684

Values are given as mean ± SD or number of individuals (%).
a, CCBs, Calcium channel blockers
b, The Pre-DM (prediabetes) group consisted of 7 iIFG, 35 iIGT, 24 combined IFG/IGT and 15 rasied HbA1c
1, *p*-value of Chi-square test
2, *p*-value of Kruskal-Wallis test
3, *p*-value of *Dunn's* post-hoc test

Figures and captions

Figure 1 Flowchart for participant recruitment and data processing

The recruitment of participants was based on the 2011 WHO criteria for diabetes and prediabetes diagnoses. Blood and clinical data were acquired from 293 qualifying subjects, and untargeted lipidomics LC-MS/MS analysis was performed. The raw data were preprocessed with Progenesis QI 2.0 to extract metabolic features. Unqualified variables and samples were detected and discarded using the BGI in-house program metaX[42]. Several types of statistical analyses such as rank sum tests, fold change analysis, PLS-DA and random forest analysis were performed to identify metabolites that differed significantly between the diagnostic groups. The lipid compounds selected by the RF classifiers were identified by matching their accurate masses and MS^E ion spectral fragmentation patterns to those in the database. Data-dependent analysis (DDA) was applied to improve the resolving power for identification. See the Methods for more details.

Figure 2 Venn diagram of significant metabolites from the 3 pairwise comparisons

Venn diagram depicting the number of significant metabolic features from 3 pairwise comparisons (the direction of change was ignored, $p<0.05$, *Dunn's* post hoc test).

Figure 3 Random forest classification based on untargeted lipidomic profiling

(A) Distribution of 5 trials of 10-fold cross-validation error in RF classifiers. The model was trained using relative intensity of the detected features from both PIM and NIM in the training set of NGT and T2D (n =70 and 70). The black solid curve indicates average of the five trials (dash lines). The pink line marks the number of selected features in the optimal set. (B) Receiver Operating Characteristic (ROC) curve and area under the ROC curve (AUC) for the training set. (C-E) ROC and AUC for validation set with NGT and T2D (n=21 and 36), prediabetes and T2D (n=76 and 36), NGT and prediabetes (n= 21 and 76), respectively. (F) Box-and-whisker plot presents the risk probability of developing T2D among the validated NGT (n=21),

subgroups of prediabetes including HbA1c $_{5.7\text{-}6.4\%}$ (n=15) to iIGT (n=32) to combined IFG/IGT (n=23), and T2D (n=36) according to the RF model.

Figure 4 Heatmap of association between clinical parameters and 28 RF selected features

Hierarchically clustered heatmap of standardized regression coefficient (Beta) of glm analysis showing the correlations between the relative abundances of the 28 significant metabolites and the phenotypes.

Red indicates positive correlations and blue indicates negative correlations. The asterisk (*) denotes an FDR of <0.05 for each regression correlation. The cross (+) denotes a *p* value of <0.05 and an FDR of **>** 0.05 and the space denotes a *p* value of ≥0.05.

Additional file 1 Phenotypic and clinical information for 293 enrolled subjects

Additional file 2 Batch numbers and run orders for biological samples and QCs

Additional file 3 Detailed list of total detected plasma features

Additional file 4 Principal components analysis of plasma lipid profiling from biological samples and QCs

Principal components analysis (PCA) was performed on all samples to identify run outliers and check for possible batch effects in both positive (A) and negative modes (B). The colors represent the different sample classes: green for NGT, blue for prediabetes (Pre-DM), red for T2D, orange for QC and black for outlier.

Additional file 5 PERMANOVA of the influence of clinical records or life habits on lipid profile

Additional file 6 Detailed list of significant features among three groups

Additional file 7 Detailed list of 28 metabolic features selected by random forest classifier

Additional file 8 Box plot displays the relative intensity levels of 28 selected diabetic-related features in the normal glucose tolerance (NGT), prediabetes (Pre-DM) and type 2 diabetes (T2D)

The features are presented in an order of decreasing importance according to the selection frequencies in RF model. One asterisk (*) denotes *p*<0.05, two denote *p*<0.01 and three denote *p* <0.001 (*Dunn's* post hoc test).

Additional file 9 A generalized linear model (GLM) analysis on 1590 significant features and clinical phenotypes

Additional file 10 MS/MS spectra of *m/z 248.1511* (ESI+, RT=0.56min) and its inferred chemical structure

Product ion spectra obtained from MS/MS of *m/z 248.1511* $[M+H]^+$ in the positive ion mode. Each arrow indicates a possible site of fragmentation, including a product ion at *m/z 85* which could be produced by all acylcarnitine butyl esters, and a product at *m/z 103*, which has been reported as aliphatic hydroxyl group-containing fragment to produce the ion at *m/z 85*. These spectra indicate that m/z *248.1511* corresponds to hydroxybutyrylcarnitine +H.

Additional file 11 Extracted-ion chromatogram (XIC) and MS/MS spectra of *m/z 508. 34* (RT=1.70min and RT=1.83min) in QC sample and LysoPC (18:0) standard reference

Panel A and Panel C displaying the extracted-ion chromatogram of m/z *508.34* $[M\text{-}CH3]^-$ in QC sample and LysoPC (18:0) standard acquired in the negative ion mode. Panel B and Panel D exhibiting the MS/MS spectra of *m/z 508.34* $[M\text{-}CH3]^-$ in QC sample and LysoPC (18:0) standard. Each arrow in MS/MS spectrum of LysoPC (18:0) indicates a reported site of fragmentation, with the most intense product ion at *m/z 283.2639* corresponding to fatty acid 18:0. The other less abundant product ion at *m/z 168* corresponds to N-dimethylaminoethylphosphate anion, and ions at *m/z 224* and *m/z 242* to the products of ketene losses from demethylated lysoPC (18:0). These spectra confirmed the identification of *m/z 508.34* (RT=1.70min, RT=1.83min) as LysoPC (18:0)–CH3.

Additional file 12 MS/MS spectra of *m/z 506.3249* (ESI-, RT=1.38min) and its inferred chemical structure

Product ion spectra obtained from MS/MS of *m/z 506.3249* [M-CH3]$^-$ in the negative ion mode. Each arrow indicates a possible site of fragmentation, with the most intense product ion at *m/z 281.2483* corresponding to 18:1 fatty acid. The spectra indicate that m/z *506.3249* corresponds to of LysoPC (18:1)–CH3.

Additional file 13 MS/MS spectra of *m/z 504.3093* (ESI-, RT=1.12min) and its inferred chemical structure

Product ion spectra obtained from MS/MS of *m/z 504.3093* [M-CH3]$^-$ in the negative ion mode. Each arrow indicates a possible site of fragmentation, with the most intense product ion at *m/z 279.2326* corresponding to 18:2 fatty acid. These spectra indicate that m/z *504.3093* corresponds to of LysoPC (18:2)–CH3.

Additional file 14 The base peak intensity (BPI) of Precursors (MS1) in positive ion mode (PIM) across the whole mass range

Panel A-C indicating test plasma sample with LC Gradient of 20min, 15min and 10min, Panel D indicates QC sample from this study with RT of 10min. As shown in Panel D, the common high abundant precursor ions may represent characteristic patterns corresponding to certain lipid species extracted from human plasma. For instance, the ions at *m/z 496.35* (RT=1.31min), *m/z 524.38* (RT=1.81min) and *m/z 758.58* (RT=6.30min) may be suggested as [lysoPC (16:0)+H]$^+$, [lysoPC (18:0)+H]$^+$ and [PC(16:0/18:2)+H]$^+$ respectively [47], the ions at *m/z 780.56* (RT=5.15min) and *m/z 782.57* (RT=5.33min) as [PC(36:5)+H]$^+$ and [PC(34:1)+Na]$^+$ and ions at *m/z 369.35* (RT=8.47min) as [cholesterol-H2O+H]$^+$, a cholestadiene cation generated from cholesteryl esters (CE) [48]. And the abundant ion at m/z 577.52 (RT=8.42min) has been reported to indicate the sodiated 18:2 fatty acyl group containing a keto moiety formed by TG species [49].

Additional file 15 The base peak intensity (BPI) of fragments (MS2) in PIM across the whole mass range

Panel A-C indicate test plasma sample with LC Gradient of 20min, 15min and 10min, Panel D indicates QC sample from this study with RT of 10min. The most abundant fragment ions at *m/z 184* which have been reported as protonated-phosphocholine moiety and that are diagnostic for the PC head group class [50,51].
