# Supplementary figures and images for: Lipidomic profiling reveals distinct differences in plasma lipid composition in healthy, prediabetic, and type 2 diabetic individuals
Source: Gigascience. 2017 May 15;6(7):1–12. doi: 10.1093/gigascience/gix036 (PMC5502363; doi:10.1093/gigascience/gix036)

**(A)****Positive**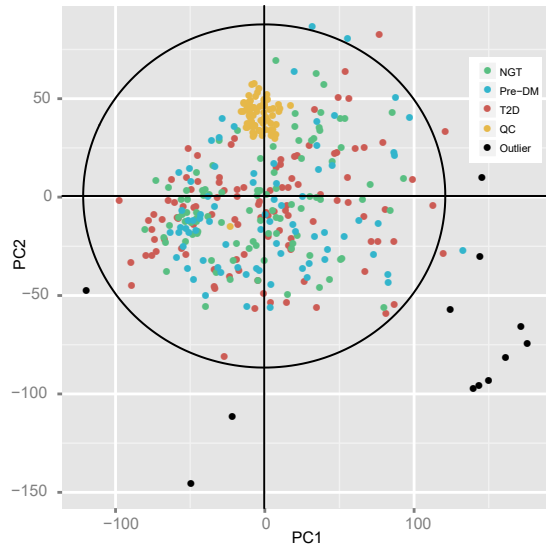**(B)****Negative**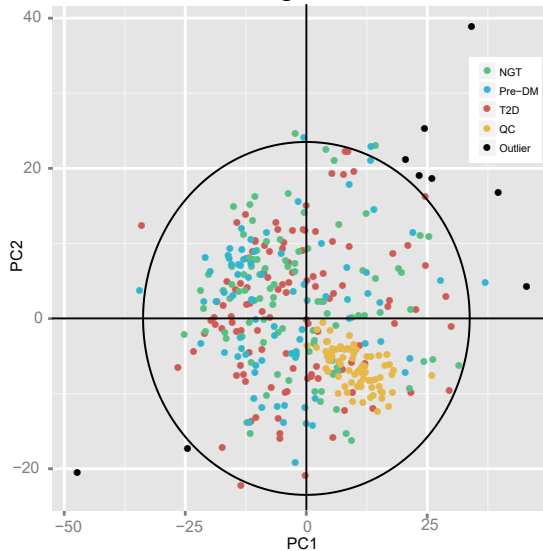

Supplement: Additional file 4: — principal component analysis of plasma lipid profiling from biological samples and quality controls. Principal component analysis was performed on all samples to identify run outliers and check for possible batch effects in both positive (A) and negative modes (B). The colors represent the different sample classes: green for normal glucose tolerant, blue for prediabetes, red for type 2 diabetes, orange for quality controls, and black for outliers. [file gix036_Additional_file_4.FIN.pdf]

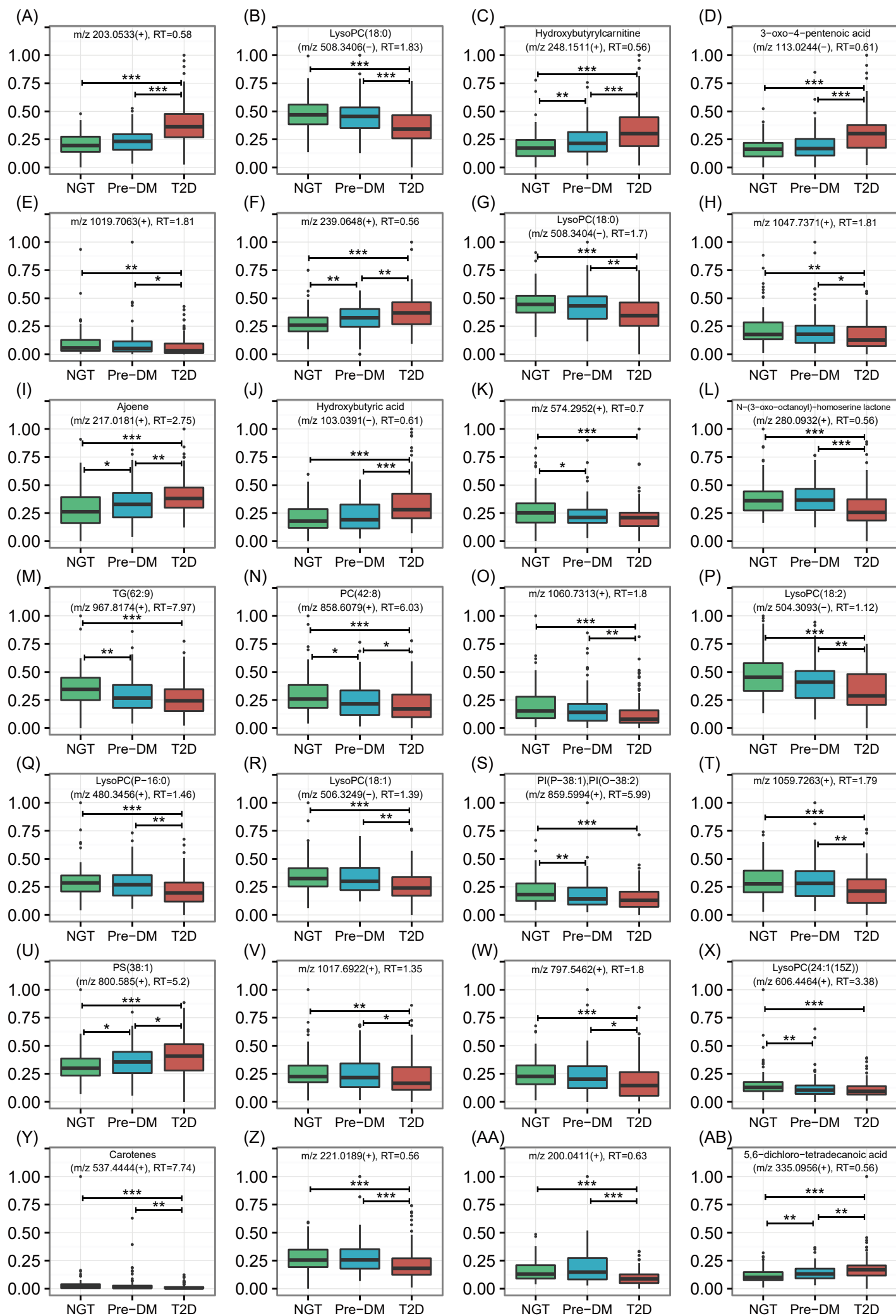

Supplement: Additional file 8: — box plot displays the relative intensity levels of 28 selected diabetic-related features in normal glucose tolerance, prediabetes, and type 2 diabetes. The features are presented in order of decreasing importance according to the selection frequencies in a random forest model. One asterisk denotes P < 0.05, 2 denote P < 0.01, and 3 denote P < 0.001 (Dunn's post hoc test). [file gix036_Additional_file_8.FIN.pdf]

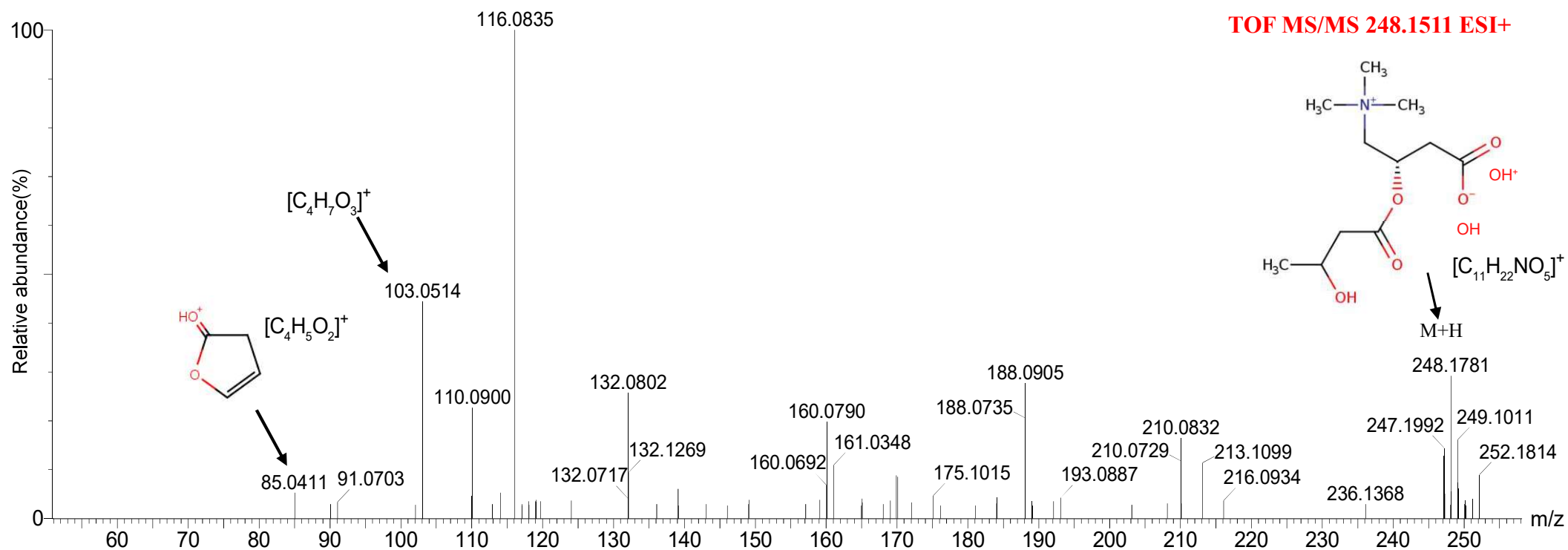

Supplement: Additional file 10: — tandem mass spectrometry spectra of m/z 248.1511 (ESI+, RT = 0.56 min) and its inferred chemical structure. Product ion spectra obtained from MS/MS of m/z 248.1511 [M+H]+ in the positive ion mode. Each arrow indicates a possible site of fragmentation, including a product ion at m/z 85, which could be produced by all acylcarnitine butyl esters, and a product at m/z 103, which has been reported as aliphatic hydroxyl group-containing fragment to produce the ion at m/z 85. These spectra indicate that m/z 248.1511 corresponds to hydroxybutyrylcarnitine +H. ESI+: positive electrospray ionization; RT: retention times. [file gix036_Additional_file_10.FIN.pdf]

A QC sample MS1

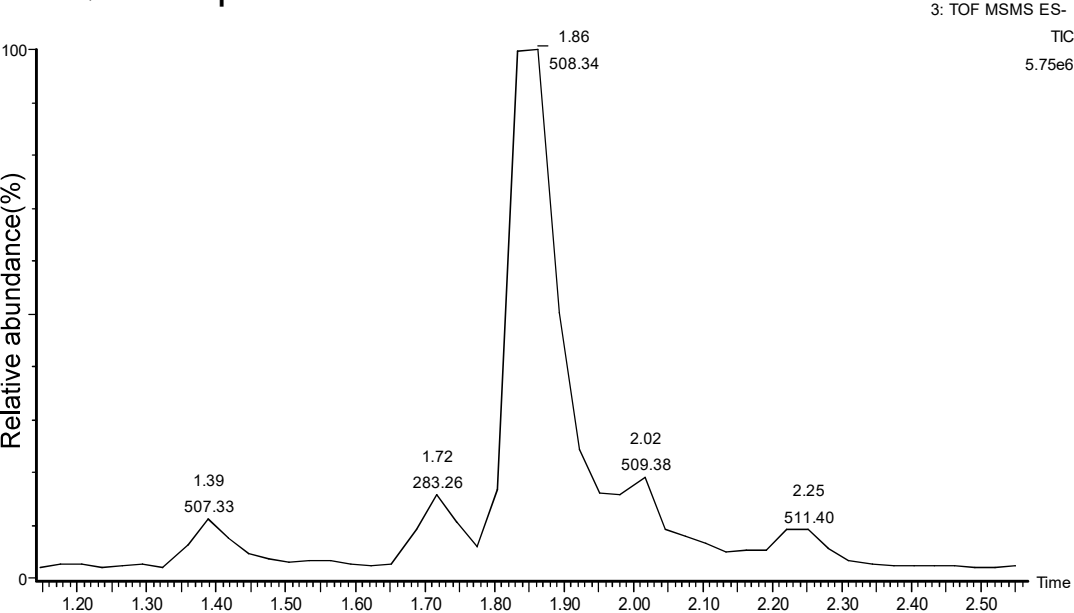

C QC sample MSMS

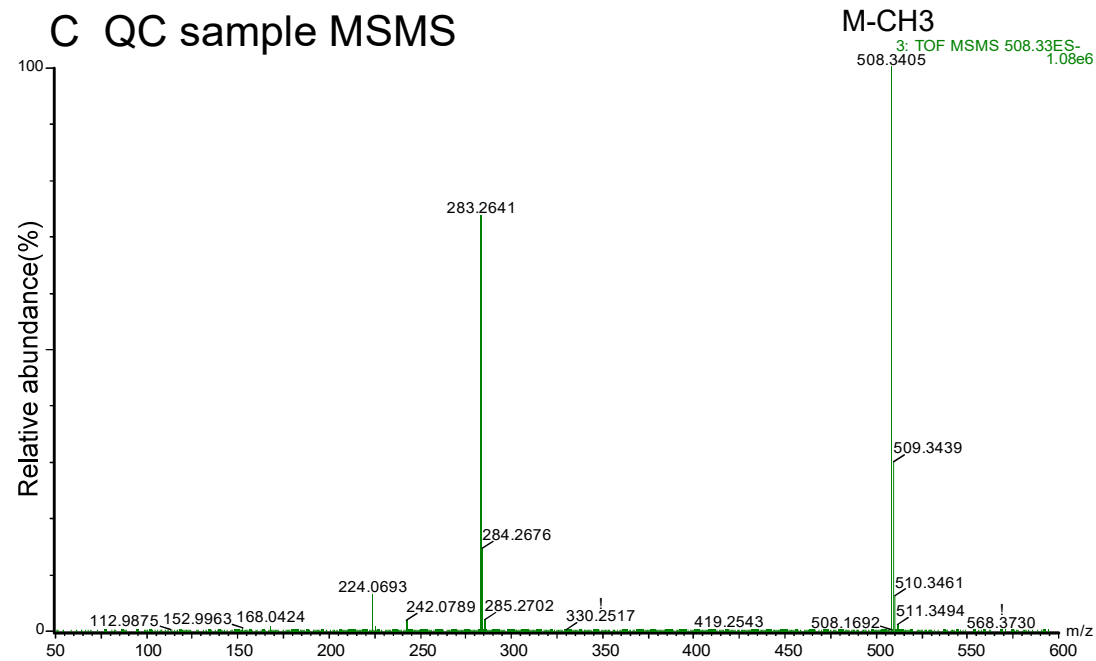

B Standard LysoPC(18:0) MS1

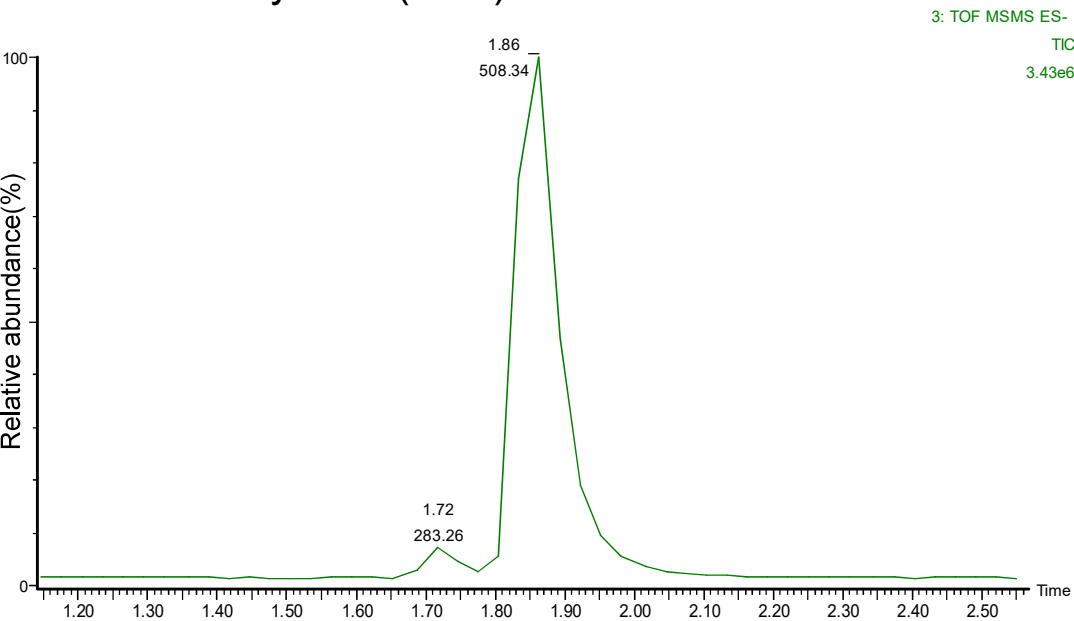

D Standard LysoPC(18:0) MSMS

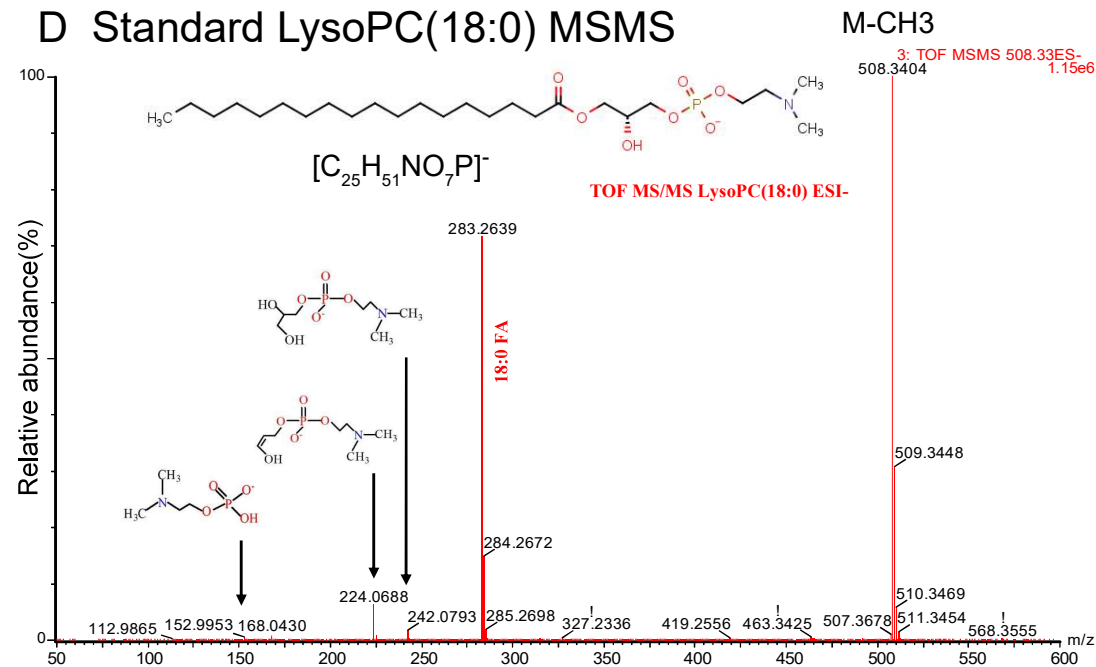

Supplement: Additional file 11: — extracted-ion chromatogram and tandem mass spectrometry spectra of m/z 508.34 (RT = 1.70 minutes and RT = 1.83 min) in quality control samples and lysophosphatidylcholine (18:0) standard reference. (A) and (C) display the extracted-ion chromatogram of m/z 508.34 [M–CH3]– in quality control sample and lysophosphatidylcholine (18:0) standard acquired in the negative ion mode. (B) and (D) exhibit the tandem mass spectrometry spectra of m/z 508.34 [M–CH3]– in the QC sample and lysophosphatidylcholine (18:0) standard. Each arrow in the MS/MS spectrum of lysophosphatidylcholine (18:0) indicates a reported site of fragmentation, with the most intense product ion at m/z 283.2639 corresponding to fatty acid 18:0. The other less abundant product ion at m/z 168 corresponds to N-dimethylaminoethylphosphate anion, and ions at m/z 224 and m/z 242 to the products of ketene losses from demethylated lysophosphatidylcholine (18:0). These spectra confirmed the identification of m/z 508.34 (RT = 1.70 min, RT = 1.83 min) as lysophosphatidylcholine (18:0)–CH3. [file gix036_Additional_file_11.FIN.pdf]

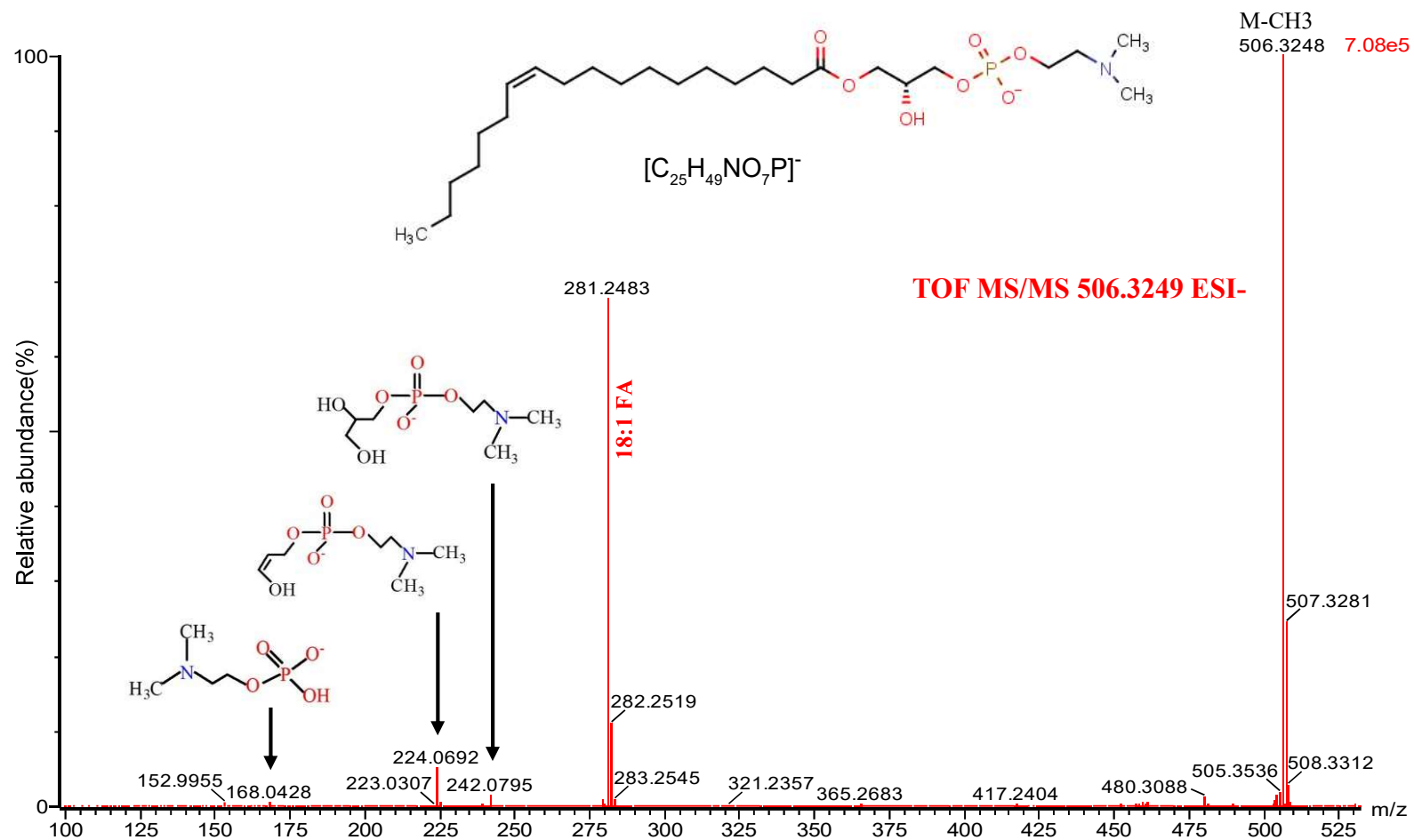

Supplement: Additional file 12: — tandem mass spectrometry spectra of m/z 506.3249 (ESI–, RT = 1.38 min) and its inferred chemical structure. Product ion spectra obtained from tandem mass spectrometry of m/z 506.3249 [M–CH3]– in the negative ion mode. Each arrow indicates a possible site of fragmentation, with the most intense product ion at m/z 281.2483 corresponding to 18:1 fatty acid. The spectra indicate that m/z 506.3249 corresponds to lysophosphatidylcholine (18:1)–CH3. [file gix036_Additional_file_12.FIN.pdf]

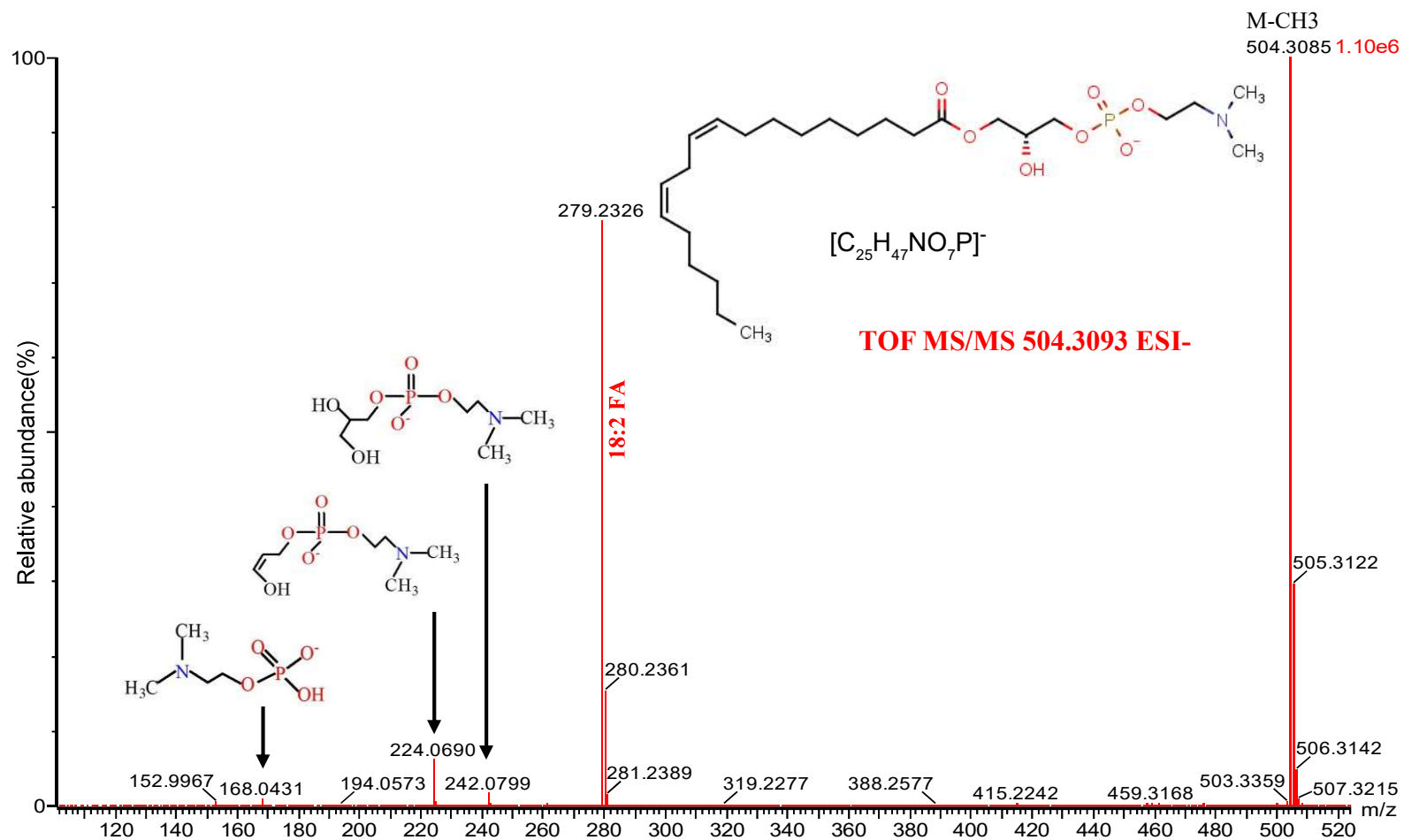

Supplement: Additional file 13: — tandem mass spectrometry spectra of m/z 504.3093 (ESI–, RT = 1.12 min) and its inferred chemical structure. Product ion spectra obtained from tandem mass spectrometry of m/z 504.3093 [M–CH3]– in the negative ion mode. Each arrow indicates a possible site of fragmentation, with the most intense product ion at m/z 279.2326 corresponding to 18:2 fatty acid. These spectra indicate that m/z 504.3093 corresponds to lysophosphatidylcholine (18:2)–CH3. [file gix036_Additional_file_13.FIN.pdf]

# POS\_MS1\_BPI\_Test

(A)

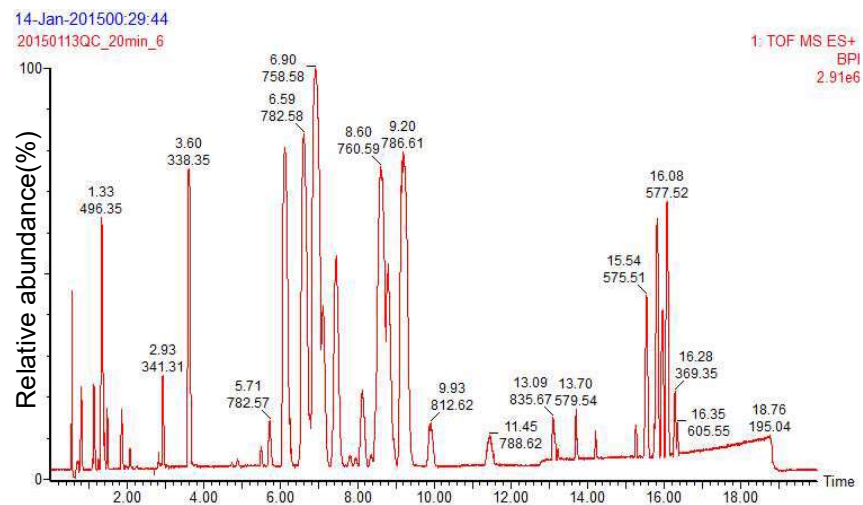

(B)

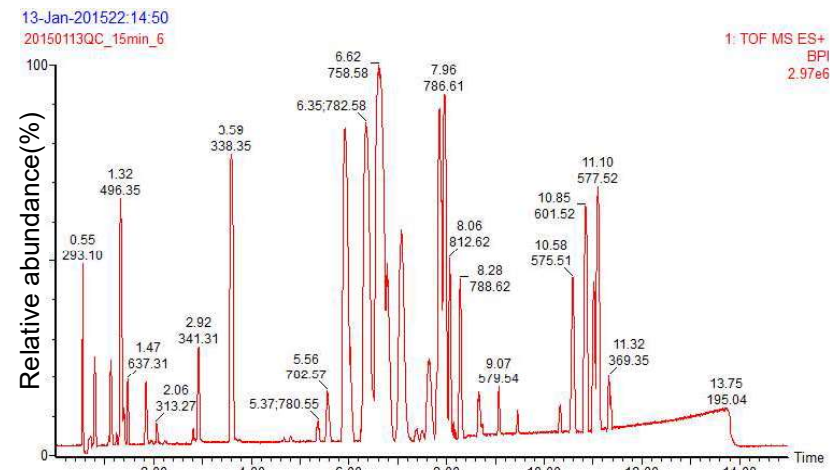

(C)

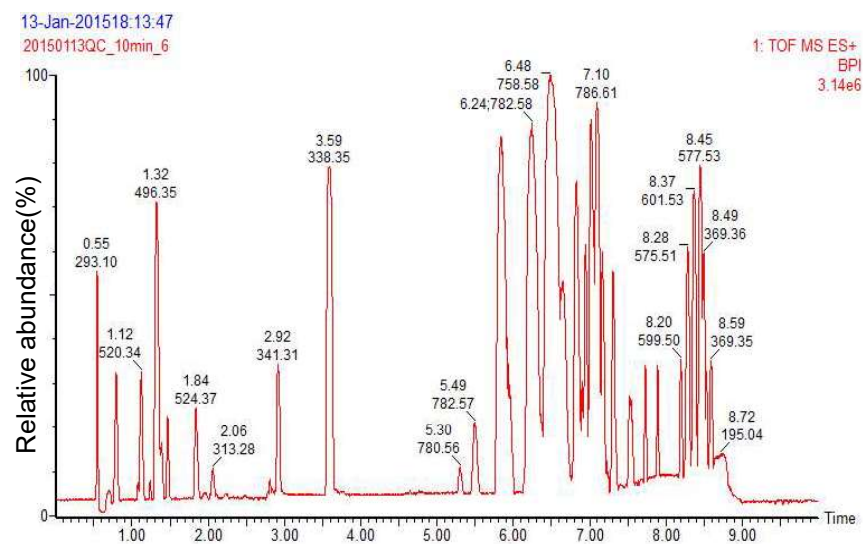

(D)

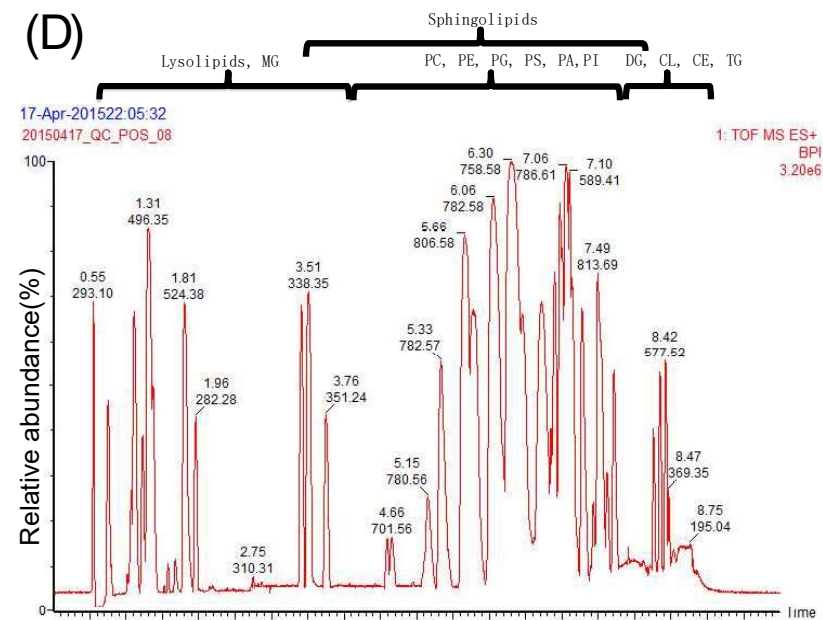

Supplement: Additional file 14: — the base peak intensity of precursors (MS1) in positive ion mode across the whole mass range. (A–C) indicate test plasma samples with liquid chromatography gradients of 20 minutes, 15 minutes, and 10 minutes. (D) indicates a quality control sample from this study with a retention time of 10 minutes. As shown in (D), the common high abundant precursor ions may represent characteristic patterns corresponding to certain lipid species extracted from human plasma. For instance, the ions at m/z 496.35 (RT = 1.31 min), m/z 524.38 (RT = 1.81 min), and m/z 758.58 (RT = 6.30 min) may be suggested as [lysophosphatidylcholine (16:0)+H]+, [lysophosphatidylcholine (18:0)+H]+, and [lysophosphatidylcholine (16:0/18:2)+H]+, respectively [50], the ions at m/z 780.56 (RT = 5.15 min) and m/z 782.57 (RT = 5.33 min) as [lysophosphatidylcholine (36:5)+H]+ and [lysophosphatidylcholine (34:1)+Na]+, and ions at m/z 369.35 (RT = 8.47 min) as [cholesterol–H2O+H]+, a cholestadiene cation generated from cholesteryl esters [51]. The abundant ion at m/z 577.52 (RT = 8.42 min) has been reported to indicate the sodiated 18:2 fatty acyl group containing a keto moiety formed by triglyceride species [52]. [file gix036_Additional_file_14.FIN.pdf]

# POS\_MSMS\_BPI\_Test

(A)

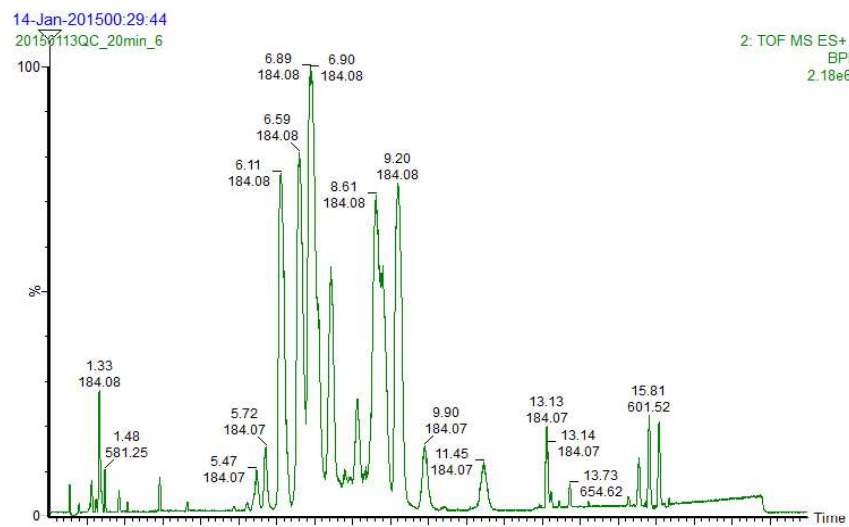

(B)

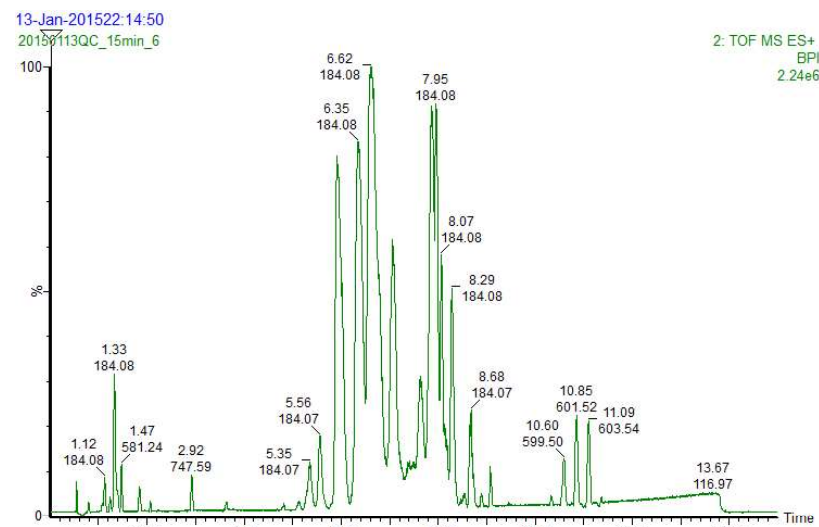

(C)

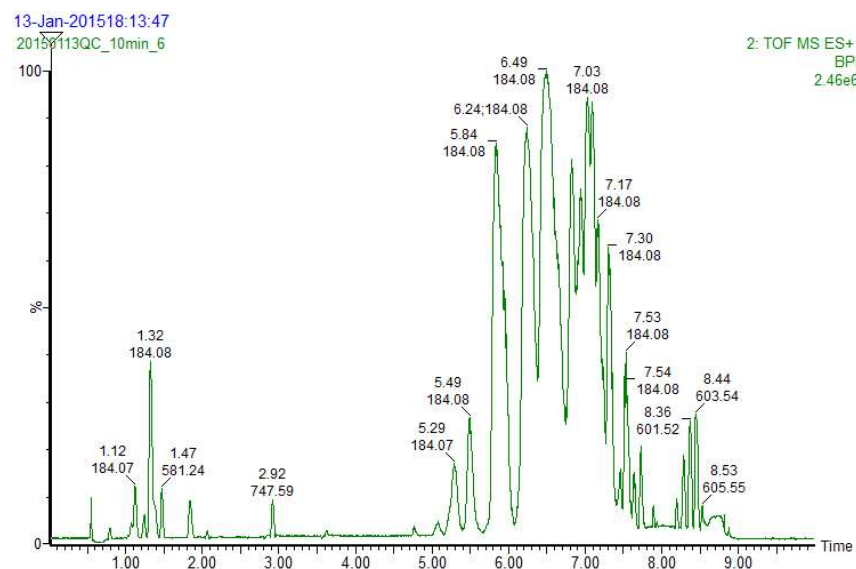

(D)

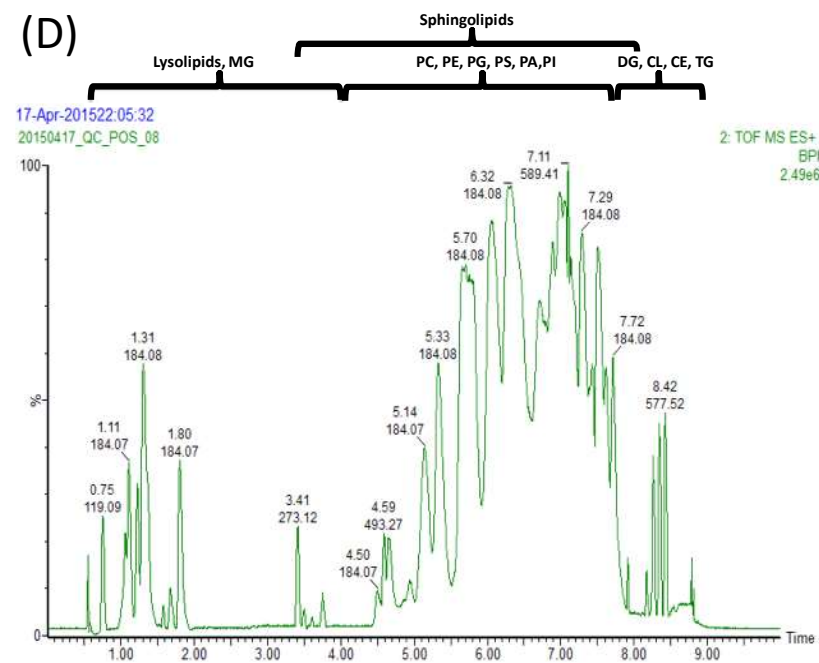

Supplement: Additional file 15: — the base peak intensity of fragments (MS2) in positive ion mode across the whole mass range. (A–C) indicate test plasma samples with liquid chromatography gradients of 20 minutes, 15 minutes, and 10 minutes. (D) indicates a quality control sample from this study with a retention time of 10 minutes. The most abundant fragment ions at m/z 184 have been reported as protonated-phosphocholine moieties that are diagnostic for the phosphatidylcholine head group class [53, 54]. [file gix036_Additional_file_15.FIN.pdf]
