# Supplementary material for: Lipidomic profiling reveals distinct differences in plasma lipid composition in healthy, prediabetic, and type 2 diabetic individuals
Source: Gigascience. 2017 May 15;6(7):1–12. doi: 10.1093/gigascience/gix036 (PMC5502363; doi:10.1093/gigascience/gix036)
Supplement: Response-to-reviewers_Original-Submission.pdf [file gix036_Response-to-reviewers_Original-Submission.pdf]

We appreciate the time and efforts by the editor and reviewers in reviewing this manuscript. We thank the reviewers for their helpful comments and suggestions. The manuscript has now been carefully revised to address their concerns, and we hope that the revised version will be acceptable for publication.

Reviewer #1:
Thank you for your very careful review of our paper, and for the comments, corrections and suggestions. We have revised the manuscript extensively following the comments and suggestions to improve the quality of the manuscript.

Than manuscript entitled “Lipidomic profiling reveals progressive changes of plasma 1 lipids from normal to type 2 diabetes" describes an untargeted lipidomic analysis of human plasma samples from NGT prediabetes and T2D. This was a challenging manuscript to review due in part to the poor quality of the grammar. There are so many errors (nine in the abstract alone) that in some places the meaning was lost and the reader is left to try and guess what is being said. I will leave it to the editors to decide how best to deal with this.

We sincerely apologize for the grammatical errors. We have carefully revised and corrected the manuscript.

The section on Data Description describes the cohort in the briefest of terms and then points to a data file. Then describes the profiling of lipids (again in the briefest of terms) and describes the data alignment and some statistical analysis. Then some description of QC samples and finally the dataset. This is simply confusing to the reader. Either describe the study in enough detail to be useful or simply list the data with RELEVANT information on its acquisition.

We have rewritten the Data Description part, and reorganized the paragraph structure according to the data analysis steps.

It is stated that 1590 features were significantly different, but this is before correction for multiple comparisons (the number after correction is around 80. This should be clearly state) Where are the assignments of the ~800 lipid species "significantly associated?

We thank the reviewer for the advice. In the revised version, we have now stated the number of significant features after correction for multiple testing and we have revised and annotated the relevant files.
As shown in Additional file 6, a total of 1,590 features differed significantly between the 3 groups, including 1,395 “positive” and 195 “negative” features ($p<0.05$, KW test), including 790 potentially matching lipids or lipid-like compounds. These 790 compounds included lysolipids, PC (phosphatidylcholines), carnitines, DG (diglyceride), TG and several free fatty acids. Further, 117 of the 1,590 features maintained significances after adjusting the false discovery rate (FDR) by Benjamini-Hochberg correction for multiple testing (Additional file 6, FDR <0.05) (Analyses, Page 6).

The development of multivariate classification models for T2D is a bit confusing and it is not really clear what the aim of this exercise is meant to be. The authors create a classification model to differentiate T2D from NGT, this is fine and it would be expected that plasma lipid species would do well in this account. But what is the clinical value of this as we already have a method to diagnose T2D? They then test this model on a mixture of T2D and prediabetes or on NGT and prediabetes where it performs relatively poorly, again all these classifications can be made with glucose measurements so what is the purpose of this modelling exercise? They further demonstrate that the "risk" of what? Is increased in some groups Fig 3F IGT, IFG/IGT but again what does this mean? I do not see anything that makes me think this group of markers has any clinical utility.

Thanks for pointing out the important view.

Type 2 Diabetes is heterogeneous metabolic disorders tightly related to perturbation of glucose and lipid metabolism. Several published studies have described hyperglycemia-independent effects of dyslipidemia on beta-cell function and diabetes complications such as atherosclerosis[1][2]. Of note, distinct differences in

fat distribution and accompanying risk of developing insulin resistance have been reported comparing Caucasians and Chinese. Accordingly, we hypothesize that detailed lipidomics analyses of Chinese individuals might provide information on possible molecular mechanism behind the ethnicity-specific differences, which eventually might be translated into clinical applications. Our study, for the first time, demonstrated global alterations in plasma lipidomic profiles between individuals in East China with different stages of T2D development, and we developed a RF-based classification models aiming to evaluate the use of plasma lipid profiles/markers to distinguish between healthy, prediabetic and T2D individuals and use this model for risk probability estimation.

As shown in Fig. 3F, using the RF-based model, we have observed a gradually increased T2D risk in 3 prediabetic subgroups from HbA1c5.7–6.4% to iIGT to combined IFG/IGT, basically consistent with the progression rates in similar prediabetic subgroups from a large-scale clinical observation[3]. Thus, our study has currently provided a highly-convinced example for the potential utility of plasma lipids on T2D risk stratification among prediabetes, which could not be achieved with glucose measurements or such common risk factors. Our work was also inspired by the previous study of Meikle and coworkers [4], in which, a lipid-based risk classification models to stratify T2D and IGT from NGT was successfully constructed. We now explicitly cite this work which we missed in the previous version, and discuss in more depth the performance of the classification models. We hope that this has increased clarity.

Furthermore, a large number of lipid features were independently associated with disease-related indices and this further points to lipidomics as a valuable tool for exploring possible lipid-related pathogenesis of T2D and monitoring T2D progression (Additional file 9). Together with earlier large studies, we are convinced that such detailed lipid profiling could and have provided a fundamental lipidomic view for Chinese individuals with NGT, prediabetes and T2D and the specific differential features could be used as a helpful starting for both deepening the mechanism for development of diabetes and assessing its risks.

So that leave the potential of these markers to inform on the biology of the disease, this was examined using correlation analysis with other measures of glucose homeostasis and anthropometric markers. However, I think this approach is flawed for a number of reasons. Interpretation of any correlation is clouded because there has been no allowance for covariates. All these analyses should have been done with regression analyses so that associations with each outcome could be examined independently from the other variables. For example, BMI are associated so when looking for correlations with age how can you exclude the effect of BMI?
And so on.

We thank the reviewer for the advice.
Accordingly, we performed regression analysis in relation to the analyses of the 1590 differential features (Additional file 9, Figure 4).
Three independent glm models were generated to calculate the correlation between lipid features and different phenotypes as follows,
Model 1 for lipid features and Age—de adjusting BMI, gender, diabetes status, hypertension history, hyperlipidemia history, smoking history and alcohol history
Model 2 for lipid features and BMI—by adjusting Age, gender, diabetes status, hypertension history, hyperlipidemia history, smoking history and alcohol history
Model 3 for lipid features and diabetes related indexes (FPG, 2h-PG, HbA1c, Insulin, C-peptide, HOMA-IR)—by adjusting Age, BMI, gender, hypertension history, hyperlipidemia history, smoking history and alcohol history.
In total, 81.38% (1294/1590) of the differential features correlated significantly with at least one of the diabetes-related indices after adjustment for age, gender, BMI, as well as hypertension, hyperlipidemia, smoking and alcohol history, with all RF selected features passing the cutoff point ($p<0.05$, Additional file 8) (Analyses, Page 7-Page 8).
We did observe some independent associations between BMI and lipid features after adjusting for the confounding effects of age, gender, diseases status and lifestyles. This information in now included in the revised manuscript. We hope that this revision addresses the issues raised by the reviewer.

With regard to the assignment of the lipid species I do not think that this was adequately explained, it is quite difficult to follow what was done and what level of confidence was attained. For example, LPC 17:0, LPC 17:1 and 17:2 were identified as 1.7/1.83_508.34m/z, 1.39_506.3249m/z and 1.12_504.3093 in negative ionisation mode. However, no features were detected in positive ion mode at the same retention time. LPC is significantly more sensitive in positive ionisation mode and it's difficult to see why it would not be detected in these analyses. Were they looked for?

We thank the reviewer for the insightful suggestion.
First, we agree that ionization of LPC species in the negative ionization mode is not common, and most annotated LPC species were also detected in the positive ion mode in our study (Additional file 3).
As mentioned in the revised methods section, the deprotonated molecule [M–H] −, the most common anion of lipids, was selected as the only adduct for database searching in the negative ion mode. Still, LPC and PC species hardly lose H+ in the negative ion mode, but can be detected as anions of acetate, chloride adducts and demethylated fragment ions [5] [6] [7].
Taken together, the previous features LysoPC 17:0 (m/z 1.7/1.83_508.34), LysoPC 17:1 (m/z 1.39_506.3249) and LysoPC 17:2 (m/z 1.12_504.3093) have been manually reannotated to LysoPC 18:0 (1.7/1.83_508.34m/z), LysoPC 18:1 (1.39_506.3249m/z) and LysoPC 18:2 (1.12_504.3093m/z) by using the anionized adduct [M–CH3] −. As shown in the Additional file 11-13, peaks at m/z 283.2641, 281.2483 and 279.2326 correspond to the abundant acyl anion fragments of stearic, oleic and linoleic acid (18:0, 18:1 and 18:2), respectively, and the characteristic m/z 224 fragment to ketene loss from these demethylated lysoPCs. Further, by comparing the retention time and fragmentation patterns with the authentic reference standards of LysoPC (18:0), the two lipid features at m/z 508.3404 and m/z 508.3406 were verified (Additional file 11).

According to the suggestion from the reviewer, we have also rechecked the mass signals from the positive ion mode and found putative matching compounds in both ion modes. For example, m/z 1.67_524.3728 and m/z 1.80_523.3651n in the positive ion mode were the same compounds as m/z 1.7/1.83_508.34 in the negative ion mode (LysoPC 18:0); m/z 1.35_504.3462 in the positive ion mode was the same compound as m/z 1.39_506.3249 in the negative ion mode (LysoPC 18:1); m/z 1.11_519.3351n in the positive ion mode was the same compound as m/z 1.12_504.3093 in the negative ion mode (LysoPC 18:2) (Additional file 3). In addition, these features detected in the positive ion mode all showed significant higher levels in NGT than in T2D individuals (Dunn's post hoc test, $p<0.05$, Additional file 6).

It is not clear how close the mass assignment was for these compounds and while some proposed fragmentation data is provided, for some compounds, it is not clear how these were derived.

We apologize for this confusion.
The MS/MS datasets were generated on the Waters XEVO-G2XS QTOF instrument and processed using commercial software Progenesis QI 2.0, consisting of raw data import, selection of possible adducts, peak set alignment, peak detection, deconvolution, dataset filtering, noise reduction, compound identification and normalization using the sum method. The analysis parameters used were as follows: 1) possible adducts of [M+H]+, [M+H-H2O]+ , [M+Na]+ and [M+K]+ for ESI+ and [M-H]- for ESI-, 2) the retention time of 0.5-9min, 3) the peak width of 1-30s, 4) 10 ppm mass tolerance for the precursors, 5) 10ppm fragment mass tolerance for theoretical fragmentation searching to improve the confidence in compound identification. The commercial software Progenesis QI 2.0 is not publicly available. We now have stated this clearly (Methods, Page 16).

The normalized peak data was further preprocessed by an in-house software metaX consisting of a series of data filters. The LC-MS/MS features with high resolution and accuracy were identified using Progenesis QI 2.0 by searching in the public databases including Human Metabolome Database (HMDB, version 3.6, http://www.hmdb.ca/), LIPID MAPS Structure Database (LMSD, http://www.lipidmaps.org/) and LipidBlast with the mentioned parameters. The metabolites matched to LMSD, LipidBlast or the aliphatic compounds of HMDB at the Molecular Framework level were considered lipids and lipid-like features. The raw annotation

files were further filtered using defined ranges of retention times of different lipid species according to previous studies[7,8].Data-dependent analysis and reference standards were used to aid in identifying the lipids (Methods, Page 17).

Standard compounds should have been used to validate at least some of the key metabolites identified. It does not appear that elution time was factored in when identifying the compounds, for example one of the identified compounds (PC16:1/2:0) was significantly associated with diabetes. The identification may be wrong as elution time was 0.7minutes whereas compounds such as lysoPC 17:1 eluted at 1.39 minutes. It's also very unlikely that DG's (43:6, 48:6) would elute <2minutes next to the lyso phospholipids. Here again standards would be very helpful.

Thanks for raising this important point.
As suggested, we have added retention time as a characteristic for lipid identification and revised the original inadequate annotations of potential lipid features.
In this study, the lipids were separated using CSH C18 ACQUITY UPLC System (2.1×100 mm, 1.7 μm, Waters) with the same mobile-phase composition according to the application note of the CSH C18 UPLC System [7].
Before the large-scale study, we tested and optimized the gradient and range of retention time in relation to separation and detection. As shown by the test samples, both abundant lipid precursors ions and fragments could be separated with similar peak shapes and ion intensities using the accelerated elution protocol (Additional file 14A-C, Additional file 15A-C). Further, the mixed QC samples with RT of 10min from this study also showed similar base peak intensities (BPI) of precursors and fragments with the test sample (Additional file 14D, Additional file 15D). Considering the large sample size of this study, we choose the accelerated elution profile.
As reported in the application note[7], the lysophospolipids such as lysoPC, lysoPE, LysoPG, LysoPS and LysoPI species and several MG species were the first early eluting compounds in the positive ion mode and several FA species eluted early in the negative ion mode (Table 1) [7]. For example, the Lyso17:0 eluted at 1.5min in the positive ion mode (Figure 3, peak 8) [7]. Lipid species from PC, PE, PG, PS, PA were then in turn eluted followed by lysolipids and the DG, CL, CE and TG species which containing longer acyl chains as the preferred end products (Figure 5) [7]. And the sphingolipids such as SM and Cer species could be eluted at a wide time range, such as the d18:1/12:0 SM was eluted at 3.49min and the d18:1/25:0 Cer was eluted at 14.49min (Table 1) [7]. Similar elution orders of plasma lipid species were also provided by Sarafian’s group[8].

Thus, we have filtered the annotations of lipid features according to the following retention times, 0.5-4min for lysophospolipids including LysoPC, LysoPE, LysoPG, LysoPS, LysoPA and LysoPI species, 3-8.1min for sphingolipids, including SM, Cer and LacCer, GluCer and LacCer species, 4-7.8min for PC, PE, PG, PS, PA and PI species, 7.8-9.5min for DG, TG and CE species in the positive ion mode; 0.5-4min for lysophospolipids and FFA species, 4-9min for PC, PE, PG, PS, PA and PI and sphingolipid species (Additional file 14D, Additional file 15D). For instance, the PC 16:1/2:0 at m/z 574.2952 (RT=0.7min) was reannotated to unknown features (NA) (Additional file 7). And the previous LysoPC 17:0, LysoPC 17:1 and LysoPC 17:2 in the negative ion mode have been manually reannotated to LysoPC 18:0, LysoPC 18:1 and LysoPC 18:2, respectively, which we have detailed mentioned in the previous response.

Unfortunately, although this study has a lot of potential to provide interesting insight to metabolism in T2D, the analytical approach and validation do not provide sufficient confidence in the conclusions drawn. I suggest a complete rethink of what is trying to be achieved here and then a careful analysis to address the specific questions under investigation.

We sincerely thank you once again for constructive criticisms and valuable suggestions, and we have performed a major revision of the paper in order to improve 1) the quality of the language, 2) the interpretation of correlation analyses by using general linear model adding covariates, 3) the lipid identification by correcting the original annotation of those molecular with inadequate annotation. We hope that this revision has improved the manuscript sufficiently to be published in GigaScience.

Reference:
1. Goldberg IJ. Diabetic Dyslipidemia: Causes and Consequences. J. Clin. Endocrinol. Metab. 2001;86:965–71.
2. Bardini G, Rotella CM, Giannini S. Dyslipidemia and diabetes: reciprocal impact of impaired lipid metabolism and Beta-cell dysfunction on micro- and macrovascular complications. Rev. Diabet. Stud. 2012;9:82–93.
3. Morris DH, Khunti K, Achana F, Srinivasan B, Gray LJ, Davies MJ, et al. Progression rates from HbA1c 6.0-6.4% and other prediabetes definitions to type 2 diabetes: A meta-analysis. Diabetologia. 2013;56:1489–93.
4. Wong G, Barlow CK, Weir JM, Jowett JBM, Magliano DJ, Zimmet P, et al. Inclusion of Plasma Lipid Species Improves Classification of Individuals at Risk of Type 2 Diabetes. PLoS One. 2013;8.
5. Ekroos K, Ejsing CS, Bahr U, Karas M, Simons K, Shevchenko A. Charting molecular composition of phosphatidylcholines by fatty acid scanning and ion trap MS3 fragmentation. J. Lipid Res. 2003;44:2181–92.
6. Taguchi R, Ishikawa M. Precise and global identification of phospholipid molecular species by an Orbitrap mass spectrometer and automated search engine Lipid Search. J. Chromatogr. A. 2010;1217:4229–39.
7. Isaac G, Mcdonald S, Astarita G. Lipid Separtion using UPLC with Charged Surface Hybrid Technology. Waters Corp. Milford, MA, USA. 2011;1–8.
8. Sarafian MH, Gaudin M, Lewis MR, Martin FP, Holmes E, Nicholson JK, et al. Objective set of criteria for optimization of sample preparation procedures for ultra-high throughput untargeted blood plasma lipid profiling by ultra performance liquid chromatography-mass spectrometry. Anal. Chem. 2014;86:5766–74.

Reviewer #2: Authors have performed untargeted lipidomic profiling of 293 subjects having Type 2 diabetes (T2D), prediabetes, or normal glucose tolerance (NGT). This study identifies 28 features that could distinguish T2D subjects from NGT.

Thank you for all of your detailed comments and suggestions. We found them very useful for the revision.

First, the title suggests a progressive change in lipid profiles from NGT to T2D. However, I believe this could only be achieved if subjects are followed over the period of time and lipid profiling is performed at various time points. With a cross-sectional cohort like this, it could not be termed as progressive changes. At the best, this demonstrates the differences in lipid profiles between NGT, prediabetes, and T2D.

We thank the reviewer for the advice.
Accordingly, the title has been changed to “Lipidomics profiling reveals distinct differences in plasma lipid composition in healthy, prediabetic and type 2 diabetic individuals”.

Authors have used random forest classifier to identify the features and build the classifier. They have randomly divided the data into two groups, for testing and validation. However, more thorough analysis will be based on the k-fold external cross-validation, where feature selection is integrated within cross-validation. Any single random split could provide biased results.

We thank the reviewer for the advice and apologize for the confusion.

In this study, we first randomly divided samples from T2D and NGT individuals into two subgroups (70 T2D/70 NGT for training set, 44 T2D/28 NGT for test set). A nested cross validation was conducted including an inner loop and an outer loop. The inner loop was used for model selection where 5 repetitions of 10-fold cross-validation were computed on the training set and the outer loop for external validation with the independent test set to get a fair estimate of model performance.

Following the suggestion of the reviewer, the random split of training and test sets was repeated 1000 times. An integrated AUC of 84.33% was determined using the test samples from 1000 splits (95% CI =

84%-84.67%) (FigureR1. A). The repeat results indicated stable performances of plasma lipids in distinguishing T2D and NGT samples and suggested a comparable performance (AUC=86.24%, 95% CI =76.05%-96.43%, Fig. 3C) could be estimated from our single random split. Moreover, the frequencies of lipidomic features on 1000 random splits were also calculated.
As presented in FigureR1. B, 15 of our 28 selected features reached relatively high selection frequencies over 300. Such lipid features including m/z 203.0533 (ESI+, RT=0.58min) of 1000 selected times; hydroxybutyrylcarnitine at m/z 248.1511(ESI +, RT=0.58min) of 950 selected times; TG (62:9) at m/z 967.8174 (ESI +, RT=7.97min) of 889 selected times most likely represent true T2D-related "signals". The average frequencies of these 28 features were reached 385 times. Taken together, our results indicated that a model with 28 selected features could serve as an unbiased RF-based T2D risk prediction model. The feature selection frequency for multiple random cross-validation is presented in Table R1.

FigureR1 Multiple random cross-validation based on untargeted lipidomics data
Receiver operating curve (ROC) and area under receiver operating curve (AUC) in test samples derived from 1000 random splits, (B) Scatterplot for feature selection frequencies (SF) on 1000 random splits (1, above) with 345 features of SF ≥ 10 times, and on our single random split with 5 repetitions of 10-fold cross-validation (2, below). The red cycle denotes the 28 selected features and the black denotes non-selected features.

KW-test and Spearman's correlation analysis doesn't take into account the confounders. I would suggest authors to use GLM analysis for all the features adjusting for suitable covariates, not just for the selected features.

We thank the reviewer for the points.
In this study, we attempted to describe differences among three clinical groups in relation to the development on T2D and evaluate the reliability of the selected diabetes-related features.
The non-parametric Kruskal-Wallis test is commonly used to determine if there are statistically significant differences among more than two independent groups. To adjust the false discovery rate (FDR) , Benjamini-Hochberg multiple testing correction was conducted with 117 of the 1590 features passing the criterion with FDR<0.05 (Additional file 6). In addition, we considered the potential effects of CCBs treatment (a class of antihypertension drugs which were widely used in our cohort, n=61) on plasma lipids as evaluated by PERMANOVA analyses (Additional file 5), and we then conducted the blocked Kruskal-Wallis test using CCBs treatment as a confounder.
Together, by adjusting for such potentially confounding effects and false discovery rate, we believe that Kruskal-Wallis test is a useful tool for detection of plasma lipid differences between different T2D development stages, especially in prediabetes, the very important precursor stage before diabetes (Additional file 6).

On the other hand, considering that no population stratified analyses were conducted before group comparisons, we agree that a multivariable regression model would be more objective than Spearman's correlation analysis for relationship between lipidomic variables and clinical phenotypes to adjust for effects of potential confounders. Therefore, we have performed GLM analyses on the 1590 features that passed the KW test (p<0.05) to provide further unbiased information between diabetic indices and these features (Additional file 9). We have also updated Figure 4 by using the correlation of GLM instead of Spearman's correlation (Figure 4 Correlations between the phenotypes and the 28 RF-based selected features). For further details, please see our response to the regression analyses question of Reviewer #1.

Some important references are missing, for example Wong et al PLoS ONE 8(10): e76577. A discussion on previously identified features for T2D classification is also important.
Thank for you drawing our attention to this point. We have now cited Wong et al. (PLoS ONE 8(10): e76577) and elaborated more on this point in our discussion section.

Reviewer #3: The authors have submitted a Research article which reports on a metabolomics study using

liquid chromatography mass spectrometry to look into the differences in lipid profiles from 293 East Asian people with pre-diabetes (81), type 2 diabetes (T2D) (1w14) and individuals with normal glucose tolerance (NGT) (98). The lipid profiles of individuals with pre-diabetes and T2D were similar to each other but different to people with NGT. I believe that the data set generated by the authors of this study can be useful to the scientific community for understanding the abnormal lipid metabolism associated with diabetic disease.
We thank the reviewer very much for your kind words about our paper.
We are delighted to learn that you think our work provide useful information in relation to understanding the abnormal lipid metabolism associated with diabetic disease.

However, there are a number of issues with the manuscript and the work it describes before it can be published in GigaScience:
1. The title seems misleading since it contains the word, "progressive". The use of this word suggests a longitudinal study in which the lipid profiles of the 293 individuals have been tracked over a period of time. In fact, the study described in the manuscript is a cross-sectional study in which the lipid profiles of individuals have been identified at a point in time when they either have NGT, pre-diabetes or T2D. The title should be re-worded accordingly.

We thank the reviewer for the advice.
The title has been changed to “Lipidomics profiling reveals distinct differences in plasma lipid composition in healthy, prediabetic and type 2 diabetic individuals”

2. The science in the paper is difficult to understand due to grammatical errors present throughout the manuscript.
We sincerely apologize for the grammatical errors. We have carefully revised the manuscript and corrected the grammar.

I appreciate that the authors have performed untargeted profiling of lipids associated with diabetes for the first time in the Chinese population - this is a valuable scientific contribution. However, the application of the Random Forest classifier and the association of human phenotypic markers with specific lipid metabolites could be more concisely and clearly reported in their relevant sections of the manuscript.
We appreciate this comment.
We apologize for the confusion of the random forest analysis and correlation analysis. We have gone through the paper to improve clarity and language. We hope that this revision is adequate.

In general, the standard of written English in the manuscript needs to be improved before it can be published.

Again, we sincerely apologize for the grammatical errors and unclear presentation. We have carefully revised and corrected the manuscript before resubmission.

GigaScience has a focus on reproducible science and requires a "Availability of Data and Materials" section in manuscripts which reports where readers can obtain the data, and the analysis tools and scripts used to generate the results reported in the manuscript. This section should be added into a revised manuscript. The text describing that the raw MS data have been deposited in the MetaboLights database should be moved into this section.
We thank the reviewer for the suggestion.
Per your advice, the paragraph “Availability of supporting data and materials” has been added to the revised version.

I appreciate that the study uses commercial software in the form of Progenesis to normalize MS data, detect peaks and assign them with metabolite identities, and this tool is not be publicly available. This is fine with me but it should be stated.
We are so sorry for this negligence and thank the reviewer for the kind reminder.

Information on the commercial software Progenesis QI used for the study has been included in the section “Availability of supporting data and materials” and in the revised method section.

However, other software tools and scripts responsible for generating the univariate and multivariate statistical results should, where possible, be provided and/or deposited in a Github repository.
We thank the reviewer for the suggestion.
The availability of the software tools and scripts responsible for generating the univariate and multivariate statistical results has now been clearly stated in the above paragraph and deposited in the GigaScience GigaDB repository.
