# Supplementary material for: Lipidomic profiling reveals distinct differences in plasma lipid composition in healthy, prediabetic, and type 2 diabetic individuals
Source: Gigascience. 2017 May 15;6(7):1–12. doi: 10.1093/gigascience/gix036 (PMC5502363; doi:10.1093/gigascience/gix036)
Supplement: Response-to-Reviewers_Revision-1.pdf [file gix036_Response-to-Reviewers_Revision-1.pdf]

Dear Dr. Nicole Nogoy,

We are very pleased by the positive comments by the reviewer. Below please find our responses to the remaining issues raised by the reviewer.

Reviewer #1: The authors have made substantial modifications to the paper and performed additional analyses which helps with interpretation.

The reference to the model reported by Meikle et al should be clarified as they were classifying only individuals who had FPG<6.1 and so would not be classified as diabetes or prediabetes based solely on the FPG. Thus there was potential clinical utility in this model. In the current analysis, no such distinction was made and so the purpose of the modelling is rather to demonstrate a continuum of the degree of alteration in the lipid profile in line with the clinical presentation. I think the authors make this point but it could be stated more explicitly so that it is clear that the models are not proposed to have clinical use (although the markers may well have clinical use if this can be demonstrated in a longitudinal cohort.

I think if these items can be clarified the manuscript would be suitable for publication.

Response: We acknowledge the points made by the reviewer and we have accordingly modified the text:

In the Background, we now state (page 2-3, lines 50-52): "Additionally, T2D risk classification models of potential value for clinical use have been developed and evaluated by Meikle and coworkers to stratify individuals with an FPG <6.1 mmol/L using lipidomics profiles with or without non-lipid risk factors.". In the discussion, we now state (page9-10, lines 223-225): "Interestingly, Meikle's groups has developed several type 2 diabetes risk classification models to evaluate their performances in relation to stratifying type 2 diabetes and IGT from NGT, all with FPG <6.1 mmol/L."

Further, in the Discussion we now state (page 9, lines 217-223): "The T2D risk of similar prediabetic subgroups in this study, HbA1c5.7–6.4%, iIGT and combined IFG/IGT, was assessed using the RF algorithm, which predicted a gradually increased T2D risk from HbA1c5.7–6.4% to iIGT to combined IFG/IGT. This prediction is overall consistent with the progression rates determined in the analysis of Morris et al. [20], but we also emphasize that a thorough statistical validation using longitudinal studies of larger cohort will be required to demonstrate the clinical utility of our model in the Han Chinese population. The low risk probability predicted for prediabetic individuals diagnosed with elevated HbA1c suggests that appropriate HbA1c criteria for Chinese prediabetic individuals should also be determined."

The reviewer also noted that your manuscript is difficult to read - please ensure that you have someone appropriate to read through and edit your manuscript.

Response: We have scrutinized the manuscript to correct grammatical mistakes and aimed to make the manuscript easier to read, but admittedly, the density of metabolomics data is heavy. All changes to improve readability of the manuscript are marked with text in blue.
