# Supplementary material for: Lipidomic profiling reveals distinct differences in plasma lipid composition in healthy, prediabetic, and type 2 diabetic individuals
Source: Gigascience. 2017 May 15;6(7):1–12. doi: 10.1093/gigascience/gix036 (PMC5502363; doi:10.1093/gigascience/gix036)
Supplement: Reviewer-1-Report-(Original-Submission).pdf [file gix036_Reviewer-1-Report-(Original-Submission).pdf]

Reviewer Report

Title: "Lipidomic profiling reveals distinct differences in plasma lipid composition in healthy, prediabetic and type 2 diabetic individuals"

Version: Original Submission **Date:** 11/10/2016

Reviewer name: Peter Meikle

Reviewer Comments to Author:

Than manuscript entitled Lipidomic profiling reveals progressive changes of plasma 1 lipids from normal to type 2" describes an untargeted lipidomic analysis of human plasma samples from NGT prediabetes and T2D.

This was a challenging manuscript to review due in part to the poor quality of the grammar. There are so many errors (nine in the abstract alone) that in some places the meaning was lost and the reader is left to try and guess what is being said. I will leave it to the editors to decide how best to deal with this.

The section on Data Description describes the cohort in the briefest of terms and then points to a data file. Then describes the profiling of lipids (again in the briefest of terms) and describes the data alignment and some statistical analysis. Then some description of QC samples and finally the dataset. This is simply confusing to the reader. Either describe the study in enough detail to be useful or simply list the data with RELEVANT information on its acquisition.

It is stated that 1590 features were significantly different, but this is before correction for multiple comparisons (the number after correction is around 80. This should be clearly stated

Where are the assignments of the ~800 lipid species "significantly associated?

The development of multivariate classification models for T2D is a bit confusing and it is not really clear what the aim of this exercise is meant to be. The authors create a classification model to differentiate T2D from NGT, this is fine and it would be expected that plasma lipid species would do well in this account. But what is the clinical value of this as we already have a method to diagnose T2D? They then test this model on a mixture of T2D and prediabetes or on NGT an prediabetes where it performs relatively poorly, again all these classifications can be made with glucose measurements so what is the purpose of this modelling exercise?

They further demonstrate that the "risk" of what? Is increased in some groups Fig 3F IGT, IFG/IGT but again what does this mean? I do not see anything that makes me think this group of markers has any clinical utility.

So that leave the potential of these markers to inform on the biology of the disease, this was examined using correlation analysis with other measures of glucose homeostasis and anthropometric markers. However, I think this approach is flawed for a number of reasons. Interpretation of any correlation is clouded because there has been no allowance for covariates. All these analyses should have been done

with regression analyses so that associations with each outcome could be examined independently from the other variables. For example and and BMI are associated so when looking for correlations with age how can you exclude the effect of BMI? And so on.

With regard to the assignment of the lipid species I do not think that this was adequately explained, it is quite difficult to follow what was done and what level of confidence was attained.

For example LPC 17:0, LPC 17:1 and 17:2 were identified as 1.7/1.83_508.34m/z, 1.39_506.3249m/z and 1.12_5.04.3093 in negative ionisation mode. However no features were detected in positive ion mode at the same retention time. LPC is significantly more sensitive in positive ionisation mode and it's difficult to see why it would not be detected in these analyses. Were they looked for?

It is not clear how close the mass assignment was for these compounds and while some proposed fragmentation data is provided, for some compounds, it is not clear how these were derived. Standard compounds should have been used to validate at least some of the key metabolites identified.

It does not appear that elution time was factored in when identifying the compounds, for example one of the identified compounds (PC16:1/2:0) was significantly associated with diabetes. The identification may be wrong as elution time was 0.7minutes whereas compounds such as lysoPC 17:1 eluted at 1.39 minutes. It's also very unlikely that DG's (43:6, 48:6) would elute <2minutes next to the lyso phospholipids. Here again standards would be very helpful.

Unfortunately although this study has a lot of potential to provide interesting insight to metabolism in T2D, the analytical approach and validation do not provide sufficient confidence in the conclusions drawn. I suggest a complete rethink of what is trying to be achieved here and then a careful analysis to address the specific questions under investigation.

Methods

Are the methods appropriate to the aims of the study, are they well described, and are necessary controls included? No

Conclusions

Are the conclusions adequately supported by the data shown? No

Reporting Standards

Does the manuscript adhere to the journal's guidelines on [minimum standards of reporting?](#) Yes

Statistics

Are you able to assess all statistics in the manuscript, including the appropriateness of statistical tests used? Yes, and I have assessed the statistics in my report.

Quality of Written English

Please indicate the quality of language in the manuscript: Not suitable for publication unless extensively edited

Declaration of Competing Interests

Please complete a declaration of competing interests, considering the following questions:

- Have you in the past five years received reimbursements, fees, funding, or salary from an organisation that may in any way gain or lose financially from the publication of this manuscript, either now or in the future?
- Do you hold any stocks or shares in an organisation that may in any way gain or lose financially from the publication of this manuscript, either now or in the future?
- Do you hold or are you currently applying for any patents relating to the content of the manuscript?
- Have you received reimbursements, fees, funding, or salary from an organization that holds or has applied for patents relating to the content of the manuscript?
- Do you have any other financial competing interests?
- Do you have any non-financial competing interests in relation to this paper?

If you can answer no to all of the above, write 'I declare that I have no competing interests' below. If your reply is yes to any, please give details below.

I declare that I have no competing interests

I agree to the open peer review policy of the journal. I understand that my name will be included on my report to the authors and, if the manuscript is accepted for publication, my named report including any attachments I upload will be posted on the website along with the authors' responses. I agree for my report to be made available under an Open Access Creative Commons CC-BY license (http://creativecommons.org/licenses/by/4.0/). I understand that any comments which I do not wish to be included in my named report can be included as confidential comments to the editors, which will not be published.

I agree to the open peer review policy of the journal

To further support our reviewers, we have joined with Publons, where you can gain additional credit to further highlight your hard work (see: https://publons.com/journal/530/gigascience). On publication of this paper, your review will be automatically added to Publons, you can then choose whether or not to claim your Publons credit. I understand this statement.

Yes
