# Supplementary material for: Lipidomic profiling reveals distinct differences in plasma lipid composition in healthy, prediabetic, and type 2 diabetic individuals
Source: Gigascience. 2017 May 15;6(7):1–12. doi: 10.1093/gigascience/gix036 (PMC5502363; doi:10.1093/gigascience/gix036)
Supplement: Reviewer-2-Report-(Original-Submission).pdf [file gix036_Reviewer-2-Report-(Original-Submission).pdf]

Reviewer Report

Title: "Lipidomic profiling reveals distinct differences in plasma lipid composition in healthy, prediabetic and type 2 diabetic individuals"

Version: Original Submission **Date:** 11/18/2016

Reviewer name: Piyushkumar Mundra

Reviewer Comments to Author:

Authors have performed untargeted lipidomic profiling of 293 subjects having Type 2 diabetes (T2D), prediabetes, or normal glucose tolerance (NGT). This study identifies 28 features that could distinguish T2D subjects from NGT.

First, the title suggests a progressive change in lipid profiles from NGT to T2D. However, I believe this could only be achieved if subjects are followed over the period of time and lipid profiling is performed at various time points. With a cross-sectional cohort like this, it could not be termed as progressive changes. At the best, this demonstrates the differences in lipid profiles between NGT, prediabetes, and T2D.

Authors have used random forest classifier to identify the features and build the classifier. They have randomly divided the data into two groups, for testing and validation. However, more thorough analysis will be based on the k-fold external cross-validation, where feature selection is integrated within cross-validation. Any single random split could provide biased results.

KW-test and Spearman's correlation analysis doesn't take into account the confounders. I would suggest authors to use GLM analysis for all the features adjusting for suitable covariates, not just for the selected features.

Some important references are missing, for example Wong et al PLoS ONE 8(10): e76577. A discussion on previously identified features for T2D classification is also important.

Methods

Are the methods appropriate to the aims of the study, are they well described, and are necessary controls included? Yes

Conclusions

Are the conclusions adequately supported by the data shown? Yes

Reporting Standards

Does the manuscript adhere to the journal's guidelines on [minimum standards of reporting?]() Yes

Statistics

Are you able to assess all statistics in the manuscript, including the appropriateness of statistical tests used? No, and I do not feel adequately qualified to assess the statistics.

Quality of Written English

Please indicate the quality of language in the manuscript: Needs some language corrections before being published

Declaration of Competing Interests

Please complete a declaration of competing interests, considering the following questions:

- Have you in the past five years received reimbursements, fees, funding, or salary from an organisation that may in any way gain or lose financially from the publication of this manuscript, either now or in the future?
- Do you hold any stocks or shares in an organisation that may in any way gain or lose financially from the publication of this manuscript, either now or in the future?
- Do you hold or are you currently applying for any patents relating to the content of the manuscript?
- Have you received reimbursements, fees, funding, or salary from an organization that holds or has applied for patents relating to the content of the manuscript?
- Do you have any other financial competing interests?
- Do you have any non-financial competing interests in relation to this paper?

If you can answer no to all of the above, write 'I declare that I have no competing interests' below. If your reply is yes to any, please give details below.

I declare that I have no competing interests.

I agree to the open peer review policy of the journal. I understand that my name will be included on my report to the authors and, if the manuscript is accepted for publication, my named report including any attachments I upload will be posted on the website along with the authors' responses. I agree for my report to be made available under an Open Access Creative Commons CC-BY license (http://creativecommons.org/licenses/by/4.0/). I understand that any comments which I do not wish to be included in my named report can be included as confidential comments to the editors, which will not be published.

I agree to the open peer review policy of the journal

To further support our reviewers, we have joined with Publons, where you can gain additional credit to further highlight your hard work (see: https://publons.com/journal/530/gigascience). On publication of this paper, your review will be automatically added to Publons, you can then choose whether or not to claim your Publons credit. I understand this statement.

Yes
