# Supplementary material for: Lipidomic profiling reveals distinct differences in plasma lipid composition in healthy, prediabetic, and type 2 diabetic individuals
Source: Gigascience. 2017 May 15;6(7):1–12. doi: 10.1093/gigascience/gix036 (PMC5502363; doi:10.1093/gigascience/gix036)
Supplement: Reviewer-3-Report-(Original-Submission).pdf [file gix036_Reviewer-3-Report-(Original-Submission).pdf]

Reviewer Report

Title: "Lipidomic profiling reveals distinct differences in plasma lipid composition in healthy, prediabetic and type 2 diabetic individuals"

Version: Original Submission **Date:** 12/2/2016

Reviewer name: Peter Li

Reviewer Comments to Author:

The authors have submitted a Research article which reports on a metabolomics study using liquid chromatography mass spectrometry to look into the differences in lipid profiles from 293 East Asian people with pre-diabetes (81), type 2 diabetes (T2D) (114) and individuals with normal glucose tolerance (NGT) (98). The lipid profiles of individuals with pre-diabetes and T2D were similar to each other but different to people with NGT.

I believe that the data set generated by the authors of this study can be useful to the scientific community for understanding the abnormal lipid metabolism associated with diabetic disease. However, there are a number of issues with the manuscript and the work it describes before it can be published in GigaScience:

1. The title seems misleading since it contains the word, "progressive". The use of this word suggests a longitudinal study in which the lipid profiles of the 293 individuals have been tracked over a period of time. In fact, the study described in the manuscript is a cross-sectional study in which the lipid profiles of individuals have been identified at a point in time when they either have NGT, pre-diabetes or T2D. The title should be re-worded accordingly.

2. The science in the paper is difficult to understand due to grammatical errors present throughout the manuscript. I appreciate that the authors have performed untargeted profiling of lipids associated with diabetes for the first time in the Chinese population - this is a valuable scientific contribution. However, the application of the Random Forest classifier and the association of human phenotypic markers with specific lipid metabolites could be more concisely and clearly reported in their relevant sections of the manuscript. In general, the standard of written English in the manuscript needs to be improved before it can be published.

3. GigaScience has a focus on reproducible science and requires a "Availability of Data and Materials" section in manuscripts which reports where readers can obtain the data, and the analysis tools and scripts used to generate the results reported in the manuscript. This section should be added into a revised manuscript. The text describing that the raw MS data have been deposited in the MetaboLights database should be moved into this section. I appreciate that the study uses commercial software in the

form of Progenesis to normalise MS data, detect peaks and assign them with metabolite identities, and this tool is not be publicly available. This is fine with me but it should be stated. However, other software tools and scripts responsible for generating the univariate and multivariate statistical results should, where possible, be provided and/or deposited in a Github repository.

Peter Li, GigaScience.

Methods

Are the methods appropriate to the aims of the study, are they well described, and are necessary controls included? No

Conclusions

Are the conclusions adequately supported by the data shown? Yes

Reporting Standards

Does the manuscript adhere to the journal's guidelines on minimum standards of reporting? No

Statistics

Are you able to assess all statistics in the manuscript, including the appropriateness of statistical tests used? No, and I do not feel adequately qualified to assess the statistics.

Quality of Written English

Please indicate the quality of language in the manuscript: Not suitable for publication unless extensively edited

Declaration of Competing Interests

Please complete a declaration of competing interests, considering the following questions:

- Have you in the past five years received reimbursements, fees, funding, or salary from an organisation that may in any way gain or lose financially from the publication of this manuscript, either now or in the future?
- Do you hold any stocks or shares in an organisation that may in any way gain or lose financially from the publication of this manuscript, either now or in the future?
- Do you hold or are you currently applying for any patents relating to the content of the manuscript?
- Have you received reimbursements, fees, funding, or salary from an organization that holds or has applied for patents relating to the content of the manuscript?
- Do you have any other financial competing interests?
- Do you have any non-financial competing interests in relation to this paper?

If you can answer no to all of the above, write 'I declare that I have no competing interests' below. If your reply is yes to any, please give details below.

I am employed by GigaScience as a Data Organisation Manager.

I agree to the open peer review policy of the journal. I understand that my name will be included on my report to the authors and, if the manuscript is accepted for publication, my named report including any attachments I upload will be posted on the website along with the authors' responses. I agree for my report to be made available under an Open Access Creative Commons CC-BY license (http://creativecommons.org/licenses/by/4.0/). I understand that any comments which I do not wish to be included in my named report can be included as confidential comments to the editors, which will not be published.

I agree to the open peer review policy of the journal

To further support our reviewers, we have joined with Publons, where you can gain additional credit to further highlight your hard work (see: https://publons.com/journal/530/gigascience). On publication of this paper, your review will be automatically added to Publons, you can then choose whether or not to claim your Publons credit. I understand this statement.

Yes
